# Accurate determination of CRISPR-mediated gene fitness in transplantable tumours

**DOI:** 10.1038/s41467-022-31830-2

**Published:** 2022-08-04

**Authors:** Peter Eirew, Ciara O’Flanagan, Jerome Ting, Sohrab Salehi, Jazmine Brimhall, Beixi Wang, Justina Biele, Teresa Algara, So Ra Lee, Corey Hoang, Damian Yap, Steven McKinney, Cherie Bates, Esther Kong, Daniel Lai, Sean Beatty, Mirela Andronescu, Elena Zaikova, Tyler Funnell, Nicholas Ceglia, Stephen Chia, Karen Gelmon, Colin Mar, Sohrab Shah, Andrew Roth, Alexandre Bouchard-Côté, Samuel Aparicio

**Affiliations:** 1 Department of Molecular Oncology, BC Cancer, Vancouver, BC Canada; 2 Computational Oncology, Department of Epidemiology and Biostatistics, Memorial Sloan Kettering Cancer Center, New York, NY 10065 USA; 3 Department of Medical Oncology, BC Cancer, Vancouver, BC Canada; 4 Department of Diagnostic Radiology, BC Cancer, Vancouver, BC Canada; 5 Department of Pathology and Laboratory Medicine, University of British Columbia, Vancouver, BC Canada; 6 Department of Computer Science, University of British Columbia, Vancouver, BC Canada; 7 Department of Statistics, University of British Columbia, Vancouver, BC Canada; 8 Present Address: AbCellera Biologics Inc., Vancouver, BC Canada; 9 Present Address: British Columbia Institute of Technology, Vancouver, BC Canada

**Keywords:** Breast cancer, Target validation, Cancer genomics, Tumour heterogeneity

## Abstract

Assessing tumour gene fitness in physiologically-relevant model systems is challenging due to biological features of in vivo tumour regeneration, including extreme variations in single cell lineage progeny. Here we develop a reproducible, quantitative approach to pooled genetic perturbation in patient-derived xenografts (PDXs), by encoding single cell output from transplanted CRISPR-transduced cells in combination with a Bayesian hierarchical model. We apply this to 181 PDX transplants from 21 breast cancer patients. We show that uncertainty in fitness estimates depends critically on the number of transplant cell clones and the variability in clone sizes. We use a pathway-directed allelic series to characterize Notch signaling, and quantify *TP53* / *MDM2* drug-gene conditional fitness in outlier patients. We show that fitness outlier identification can be mirrored by pharmacological perturbation. Overall, we demonstrate that the gene fitness landscape in breast PDXs is dominated by inter-patient differences.

## Abstract (web summary)

Gene fitness and essentiality analyses using in vivo cancer models are challenging due to multiple confounders. Here, the authors develop a quantitative approach to study CRISPR-transduced patient-derived xenografts, which they use to analyse in vivo gene fitness in breast cancers and the biological features that influence uncertainty in fitness estimation.

## Introduction

Triple negative breast cancers (TNBC) are a heterogenous group of diseases, defined by the absence of detectable estrogen and progesterone receptors (*ESR1*, *PGR*), non-amplified/normally expressed Her2 receptor (*ERBB2*), and characterized by aggressive clinical behaviour and poor prognosis. Somatic mutations, cell of origin and germline variation contribute to the as yet poorly defined phenotypic variation of TNBC^1^. Large-scale sequencing studies have provided the basis for molecular approaches to identify TNBC subsets with similar underlying biology. These include subsets defined by transcript profile, including a basal expression subtype^2,3^, by the activity of recurrently dysregulated signaling pathways, by activity and deficiency in DNA repair pathways (notably double strand break repair)^4^, by combined expression and copy number profile^5,6^, by clonal diversity and kinetics^7^, and by immune and stromal features in the tumour environment^8^. Insights from these classification approaches are already guiding patient stratification for treatment with novel targeted agents^9^. Nonetheless, the heterogeneity of patient response highlights the need for more direct and quantitative approaches to understand the fitness of genes within the complex genomic and epigenomic backgrounds that characterize these cancers.

Gene fitness estimates can be made at medium to high throughput by quantification of cellular growth phenotypes under defined perturbations, for example treatment with drug panels, transcript inhibition through RNA interference^10^ or gene targeting by CRISPR-KO or CRISPRi^11^. In CRISPR-based approaches, integration of a vector including a single guide RNA (sgRNA) delivers both a targeted genetic perturbation in trans and a heritable genetic marker that can be used to identify the perturbation in the cell’s progeny. This underlies pooled library screening approaches, in which cell populations transduced with diverse libraries of CRISPR guides are jointly propagated under competitive conditions, and the relative growth of subsets bearing each perturbation quantified by next generation sequencing. This approach has been widely applied to cancer cell lines propagated in vitro, identifying genes broadly essential for cell growth and survival, genes with differential fitness in lines of particular origin or mutational background, and combinatorial gene-gene and/or gene-drug interactions^12,13^.

Extending these approaches to in vivo models such as patient-derived xenografts (PDX) offers the potential to characterize gene fitness in physiologically-relevant settings and in mutationally and clonally complex tumour backgrounds. However, aspects of xenograft biology pose notable challenges. Firstly, tissue transplantation introduces a cellular survival bottleneck, with tumours representing the clonal progeny of limited numbers of input cells^14,15^. This affects statistical strength by putting a ceiling on the number of independent datapoints (clonally-expanding cell populations) sampled. Secondly, there is considerable heterogeneity in the cellular output of transplanted cells that successfully propagate to the final tumour, with relative clone sizes spanning 3-4 orders of magnitude in breast cancer PDX models^16,17^. This can add considerable noise when assaying signals from introduced perturbations, since final clone populations sizes compound their effects with the cellular growth potential of each transplanted cell.

A strategy to address heterogeneity of cellular output is to incorporate degenerate cellular barcodes (unique molecular identifiers, UMIs) into experimental vectors. Each transduced starting cell then receives both a functional perturbation via the sgRNA and a genetic marker via the UMI, which are passed to the cell’s clonal progeny^18,19^. Statistical approaches informed by joint sgRNA-UMI counts can improve signal detection in the screen, for example by reducing confounding effects of clones of outlying size. The approach has been applied to pooled screens in 3D organoid culture^20^ and mouse tumour models^21–23^, which are characterized by heterogenous cellular output.

Extending this concept, we develop here joint UMI and sgRNA-encoding vectors and use these in conjunction with a Bayesian statistical model informed by clone size distribution to make quantitative inference of gene fitness in PDXs. We demonstrate statistical power to resolve in vivo gene fitness differences and driving pathway mechanisms in a diverse set of 181 CRISPR-transduced tissue xenotransplants from 21 different breast PDX lines.

## Results

### Factors influencing quantitative gene fitness measurements in vivo

We sought to develop a rigorous quantitative approach to estimation of fitness via in vivo population competition experiments. It is well known that the total progeny arising from in vivo proliferation of single tumour cells in primary human transplant experiments is highly variable^16,17^. The resulting distributions of clone sizes have a heavy tailed form, in which small numbers of cells may contribute disproportionately to the total population of cells as the system evolves in time. In experiments which rely on sampling from clonal populations of this form, the accuracy and precision of estimates is strongly related to the numbers of clones sampled, with loss of precision when sampling is sparse.

**Fig. 1:**
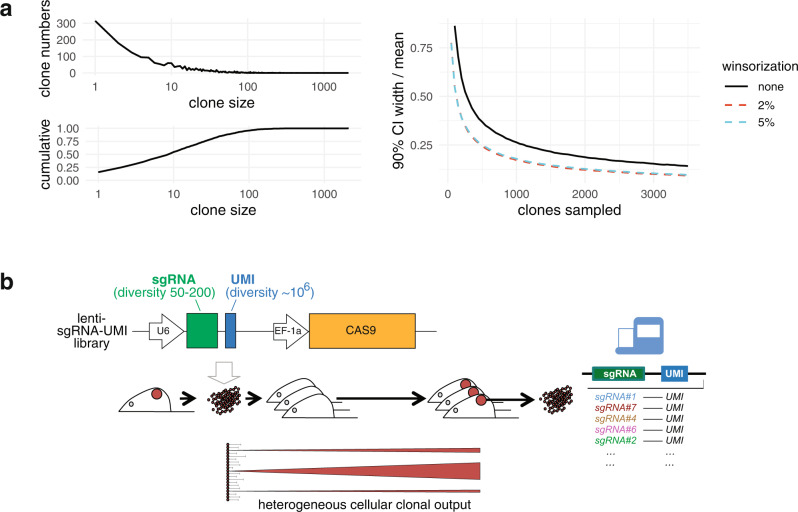
Quantitative assessment of gene fitness in vivo. **a** Sampling from a heavy-tailed clone distribution. Left panels show the size distribution (upper) and cumulative size distribution (lower) of clones in a barcoded PDX tumour. Right panel shows the decrease in uncertainty in the sampled population mean clone size when clones are sampled in increasing numbers. Solid line = arithmetic mean, dashed lines = winsorized mean. **b** Experiment schematic. PDX cells are transduced ex vivo with a CAS9-sgRNA-UMI lentiviral library, and transplanted into recipient mice. Resulting tumours are harvested, and joint sgRNA-UMI identities determined by targeted amplicon sequencing of bulk DNA. Schematic lower panel highlights that many initially transplanted cells are not represented in the final tumour, and those transplanted cells that do are represented with heterogenous numbers of clonal progeny. Source data are provided as Source Data files.

The strength of these effects was evident from a simulation in which clones were randomly sampled repeatedly from a published breast cancer PDX data set^16^ (Fig. 1a, left panel). As the sample size falls below ~500–1000 clones, the heavy-tailed size distribution results in increasingly greater uncertainty over the mean clone size (Fig. 1a, right panel, 90% credible interval (CI) of mean estimate). The effects of sparse sampling can be partially offset by re-scaling the extreme outliers (winsorization, Fig. 1a, right panel), however the general form persists.

We thus reasoned that rigorous quantification of fitness requires (i) a method of estimating clone size for each perturbation in the population (in this case, for each sgRNA molecule introduced into a single cell during tumour transplantation); (ii) appropriate sizing of the sgRNA library, given the clone numbers and variability in clone size in the tissues/tumours being sampled; (iii) minimizing distortion of clone number and size during experimental manipulation; and (iv) a statistical model for estimating fitness and variance from multiple measurements of a sample population, accounting for the form of the clone size distribution.

### Population size encoding of CRISPR-transduced transplant cell progeny through UMI tags

First, to measure sgRNA clone sizes, we introduced a degenerate barcode sequence (unique molecular identifier, UMI)^24^ into a Cas9-containing lentiCRISPRv2 vector (Figs. 1b and S1a, b). In this arrangement, each vector molecule is coded with a unique sgRNA-UMI sequence that can be read through as a single molecule in sequencing. We verified the compatibility of UMI encoding with CRISPR-sgRNA function. Plasmids containing a 27 nucleotide UMI sequence insert either upstream or downstream of an sgRNA targeting enhanced green fluorescent protein (eGFP) demonstrated comparable silencing efficiency of the target protein as the unmodified vector when transfected into an eGFP-expressing cell line (Fig. S1c). Downstream UMI-containing plasmid packaged efficiently into lentiviruses (typical functional titres 0.5 × 10^8^–3 × 10^8^ infectious units/mL). Pooled CRISPR competitive growth assays with the modified vector carried out in 2D culture in breast cell lines (luminal-type MCF7 and normal-derived 184htert *TP53*^–/–^ *BRCA2*^–/–^) showed expected depletion of sgRNAs targeting known essential and growth-promoting genes (e.g. *PLK1*, *PLK4*, *CHEK1*, *MTOR*, *MYC*) (Fig. S1d). Together, these indicate that the addition of the UMI sequence is compatible with normal function of the lentivirally-delivered CRISPR-Cas9 system.

Second, a key initial factor to avoid biased starting conditions is the size of the sgRNA library relative to the number of clones and variability in clone size. We estimated that transplants of 1 × 10^6^–1 × 10^7^ cells would yield tumours of ~1 × 10^4^–1 × 10^6^ clones, given reported breast PDX clone-initiating frequencies of 1 per 1 × 10^1^–1 × 10^4^ cells^16,17^. This suggests that screens employing libraries of ~10–1000 different guides can be adequately powered to achieve clone numbers of 500–1000 per guide.

Based on this, we cloned a library of sgRNAs targeting growth factor receptors, hormone receptors, key members of PI3-kinase, integrin, DNA damage, RAS-MAPK, cell-cycle control, Notch, Wnt, Hedgehog, Hippo, hypoxia and autophagy pathways (168 sgRNAs targeting 56 genes in triplicate plus 24 non-targeting sgRNAs, 192 sgRNAs total, Table S1) into the vector. Targeted amplicon sequencing confirmed a broad joint distribution of sgRNAs and UMIs in the library (Fig. S1e, f).

Details of transduction and PCR sequencing of libraries are in Methods. Briefly, PDX tissue was disaggregated quickly to small organoids, avoiding lengthy enzymatic dissociation processes that can reduce viable cell numbers. Following transduction with virus for four hours ex vivo, tissue was transplanted into recipient mice, allowed to grow tumours of at least 0.1 cm^3^ in size over a period of weeks, then harvested for DNA extraction (Fig. 1b). For most experiments we used a viral dose of 8–10 × 10^6^ infectious units per transplant (range 2.8–24.6 × 10^6^ infectious units in entire study, Table S2). A PCR amplicon spanning both sgRNA and UMI was sequenced (Illumina MiSeq or NextSeq) to a depth of ~2 × 10^6^ reads per tumour. sgRNA and UMIs were extracted from sequence data, filtered for sequence quality, with all reads with matched sgRNA and UMI vector sequences considered as individual clones of an sgRNA-UMI introduced into a cell. To reduce PCR and sequencing error inflation of sgRNA-UMI clone measurements, a round of in silico clone aggregation was carried out using the UMI-Tools Directional algorithm^25^.

### Heterogeneity of clone size in sgRNA-UMI PDX transplants

**Fig. 2:**
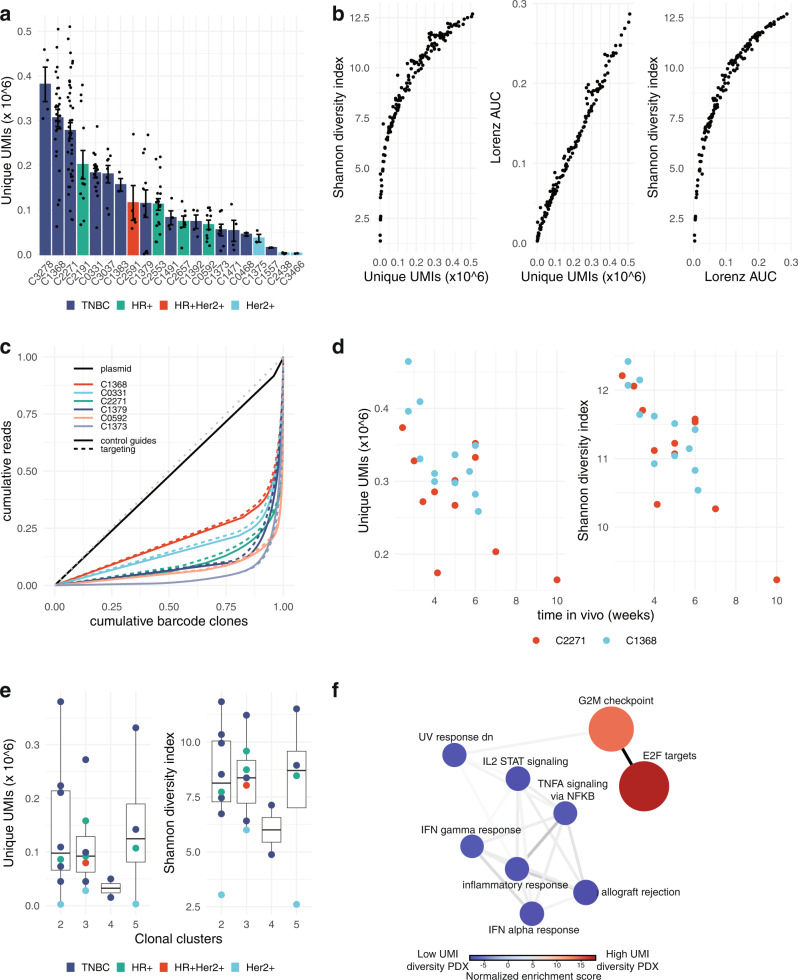
Transplant clone diversity in breast cancer PDXs. **a** Numbers of unique sgRNA-UMI clones per million reads in transduced tumours grown in 21 PDX lines (bars show mean + /− SEM for each PDX line, individual tumour datapoints overlaid). **b** Diversity measures in transduced PDX tumours. Panels compare sgRNA-UMI clone number with two measures of diversity; the Shannon diversity index and the area under a Lorenz curve plot of cumulative sequence reads against cumulative clone numbers. All measures are evaluated per million sequence reads. Lower clone numbers per tumour are strongly associated with a greater heterogeneity in clone sizes. **c** Lorenz curves of cumulative sequence reads against cumulative clone numbers, illustrating clone size heterogeneity in sample tumours from six PDX lines, and in the initiating sgRNA-UMI library plasmid (black). Solid and dotted lines show data from control and gene-targeting guide subsets. A greater deviation from the x = y diagonal indicates a greater unevenness in clone sizes. **d** Reduced diversity with passaging time. Panels show clone numbers (left) and Shannon diversity index (right) for replicate transplants from two PDX lines harvested at variable timepoints after transplantation. **e** Transplant clone diversity is not associated with genomic clonal diversity. Panels show clone numbers (left) and Shannon diversity index (right) against the number of genomic clonal clusters inferred from bulk WGS by population structure model PyClone-VI in 21 PDX lines (sequence from one PDX tumour analyzed per line). Boxplot lines at 25th, 50th and 75th centiles, vertical lines extend to furthest datapoint within 1.5 interquartile range distance of box limits. **f** GSEA pathway enrichment graph derived from RNAseq transcriptomes from the six highest compared with six lowest sgRNA-UMI diversity PDX lines. Node size indicates the number of genes in curated pathways, from Reactome Hallmark pathway set. Edge line width indicates numbers of genes shared between pathways. High sgRNA-UMI diversity PDX lines show differential activity in cell-cycle pathways, low sgRNA-UMI diversity lines in immune pathways. Source data are provided as Source Data files.

To explore the clonal features of transduced PDX tumours, we first enumerated the number of unique sgRNA-UMI clones measured in transplants from breast cancer PDX lines derived from 21 different patients (Fig. S2). We observed a wide range of clone numbers in individual transplants (90% range: 0.006–0.424 × 10^6^ clones per million reads; 33–2210 per sgRNA). This equated to a greater than 10-fold range in the median in different breast PDX lines (Fig. 2a), consistent with variability found in previous analyses of clone initiation in breast PDX^16,17^.

Comparison of replicate transplants further indicated that sgRNA-UMI clone number is a reproducible property of PDX lines (Fig. 2a). Within our panel of PDX lines, TNBCs and hormone receptor positive subsets included medium and high clone number examples, whereas clone number was low (fewer that 10^5^ sgRNA-UMIs per 10^6^ reads) in the three hormone receptor negative Her2 positive lines (Fig. 2a).

We reasoned that low sgRNA-UMI clone number in certain PDX lines could reflect heavy-tailed clone size distributions, with sizeable proportions of total tumour cell mass represented by small proportions of transplant clones. We therefore compared sgRNA-UMI clone number with two different measures of diversity; Shannon diversity index (SDI), a composite entropy measure of evenness of clone size distribution and total clone number; and the area under the Lorenz curve plot of cumulative reads against cumulative clones, a function of clone size distribution alone (Fig. 2b, c, Supplementary Methods 1.2). We found clone number to be positively correlated with SDI and with homogeneity in clone size (Fig. 2b), with tumour composition ranging from extremes of large numbers of more evenly-sized clones (high entropy) to small numbers of more heterogeneously-sized clones (low entropy). We did not observe outcomes with large numbers of evenly-sized clones. These relationships were maintained independent of whether cells received a neutral sgRNA or a gene-targeting sgRNA (Fig. 2c), indicating that clone number and size distribution are intrinsic properties of the PDX transplant cellular regenerative process, not solely consequences of introduced gene perturbations.

We found that extended propagation time in vivo led to a decrease in overall diversity (Fig. 2d). To maintain favourable sampling diversity, we therefore harvested tumours at relatively small size (~0.1–0.15 cm^3^, typically, 3–8 weeks’ growth in vivo) in subsequent competitive fitness experiments. Transplant clone diversity did not show a correlation with the extent of genomic clonal diversity within PDXs, as measured by the number of mutational clusters inferred from bulk WGS by Bayesian population model PyClone-VI^26^ (Fig. 2e).

Finally, to examine biological features contributing to sgRNA-UMI clone diversity, we used gene set enrichment analysis^27^ on bulk RNAseq to identify pathways with differential activity in PDX lines with higher compared with lower SDI. Within the Reactome Hallmark pathway set (www.reactome.org), PDX lines with higher diversity were characterized by elevated activation of cell cycle-related pathways, notably G2M checkpoint and E2F targets (Fig. 2f). In contrast, PDX lines with lower diversity showed elevated activation of allograft rejection and inflammatory pathways, notably interleukins and interferons (Fig. 2f). This suggests interaction between transplanted tumour cells and the host innate immune system as a possible mechanism to explain the lower number of engrafting transplant clones in these PDX lines. Elevated cytokine response might also reflect response to endogenous DNA damage in the low sgRNA-UMI diversity subset. However, we did not find a correlation between sgRNA-UMI diversity and the overall burden of somatic single nucleotide variants in PDX lines to support this interpretation (Fig. S3a). Similarly, we did not observe strong correlation with genome-wide mutational signatures^28^, with only the SBS8 signature^29^ showing a significantly non-zero regression slope coefficient (*p* = 0.008) with sgRNA UMI diversity but with low overall coefficient of determination (*r*^2^ = 0.33) (Fig. S3b). Thus, measured clone diversity in library transplant experiments appears to be driven by a number of biological features, including proliferating-cell content of the transplanted material and activity of immune-pathway cytokines in tumour cells.

Taken together the key elements required for population fitness measurements were identified as, appropriately scaled perturbation library size; short transduction times with minimal tumour manipulation; early harvesting of tumour transplants; and evaluation of intrinsic clone diversity capacity of each PDX.

### Fitness inference informed by UMI distributions and a Bayesian hierarchical mixture model

**Fig. 3:**
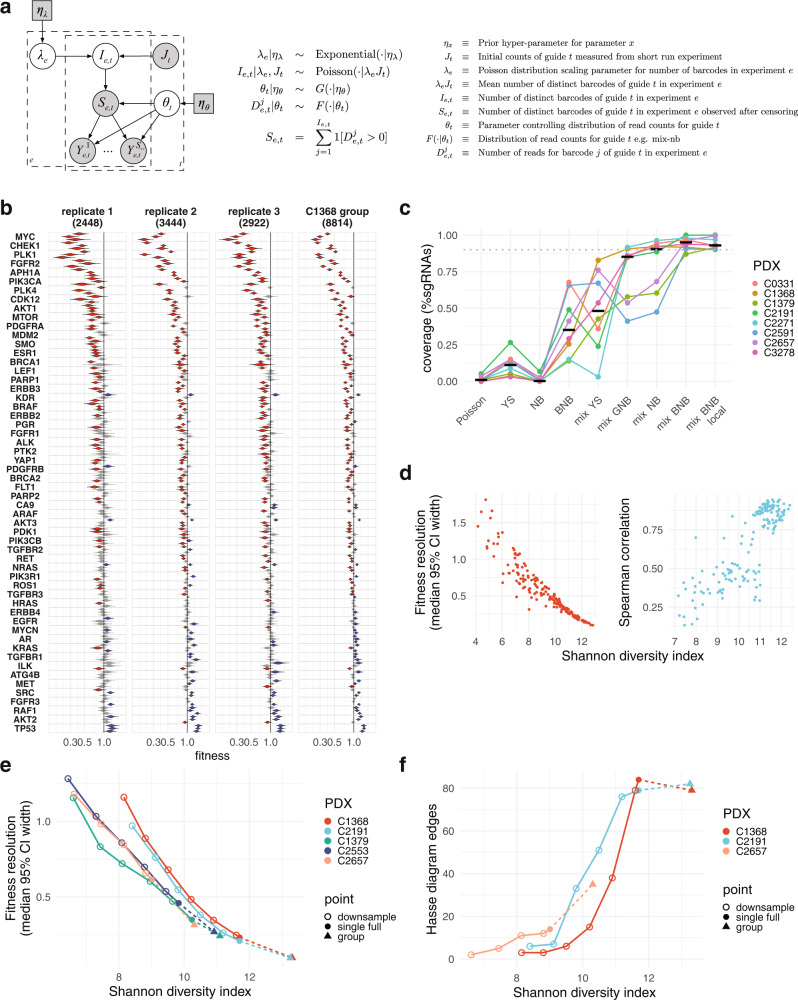
Bayesian model for UMI-informed fitness inference in pooled screens. **a** Bayesian probabilistic graphical model schematic. **b** Posterior distributions of guide fitness for three biological replicate transplants from C1368 transduced with a 192-guide library targeting 56 signaling genes. Right hand panel displays posterior distributions for a group inference run using data from the three replicates. Mix-NB model is used. Figures above each panel indicate mean sgRNA-UMI clones per guide. Guide distributions coloured red (low fitness) and blue (high fitness) indicate estimates that differ from neutrality (fitness = 1) with false discovery rate <0.05 (Benjamini-Hochberg method). **c** Bayesian goodness of fit test for different count likelihood models. Improved fit is obtained using mixtures of two distributions. (YS = Yule Simon, NB = negative binomial, BNB = beta negative binomial, mix = weighted linear mix of 2 distributions of the same model type with same mixture weight for all sgRNAs in each sample, except mix-BNB local in which a separate mixture parameter is used for each sgRNA.) **d** Higher sgRNA-UMI diversity is associated with screens with narrower credible intervals and closer correlation between biological replicates. Left panel shows the median fitness credible interval width against Shannon diversity. Right panel shows Spearman rank correlation of guides against mean Shannon diversity in pairwise comparisons between biological replicates. **e** Effect on screen resolution of reducing sgRNA-UMI numbers through in silico clone subsampling (unfilled circles), or increasing numbers by combining biological replicate datasets (triangles). Data shown for starting datasets from 5 different PDX lines (solid circles). **f** Effect on the number of guide pairs with differentially resolved fitness, as sgRNA-UMI numbers are varied by in silico clone subsampling (unfilled circles) or combining biological replicate datasets (triangles). Resolved pairs are Hasse plot edges connecting guide pairs with non-overlapping 95% credible intervals (fitness *a* < *b*, and no *c* such that *a* < *c* < *b*). As fewer sgRNA-UMIs are sampled, the power to resolve fitness pairwise differences between guides decreases. Data shown for starting datasets from 3 different PDX lines (solid circles). Source data are provided as Source Data files.

We anticipated that PDX line differences in cellular regeneration properties would translate into differences in the statistical power of pooled CRISPR screens, notably a reduction in power in PDX lines generating tumours comprising fewer clones. To control for PDX specific effects we developed a modeling approach using jointly measured sgRNA-UMI data to infer the fitness impact of sgRNA perturbations introduced synchronously into a PDX. We developed a hierarchical Bayesian model to jointly analyze the observed UMI and sgRNA read count data, which allowed us to quantify the uncertainty in fitness estimates via credible intervals obtained from the posterior distribution (Fig. 3a, Supplementary Methods 1.5). To account for differences in the prevalence of sgRNAs within each library (Fig. S1f), sequence read count data from tumours propagated in mice (termed final counts, *D*^*j*^_*t*_) are modelled along with read counts from the plasmid vector library used for packaging into lentivirus used to transfect (termed initial counts, *I*_*t*_). To increase statistical power, we included the ability to jointly analyze multiple replicates within the modeling framework. Winsorization (which here revalues the top 2% of the posterior distribution to the 98th centile point) was used to produce robust estimators that reduce the impact of clones of outlying size (Fig. S4c). The model returns the posterior distribution of the winsorized mean of cellular output as the sgRNA fitness estimator (Fig. 3b, left 3 panels represent replicate transplants from a high diversity PDX line, right panel shows the grouped analysis, Supplementary Data 1). Fitness in this modeling approach is thus a measure of the relative expected cellular output of cells transduced with different guides, after lowering of the impact of outlying large clones through winsorization.

We used a Bayesian goodness of fit procedure to evaluate the performance of different generative functions modeling distributions of this type (Supplementary Methods 1.5.6). The procedure measures the percentage of sgRNAs for which observed mean clone size falls within the modeled 90% posterior credible interval (expected to be close to 90% of the sgRNAs if the fit is good). Likelihoods based on mixture models (e.g. sum of two negative binomial distributions with different means and dispersions) resulted in improved goodness-of-fit when applied to datasets representing a range of sgRNA-clone diversity (Figs. 3c and S4a). In contrast, likelihoods based on a single distribution functions (e.g. Poisson, negative binomial, beta negative binomial, Yule-Simon) had lower flexibility to fit to the form of empirical distributions, resulting in lower coverage of observed means within modeled credible intervals. While goodness-of-fit and marginal likelihoods varied with the use of different generative functions (Supplementary Methods 1.5.6), we note that the median Bayesian fitness estimates were broadly similar, with the exception of the poorly-fitting Poisson model (Fig. S4b). For subsequent analysis, we chose the mix-NB model (mixture of two negative binomial distributions). This distribution has defined moments, making model interpretation more straightforward, and also has faster execution time compared with mix-BNB, which has slightly higher marginal likelihood and overall goodness-of-fit. We also note that the better fit of mixture models is consistent with the presence of more than one biologically distinct cell type in the initiating transplants.

For comparison, we also generated sgRNA-UMI-informed fitness estimates and confidence intervals using a frequentist methodology based on a ratio statistic and the Delta method (Supplementary Methods 1.4). While Bayesian and frequentist methodologies generated broadly similar rankings of guide fitness in single data set analyses (Fig. S4b, right panels), the Bayesian approach was better suited to leveraging of replicates in grouped analyses in which data and a subset of the model parameters were shared between multiple data sets from biological replicate transplants (Fig. 3b, right panel; note the narrower posterior distributions compared with single data set distribution in three left panels).

While our modeling approach assumes that each transduced cell receives a single viral integration, it is known that transduction of cell populations with viruses results in a subset of cells receiving two or more integration events. Multiple integration events may lead to some overestimation of clone number, since sequences from the progeny of these cells are counted in more than one clone. Nonetheless, this effect should affect guides uniformly and thus be neutral for relative fitness estimates. We investigated this with a model version in which initial cell transduction frequency is assumed to follow a Poisson distribution (Supplementary Methods 1.5.4). This model showed a negligible increase in marginal likelihood in our data sets relative to the single infection mix-NB model (Supplementary Methods 1.5.7), implying that a multiple infection model does not bring significant benefits in fitting the data. We also carried out transduction experiments in which parallel transplants received viral doses varying over a four-fold range. Fitness estimates were well correlated (*r*^2^ 0.766–0.808, Fig. S5a), and only small variation in credible interval width was observed over the viral dosing range (median 95% interval 0.219, 0.209, 0.208 at doses of 5, 10, 20 × 10^6^ infectious units). For simplicity, we therefore settled on the single infection model version for subsequent analyses.

We used variants of the signaling library that included negative control guides that are non-genome targeting (no matching sequence in human genome) or non-gene-targeting (matching sequence in the human genome, at least 5 kbp distant from any coding region). Comparison of these offers an estimate of the magnitude of non-target-specific growth inhibition from Cas9-induced genome cutting, since the latter but not the former controls are expected to target any locus. Consistent with this, non-genome-targeting control guides registered higher fitness compared with non-gene-targeting controls (Fig. 5a, *ctl* and *ctl*_*t*_, respectively), with a mean difference in net growth of 0.30. A copy number dependent non-gene-specific negative effect on cellular proliferation on CRISPR cutting has been previously documented in cell line studies^12^.

We next examined the relationship between clonal diversity in PDX tumours and the power to resolve fitness differences between sgRNA targets. Using the mean 95% Bayesian credible interval width as a proxy for the resolution of a pooled PDX screen, we observed a strong inverse correlation between clonal diversity and resolution (Fig. 3d, left panel). This was also reflected in a greater reproducibility of fitness ranking between replicate transplants from higher diversity compared with lower sgRNA-UMI diversity tumours (Fig. 3d, right panel). The inverse relationship between clone diversity and screen resolution was also seen when reducing UMI numbers by sub-sampling clones from single datasets, or by increasing number by combining biological replicate datasets (Fig. 3e). As a graphical representation of fitness differences resolved at significance between sgRNAs, we generated partially ordered sets (posets), visualized using Hasse diagrams, which show minimal sets of edges connecting sgRNA nodes with non-overlapping 95% Bayesian credible intervals (fitness *a* < *b*, and such that there are no *c* such that *a* < *c* < *b*) (Fig. S6). Here, sampling of fewer clones resulted in progressively simpler graphs resolving fitness differences between fewer sgRNA pairs (Figs. 3f and S6).

Overall, these observations demonstrate that the power to resolve in vivo fitness differences between genes is critically dependent on clone diversity, derived from the number of transplant clones in a regenerated tumour and the heterogeneity in their sizes. These are biological properties of PDXs, varying considerably between lines derived from different patient tumours.

### Intrinsic between-patient variation dominates primary breast cancer gene fitness in vivo

Having established Bayesian fitness measures from sgRNA-UMI clones in vivo, we set out to examine sources of variation of single gene fitness among different patient tumours for the functions of PI3-kinase, integrin, DNA damage, RAS-MAPK, cell-cycle control, Notch, Wnt, Hedgehog, Hippo, hypoxia and autophagy pathways represented in the sgRNA library. We conducted multiple technical replicate transplant experiments (*n* = 3–6) for 15 patients (10 TNBCs) from the 21 patient series (6 excluded due to low sgRNA-UMI diversity), contrasting different passages and anatomical sites of transplant of individual patient tumours.

**Fig. 4:**
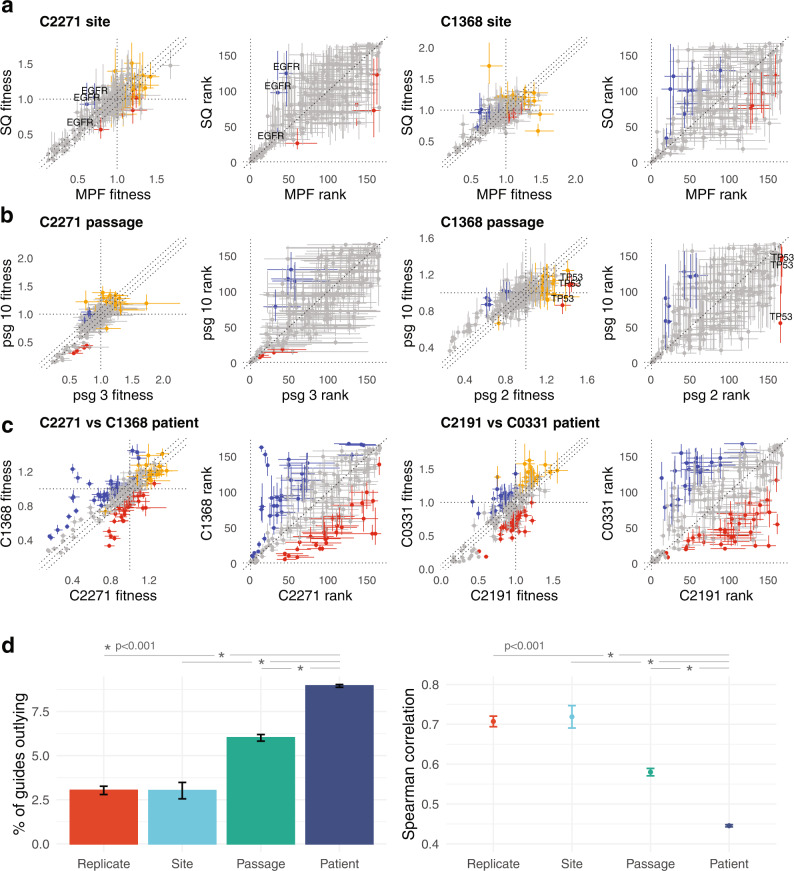
Between-patient variation dominates in vivo gene fitness. **a** Two examples comparing guide fitness measurements (median, bars = 95% CI) between transplants of the same TNBC PDX material into subcutaneous (SQ) or mammary fat pad (MFP) sites. Red, blue: guide outliers between the two conditions satisfying criteria related to both fitness and rank difference, and with more than one outlying guide targeting the same gene (Supplementary Methods). Orange: non-targeting control guides, shown in fitness plots. (PDX tumours: C2271 *n* = 2, 1 for MFP, SQ; C1368 *n* = 2, 1 for MFP, SQ.) **b** Two examples comparing guide fitness measurements (median, bars = 95% CI) between transplants of an early and late passage from the same TNBC PDX line. Criteria for identifying outlying guides as in **a**. (PDX tumours: C2271 *n* = 3, 3 for early, late; C1368 *n* = 3, 2 for early, late.) **c** Two examples comparing guide fitness measurements (median, bars = 95% CI) between transplants of PDX lines from different patients. Criteria for identifying outlying guides as in **a**. (PDX tumours: *n* = 3, 3, 4, 3 for C2271, C1368, C2191, C0331.) **d** Left panel shows the percentage of guides satisfying outlying criteria for all pairs of datasets that represent biological replicates; site comparisions; early vs late passage comparisons; and comparisions between different patient PDX lines. Right panel shows the Spearman rank correlation of guide fitness for the same sets of comparisons. Bars display mean + /− SEM (*p* < 0.001 two-sided for all pairs, except for Replicate-Site, in both panels). Both metrics show greatest dissimilarity in fitness for comparisons involving different patients. (*n* = 249, 30, 496 and 4624 pairwise comparisons for replicate, site, passage, patient). Source data are provided as Source Data files.

First, to assess gene fitness in different transplant sites, we compared replicate transplants from 6 different PDX lines grown in separate groups of mice in subcutaneous or inguinal mammary fat pad sites (Fig. 4a). To facilitate comparison between experiments and sgRNA libraries, fitness values were normalized within each dataset such that the 70th centile value among gene-targeting guides was set to a neutral level of 1 (Supplementary Methods 1.6). We applied a stringent set of criteria to identify targeting guides showing statistical and biological significant difference between sites, namely: fitness differential that is non-zero (by 95% posterior credible interval); targeting guides that show statistically significant fitness differential relative to the variability in neutral guides; rank differential that is non-zero (by 95% posterior credible interval); and at least two guides targeting the same gene satisfying these three criteria (Supplementary Methods 1.8). Representative comparison examples are shown in fitness and rank space, with guides satisfying the difference criteria highlighted (Fig. 4a). We did not observe strong site-specific fitness differences, although in four of six patients *EGFR* exhibited modestly lower fitness (~0.4) in the mammary fat pad site than subcutaneous site (Figs. 4a, left two panels and S7). Related pathway *ERBB* family receptors exhibited overall neutral differential fitness, with one patient each exhibiting a growth effect for *ERBB2* and *ERBB3* (Fig. S7). Thus, for all but *EGFR*, site of transplant had little or no effect on the measured fitness ranks.

Polyclonal primary breast cancers evolve in patients and in PDX transplants^30^. To assess the variation in fitness with serial transplant passaging, we compared an early passage (2nd–3rd mouse generation since initiating transplant from patient) with a later passage (6–12th) for three PDX lines (intervening time 15, 18 and 23 months in vivo). We observed relative stability in the rank ordering of guides, implying that biological drivers at early passages remain functional over significant periods of time in vivo (Figs. 4b and S8). Notable outliers included *TP53* in C1368, a TNBC line in which the all three *TP53*-targeting guides showed positive fitness at passage 2 (consistent with wild-type *TP53* status in this line) and neutral fitness at passage 10 (Fig. 4b, right two panels). We examined mutation status and copy number in pseudobulk clonally merged genomes derived from DLP + scWGS^31^ sequenced at the early and late passages. In spite of passage-related copy number clonal evolution, we did not find somatic mutations in or closely related genes to explain this functional shift. We also noted that guide fitness profiles were well correlated between transplants of the same tumour material harvested after varying propagation periods in vivo (*r*^2^ 0.759–0.892, Fig. S5b), indicating that time-dependent fitness within a passage generation was not a strong feature in these experiments.

In contrast with the relative stability in fitness profiles between technical replicates, or variations in site and passage, we observed considerably greater fitness variation when comparing PDX lines originating from different patients (Fig. 4c). These comparisons indicated different functional drivers within the TNBC subset of lines, as well as showing the expected differences in hormonal signaling in ER + lines compared with TNBC (Fig. S9). Quantifying the proportion of genes satisfying the multiple difference criteria across the entire data set (5399 pairwise comparisons) showed a progression of increasing fitness variability from technical replicates or site comparisons to passage comparisons to inter-patient comparisons (Fig. 4d, left panel, Supplementary Methods 1.8). A similar ordering was seen using the Spearman rank correlation of guide fitness in the comparisons (Fig. 4d, right panel), with between-patient variation dominating the fitness landscape. Taken together, by far the greatest source of variation in fitness among genes assessed in vivo arises from intrinsic differences between primary breast cancers.

**Fig. 5:**
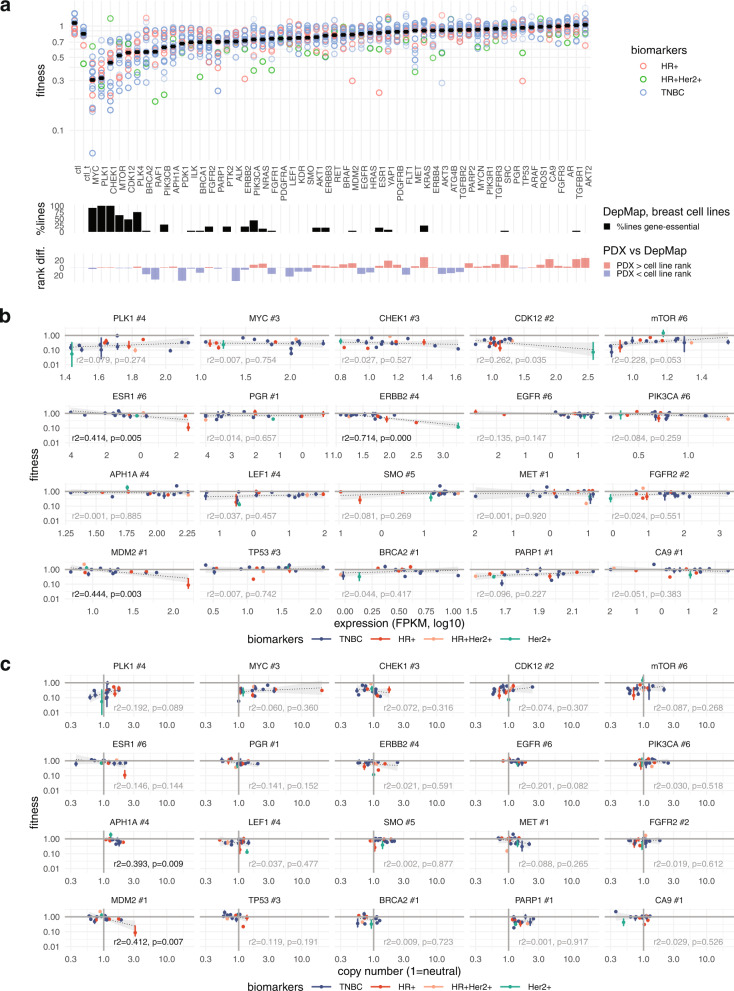
Inter-patient differences dominates the variability in fitness in PDXs. **a** Range of gene fitness for 15 PDX lines. Upper panel: circles show the median fitness for all guides targeting a gene in all experiments carried out with each PDX line (*n* = 135 PDX tumours total). Points are alpha-weighted by the standard error of the mean of fitness values for that guide/PDX line (higher standard error points more transparent). ctl and ctl_t indicate non-targeting controls and non-gene targeting controls respectively. Middle panel: percentage of Cancer DepMap breast cancer cell lines (*n* = 25) reported with in vitro CRISPR-assayed essentiality for the gene. Lower panel: difference between the gene fitness rank order in PDX lines in vivo and DepMap breast cell lines in vitro (of *n* = 56 genes). **b** Relationship between guide fitness (median, bars = 95% CI) and transcript expression, shown for 12 guides in biological replicate series from 16 PDX series (*n* = 61 PDX tumours total). Transcript expressions are from RNAseq on a tumour from the same PDX line as the fitness measurements. Statistics shown are regression r squared and the *p*-value testing for zero slope coefficient (highlighted where *p* < 0.05, two-sided significant within panel: known single-gene breast cancer biomarkers *ESR1* and *ERBB2*; also *MDM2*). **c** Relationship between guide fitness (median, bars = 95% CI) and local copy number, shown for 12 guides in biological replicate series from 16 PDX series (*n* = 61 PDX tumours total). Copy number is derived using TITAN, on WGS from a tumour from the same PDX line as the fitness measurements. Regression statistics as in **b** (highlighted where *p* < 0.05: *APH1A* and *MDM2*). Source data are provided as Source Data files.

We next assessed the variation in gene and patient fitness of core genes from PI3-kinase, integrin, DNA damage, RAS-MAPK, cell-cycle control, Notch, Wnt, Hedgehog, Hippo, hypoxia and autophagy pathways. In all series, guides targeting genes with known essential biological function (e.g. *PLK1*, *PLK4*, *CHEK1*) registered overall negative fitness (i.e. lower net growth when targeted with CRISPR), though the extent varied between PDX series (Fig. 5a, upper panel). A similar fitness pattern was observed in strong growth-promoting genes such as *MYC*, *MTOR* and *CDK12*. The fitness of these large effect size genes was concordant with those identified as broadly essential in breast cancer cell lines propagated in vitro (Fig. 5a, middle panel shows gene-sensitive proportion of 25 breast cell lines from Cancer DepMap data set^32^, Fig. S10). Among moderate effect size genes we observed differences in fitness ranks between in vivo (PDX) and in vitro (DepMap) datasets for *PDK1* (oxidative phosphorylation), *RAF1*, *CA9* (hypoxia), *ALK*, *SRC*, *LEF1* (Wnt), *YAP1* (Hippo) and *FLT1* (VEGF pathway) (Fig. 5a, lower panel). *BRCA2*, which has near neutral fitness in cell lines, exhibits a more pronounced negative fitness in primary PDXs. Taken together, these indicate functional differences between cell lines (many derived from metastatic and/or post chemotherapy treated tissues) and primary breast cancers grown in vivo.

Beyond high and moderate fitness genes, many other signaling genes were characterized by neutral fitness in many series, however with notably outlying non-neutral fitness (negative or positive) in specific series. Series-specific gene outliers included *MET* in C3278 and C1471; *PARP1* in C0331; *PIK3CA* / *PIK3CB* / *PIK3R1* in C1383 and C1471; *FGF* receptors in C1368, C3037 and C1373; and *EGFR*/*ERBB* family receptors in C1379, C1383, C1392 and C1373 (Fig. S9). Among RAS-MAPK pathway genes tested, *RAF1* sensitivity was observed in around half of PDX series. We did not observe strong fitness outliers in RAS genes (*KRAS*, *HRAS*, *NRAS*) (Figs. 5a and S9) in primary PDX, however cell lines exhibited greater median RAS pathway negative fitness overall, indicating potential constitutive differences between primary PDX and cell lines in RAS signaling. RAS genes typically gain oncogenic function through hotspot base substitution mutations, which are rare in breast cancer and absent in our PDX panel, though frequent in tumours types such as colon, lung and pancreatic ductal adenocarcinoma.

To exclude large expression or copy number effects on fitness, we next compared fitness measures with transcript expression level and genomic copy number of the targeted genes (Fig. 5b, c). These data were derived from bulk RNAseq and WGS from additional tumours in the same patient PDX series. We noted a number of genes in which fitness outliers occurred in series with highest levels of transcript expression or copy number. These included *ESR1* and *ERBB2*, which are established single gene biomarkers predictive for response to anti-estrogen or HER2-targeting therapy. Furthermore, *MDM2* fitness was most negative in series C2553, in which copy number amplification is associated with elevated expression (see section below). For the majority of genes, though, fitness did not show a clear correlation with either transcript expression or copy number. Cell signaling pathways are complex, with regulatory characteristics including functional degeneracy, thresholding, feedback and cross-pathway interaction. It is not surprising therefore that functional readouts such as gene fitness do not in general reduce to simple measures of state, highlighting the utility of making such measurements empirically.

### Validation of fitness outliers with allelic series pathway mapping and pharmacological inhibition

**Fig. 6:**
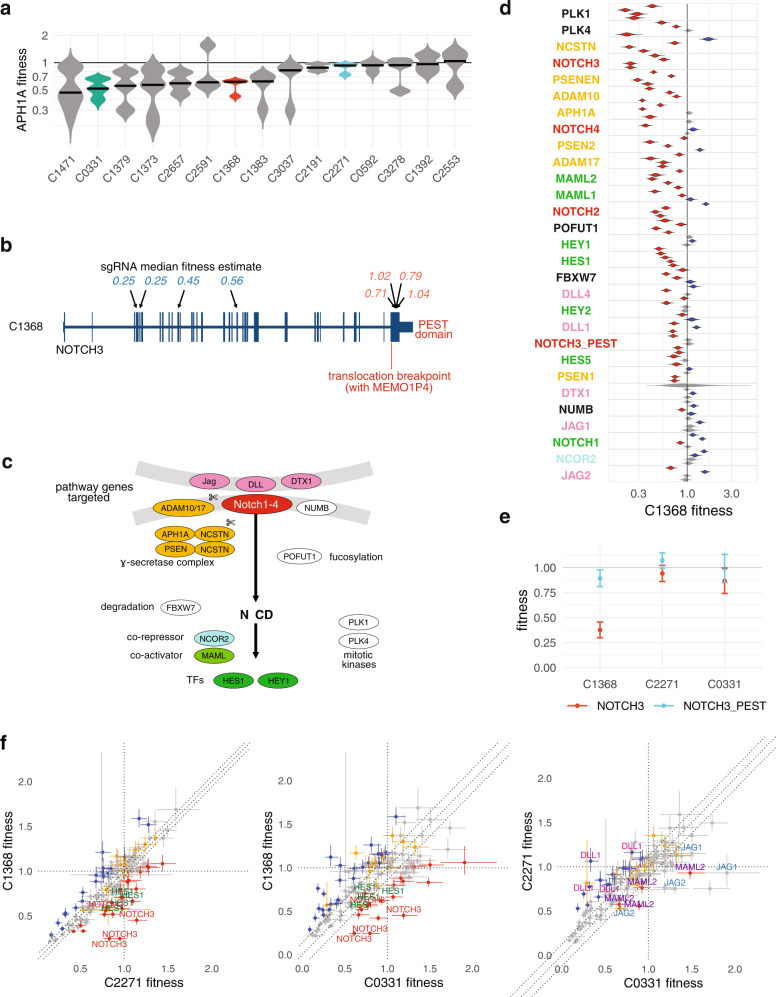
Notch pathway allelic series highlights driver mechanism in a NOTCH3-rearranged PDX line. **a** Fitness posterior distribution of guides targeting gamma secretase complex component APH1A in 15 PDX lines. PDX lines C0331, C1368 and C2271, further assayed in vivo (**d**–**f**), shown in colour. **b** Exon diagram of *NOTCH3*, showing position of translocation breakpoint with *MEMO1P4* in PDX line C1368. Arrows show position of 4 guides targeting *NOTCH3* body, and 4 guides targeting the PEST3 domain not present in the translocation, labeled with the in vivo fitness of each guide. **c** Canonical Notch signaling pathway. The genes shown are included in a Notch-focused sgRNA library, and coloured by functional group. **d** Fitness of in vivo Notch pathway and *NOTCH3* allelic series in C1368 (4 PDX tumours). Guide distributions coloured red (low fitness) and blue (high fitness) indicate estimates that differ from neutrality (fitness = 1) with false discovery rate <0.05 (Benjamini-Hochberg method). The driving function of the *NOTCH3* translocation is illustrated by reduced fitness with guides targeting *NOTCH3* body but not the missing *NOTCH3* PEST domain. Reduced fitness is also seen in multiple cleavage complex components, Notch pathway transcription factors and coactivators. **e** Fitness (mean + /− SEM) of four guides targeting 5’ *NOTCH3* regions other than the PEST domain and four guides targeting the PEST domain. C1368 shows differential sensitivity to *NOTCH3* targeting outside the PEST domain, which is missing in this PDX line due to translocation. (PDX tumours: C1368 4, C2271 4, C0331 4.) **f** Pairwise comparison of guide fitness (median, bars = 95% CI) in C1368 and two other TNBC lines, C0331 and C2271. C1368 shows lower fitness in NOTCH3 compared with both other TNBCs, as well as in the canonical transcription factor *HES1*. C2271 and C0331 show differential fitness in Notch ligands *DLL1* and *JAG1/2*, and transcriptional coactivator *MAML2*. (PDX tumours: C1368 4, C2271 4, C0331 4.) Source data are provided as Source Data files.

To illustrate the reproducibility and validation of primary in vivo fitness measures we adopted the standard approach of constructing a de-novo allelic series, in which a series of guides target multiple gene regions and also different genes within a pathway. In our initial transplant experiments, we observed several PDX lines with sensitivity to *APH1A* (Fig. 6a). This gene is a member of the gamma-secretase complex required for proteolytic cleavage of Notch receptor. We therefore selected two negative fitness (C0331, C1368) and one neutral (C2271) line for detailed fitness mapping on the Notch pathway. The Notch pathway has been implicated in breast cancer, but defining the fitness of Notch pathway genes requires phenotypic assays of cell-cell contacts in 3D or in vivo. Genome sequencing revealed that the *APH1A-*sensitive TNBC line C1368 contains a somatic translocation between *NOTCH3* and *MEMO1P4*, resulting in a *NOTCH3* transcript missing the PEST domain-containing C-terminus exon (Fig. 6b). The PEST domain of Notch normally negatively self-regulates survival through targeting the Notch receptor protein for degradation. PEST domain deletion or mutation extends the duration of Notch signaling and has been observed in several cancer types^33^. We constructed a 128-sgRNA library targeting 26 Notch pathway genes, including 4 sgRNAs targeting the *NOTCH3* PEST domain and 4 sgRNAs targeting other exons in the gene (Fig. 6b, c, Table S1). Consistent with the genomic aberration, C1368 was highly sensitive in vivo to sgRNAs targeting non-PEST 5’ regions of *NOTCH3*, whereas sgRNAs targeting the PEST3 domain were functionally neutral (Fig. 6d, e). Sensitivity to *APH1A* was mirrored in negative fitness values for multiple other members of the gamma-secretase (*NCST*, *PSEN2*, *PSENEN*) and the *ADAM10/17* cleavage complexes (Fig. 6d). Negative fitness was observed to Notch downstream signaling transcription factors HES and HEY and co-activators *MAML1* and *MAML2*, in conjunction with positive fitness in transcriptional co-repressor NCOR2 (Fig. 6d). sgRNAs targeting extra-cellular Notch ligands *DLL1/4*, *DTX1* and *JAG1/2* showed neutral fitness (Fig. 6d). These results are consistent with a dominant, cell-autonomous activation from the fusion gene. Comparison with the fitness landscapes in C2271 and C0331 confirmed the heightened relative sensitivity of C1368 to *NOTCH3*, as well as to the downstream pathway effector *HES1* (Fig. 6f, left and middle panels). In contrast the fitness landscapes of C2271 and C0331 differed mostly in extracellular signaling ligands *DLL1* and *JAG1/2*, as well as transcriptional coactivator *MAML2* (Fig. 6f, right panel). Taken together, the allelic and pathway fitness measurements reflect qualitative and quantitative differences in Notch signaling, with ligand mediated effects in wild type tumours contrasting with the PEST-domain-lacking *NOTCH3*-*MEMO1P4* translocation product, which dominantly activates pathway signaling in C1368.

**Fig. 7:**
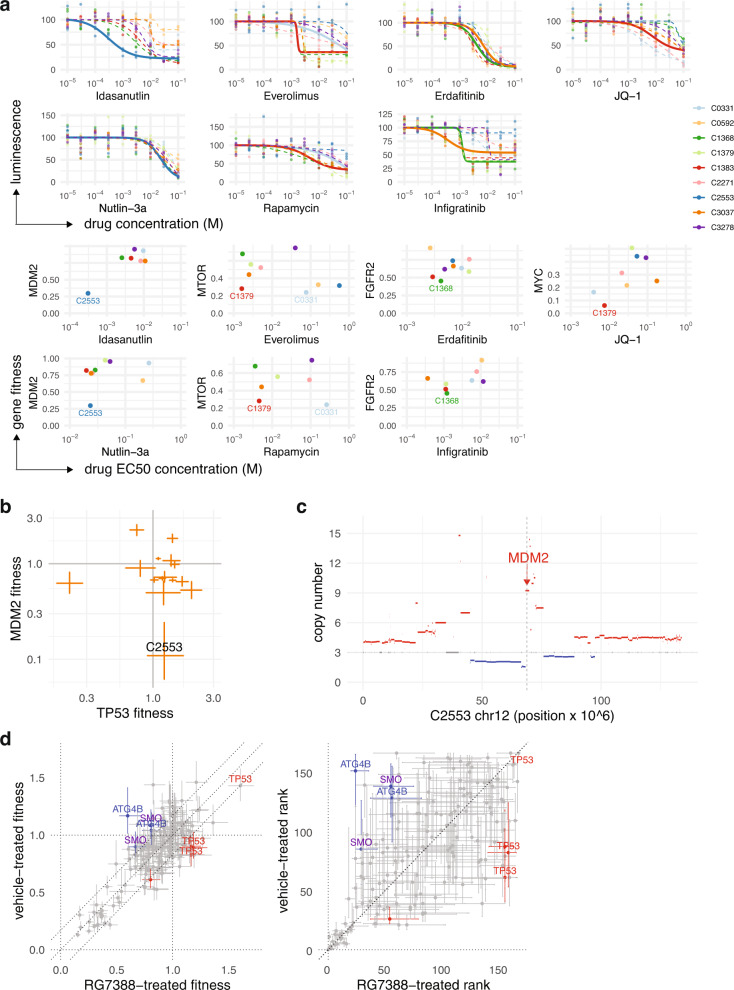
Conditional *TP53* fitness in an *MDM2*-amplified PDX line. **a** Upper panels show dose-response curves for nine PDX lines treated ex vivo with seven drugs in short-term organoid culture. Curves are shown as solid lines for PDXs that showed outlying fitness sensitivity to the drug-targeted gene(s) in the pooled in vivo CRISPR experiments. Lower panels show the median in vivo fitness of the indicated targeted gene against the half-maximal effective drug concentration (EC50). **b** Fitness measures for *TP53* (guide TP53_3) and *MDM2* (guide MDM2_1) (median, bars = 95% CI) in 17 PDX lines (60 PDX tumours total). PDX line C2553 is very sensitive to *MDM2* targeting, while close to neutral fitness for *TP53*, consistent with *MDM2* acting as a negative *TP53* regulator. **c** Chromosome 12 copy number profile for PDX line C2553, inferred by TITAN from whole genome sequence data. Copy number amplicon including *MDM2* gene is indicated. **d** Comparison of in vivo fitness and rank measurements for C2553 treated with *MDM2* inhibitor Idasanutlin or vehicle control (median, bars = 95% CI. PDX tumours: drug-treated 2, vehicle 1.) *TP53* shows a shift to positive fitness under drug treatment compared with control, consistent with a model in which pharmacological *MDM2* inhibition causes reversion to a *TP53* wild-type phenotype. Source data are provided as Source Data files.

In a second validation approach, we set out to test whether fitness phenotypes in our pooled screens are predictive of drug sensitivity in specific patient backgrounds. We thus performed short-term toxicity assays using cells dissociated from nine different PDX lines and propagated ex vivo as 3D organoids. We used a panel of seven compounds, targeting *MDM2*, *MTOR*/PI3K, *FGR* receptor signaling and *MYC* pathways. Drug-response curves were obtained over a 0.01–100 mM concentration range (Fig. 7a). Drug treatments differ considerably in biophysical mechanism of functional inhibition compared with sgRNA/CRISPR. Nevertheless, in spite of differences in time frame (96 h vs 3–10 weeks), system (in vitro culture vs in vivo xenograft) and perturbation modality (drug vs gene editing), we found several examples in which PDX line specific gene fitness outliers in the pooled CRISPR setting (Fig. S9) correspond with drug sensitivity in the organoid drug assays (Fig. 7a, PDX with fitness outliers in the drug target shown as solid dose-response curves in upper panel; labeled in comparisons with half-inhibitory drug dose in lower panel). Notable examples are *MDM2* gene fitness with sensitivity to inhibitors Nutlin-3a and Idasanutlin in PDX line C2553, *MTOR* with inhibitors Rapamycin and Everolimus in C1379, *FGFR2* with FGF receptor tyrosine kinase inhibitors Erdafitinib and Infigratinib in C1368 and *MYC* with BET inhibitor JQ-1 in C1379 (Fig. 7a).

To further demonstrate that in vivo fitness measurements have quantitative value, we probed pharmacologically an *MDM2* fitness outlier. The PDX line most sensitive to in vitro pharmacological *MDM2* inhibition (C2553) previously showed outlying fitness in *MDM2* and close to neutral fitness in *TP53* in the in vivo CRISPR screens (Fig. 7b). This *TP53* wild-type ER + PDX line contains a 6-copy amplification in *MDM2* (Fig. 7c), resulting in the highest transcript expression of this gene among our PDX series (Fig. 5b, rightmost bar in *MDM2* panel). *MDM2* is a negative regulator of *TP53*, shortening its survival by targeting for proteasomal degradation. The CRISPR and pharmacological sensitivities are consistent with attenuated *TP53* activity resulting from inhibitions by *MDM2*. We hypothesized that pharmacological *MDM2* inhibition would reactivate wildtype *TP53*, and so cause a shift from neutral to positive fitness in *TP53*. To test this, we carried out in vivo assays using the signaling library with mice randomized to receive 100 mg/kg Idasanutlin or vehicle control for 14 days, starting from a small tumour size of ~0.05 cm^3^. As predicted, all three sgRNAs targeting *TP53* exhibited a shift towards positive fitness in Idasanutlin-treated compared with vehicle-treated mice, with all three guides satisfying the multiple difference criteria (mean fitness difference 0.27) (Fig. 7d). We also noted a shift under drug treatment towards negative fitness in sgRNAs targeting autophagy-promoting gene *ATG4B* (two of three guides, mean fitness difference −0.40), consistent with reports that nutlin class drugs can upregulate the autophagy pathway^34^. Furthermore, hedgehog pathway receptor SMO showed a shift towards lower fitness on drug treatment (two of three guides, mean fitness difference −0.26), consistent with reports that this pathway can up-regulate *MDM2* expression^35^. Together these data provide additional evidence for an epistatic relationship between *TP53* and *MDM2* in primary breast cancers, and highlight that complex conditional drug-gene interactions can be dissected in PDX using the UMI-encoded fitness approach.

## Discussion

Measurements of gene fitness in replicating cells have proven to be a powerful means to understand biological functions in cancer model systems. However, understanding of the parameters that make robust quantification of fitness possible in physiologically growing tumours has been lacking. Importantly, PDXs allow functional study of an extended range of patient genotypes in many tumour types, including breast, where the study of gene fitness has been limited by the difficulty of cell line and long-term organoid generation. This is an especially acute issue for primary breast cancers, the majority of studies having been conducted with metastatic cell lines in vitro. Here we show how UMI-encoded sgRNA perturbation libraries can be used to quantify gene fitness in vivo in breast PDXs. By modeling the number and size distribution of sgRNA-UMI clones in PDX in a hierarchical Bayesian mixture framework, matching library size and length of tumour growth, reproducible estimates of gene fitness can be obtained.

Bayesian modeling of clone size distributions, in combination with winsorization of the distribution tail, has the benefit of reducing potentially confounding influences on fitness estimates of outlying large clones. This is of particular relevance to PDX models, in which the clone size distribution has a heavy-tailed form. Even in less clone-size heterogenous datasets generated from cell line culture, the use of median clone depletion measures has been shown to improve signal in CRISPR screens^18,19^. Our modeling approach could also be applied to quantify fitness in organoid cultures, in which clone size heterogeneity along with more limited biomass present a challenge to the library size that can be used quantitatively^23,36–38^. The approach is also applicable to other pooled perturbation modalities, such as screening with libraries of RNA interference constructs. In common with many CRISPR approaches which measure net proliferative output, the fitness estimates principally reflect cell-autonomous effects of gene perturbation. There may in addition be contributions from cell-cell interaction and from inter-clone competition for limiting resources, which would require additional experimental and modeling approaches to disentangle.

We find that the resolution with which gene fitness can be ascertained is critically dependent on features of the regenerative process underlying xenograft tumour growth, encoded in UMI distributions. Notably, statistical strength is increased in PDX lines in which larger numbers of transplanted cells contribute to xenografts, and in which there is lower heterogeneity in the clonal cellular contribution of these cells. Moreover, we find that these are reproducible properties of individual PDX lines. In practical terms, we can assess sgRNA panels at medium throughput (~100–200 guides in parallel), resolving in vivo gene fitness differences of moderate size (~0.2–0.5) in PDX lines which exhibit medium to high sgRNA-UMI diversity (typically, >500 UMIs per guide, SDI > 9). This level of throughput makes it feasible to carry out unbiased studies, screening several thousand genes spread over a number of libraries, to search for genes with fitness effects in a specific patient tumour genotype. In lower sgRNA-UMI diversity PDX lines, we are powered to detect fitness differences where the gene perturbation phenotype is strong (e.g. known essential genes). In all cases, resolution may be improved by pooling of biological replicates, a key feature of the Bayesian model. Importantly, in many PDX lines this allows addressing biological questions through negative or positive selection approaches. Positive selection, in which the perturbed phenotype can be orders of magnitude greater than neutral, has been successfully applied to in vivo model systems, to uncover novel tumour suppressor mechanisms^21,22,39^. In contrast, negative selection phenotypes, such as the reduced growth when a driver gene is inhibited, or conditionally lower gene fitness under drug treatment, have been more challenging to quantify at medium throughput in vivo. The UMI-informed Bayesian framework provides internal measures to assess interpretability and guide the design of suitable powered studies. At the library level, a relatively even representation of sgRNAs is desirable, to avoid wider statistical uncertainty in fitness estimates in low prevalence guides. Modeling of UMI distributions also lowers the potentially confounding impact of clones of outlying size, which may reflect developmental potential of rare cells within tumours, or chance combinatorial phenotypes in cells transduced by more than one guide. The size of gene libraries that can be screened is thus a product of line-intrinsic single cell output and transplant biomass, modified by transfection conditions and length of time in vivo. This approach would feasibly allow screens of most cellular core modules for example, by constructing libraries of ~1000 core genes^40^ and utilizing high diversity lines, with pooling from replicate transplants.

Transcriptome analysis suggests a variety of biological pathways underlie the differential clonal composition. These include elevated activity of cell cycle pathways in high sgRNA-UMI diversity lines, which may be indicative of a progenitor rather than highly differentiated developmental phenotype. In addition, interleukin and interferon cytokine pathways were elevated in low sgRNA-UMI diversity PDX lines, suggestive of a cellular bottleneck related to interaction between certain xenografted tumours and the host innate immune system (which, unlike adaptive immunity, is intact in the NRG/NSG strains used). Alternatively, elevated cytokine expression may reflect endogenous response to high rates of DNA damage, though we did not observe a correlation between sgRNA-UMI diversity and overall mutational burden, nor with genome-wide signatures of DNA damage^28^, to support this interpretation.

The quantitative assessment of gene fitness in vivo admitted detailed assessment of oncogenic driver mechanisms via allele series and drug-gene fitness. We observed outlying sensitivity to gamma secretase cleavage component *APH1A* in C1368, a line bearing a *NOTCH3*-*MEMO1P4* translocation resulting in the loss of the auto-regulatory PEST domain. Secondary screening with a Notch pathway-focused library confirmed growth dependence on the canonical Notch pathway, with this translocated Notch product rather than alternative Notch ligands as principal driver. This contrasted with two tumours that differ in ligand mediated Notch pathway signaling. We also observed outlying sensitivity to *MDM2* in C2553, a PDX line amplified at this locus. *MDM2* is a negative regulator of *TP53*, with amplification observed in ~4% of human tumours^41^. Consistent with this regulatory interaction, we showed that treatment of mice xenografted with this PDX with *MDM2* inhibitor Idasanutlin induced a shift to positive *TP53* fitness, indicating a reversion towards growth-inhibitory *TP53* function. This illustrates the utility of the approach to probe drug-gene interactions in specific tumour genotypes. Furthermore, quantification of gene fitness shifts conditional on drug treatment offers a means to screen for evolutionary double bind steering opportunities, in which phenotypic adaptation to an initial therapy results in collateral sensitivity to a second mode of intervention^42,43^.

Notably, the main source of gene fitness variation in the 181 transplant experiments described arises not from biological replicates, variations in site of transplant or differences between passages of tumour, but rather the intrinsic differences between patients. Although site transplants were largely neutral, fitness measurements of *EGFR* nevertheless exhibited a modest site-of-transplant effect, with greater negative fitness in the mammary fat pad, indicating greater dependence on *EGFR* signaling in the mammary microenviroment. Among patients, even for high negative fitness genes such as *MYC*, *PLK1*, *CDK12*, *CHEK1*, the variation in measured gene fitness can be as high as 7–10 fold between patients. The high clinical variability in responses and phenotypes in breast cancer patients is well known, and our results emphasize that intrinsic differences between patients demand the study of gene fitness in many patient tumour backgrounds to identify breast cancer sub-type fitness landscapes for any gene or pathway.

We assessed the gene and patient fitness variation of core genes from PI3-kinase, integrin, DNA damage, RAS-MAPK, cell-cycle control, Notch, Wnt, Hedgehog, Hippo, hypoxia and autophagy pathways. While the relative fitness of large effect size genes such as *MYC*, *CHEK1*, *PLK1* was conserved with that described in recent metastatic cell line CRISPR screens, some differences were observed in moderate effect size genes, notably *PDK1* (oxidative phosphorylation), *BRCA2*, *RAF1*, *CA9* (hypoxia), *ALK*, *SRC*, *LEF1* (Wnt), *YAP1* (Hippo) and *FLT1* (VEGF pathway).

Taken together, we present a method and resource for assessment of tumour gene fitness in vivo, that will be applicable to other tumour types and transplant models, enhancing the understanding of tumour biology and drug responses.

## Methods

### Biospecimen collection and ethical approval

De-identified tumour tissues from women aged 35–86 undergoing surgery or diagnostic core biopsy were collected with direct informed written consent for inclusion with University of British Columbia (UBC) Research Ethics Board (REB) under approved study protocols: REB-H16-01625 titled B-PRECISE Biobank, REB-H11-01887 titled Predictive models of drug action in breast cancer, REB-H20-00170 titled Linking clonal genomes to tumour evolution and therapeutics, Animal Care certificate REB-A07-0524, Biohazard Approval Certificate B11-0043, McGill University Health Centre (MUHC) REB -SUR99-780^44^ and accordance to Tri-Council Policy Statement: Ethical Conduct for Research Involving Humans-TCPS2. Respect for persons, concern for welfare and justice of all participants have been upheld. Participants did not receive compensation. De-identified tumour tissue is defined as biological material with direct patient identifiers removed and replaced with a code (https://ethics.research.ubc.ca/). Only privacy guardians have a level of access linking any participant’s code to their personal information and maintain the ethical duty of confidentiality. The number of patient-derived xenograft lines used in the study (*n* = 21) was chosen to ensure coverage of initiating patient tumours with a variety of histological subtypes (HR+, HR + Her2 + , Her2 + , TNBC), site (breast or metastatic) and including treatment-naive and previously therapy-treated at the time of biopsy. Only tumours that successfully engrafted in mice were used, which represents some bias in favour of more aggressive tumours within each histological subtype.

### Tissue processing

Primary surgical and core biopsy tissue samples were transported from the operating room on ice in cold 1:1 v/v Dulbecco’s Modified Eagle Medium/Ham’s F12 Medium (DMEM-F12, Corning, NY, USA). Fat was trimmed away using scalpels. A small piece of tumour tissue was removed using scalpels and fixed in 10% formalin buffered saline (Fisher Scientific, Kalamazoo, MI, USA) for histological analysis. Additional small fragments from different portions of the tissue were collected together, flash frozen in liquid nitrogen and stored at −80 °C for nucleic acid extraction. The remaining tissue was minced finely with scalpels, then mechanically disaggregated for one minute using a Stomacher 80 Biomaster (Seward Limited, Worthing, UK) in 1–2 mL cold DMEM-F12. Aliquots from the resulting suspension of cells and organoids were used for xenotransplants.

For routine passaging, xenograft-bearing mice were euthanized when the size of the tumours approached 1 mL in volume, adding together the sizes of individual growths when more than one was present. The tumour material was excised aseptically, then processed as described for primary tissue. Serially transplanted aliquots represented 0.1–0.3 % of the xenograft tumour volume.

For in vivo screen endpoints, tumours grown from lenti-CRISPR-transduced PDX material were harvested at a volume of 0.1–0.2 mL. The excised tumours were weighed, then chopped with scalpels. An aliquot weighing 0.05–0.06 g was flash frozen in liquid nitrogen or on dry ice, then stored at −80 °C to be used for nucleic acid extraction. The remaining tissue was frozen in a 47:47:6 v/v mixture of Dulbecco’s Modified Eagle Medium (DMEM, Corning): fetal calf serum (FCS, Sigma, St Louis, MO, USA): dimethyl sulfoxide (DMSO, Sigma).

### Xenografting

#### Animals

Female immuno-compromised, NOD/SCID/IL2r^*−/−*^ (NSG) and NOD/Rag1^*−/−*^Il2r^*−/−*^ (NRG) mice were bred and housed at the Animal Resource Centre at the British Columbia Cancer Research Centre. Housing was maintained in a 18–25 °C temperature range and 20–70% humidity range, with a 12 hour daylight cycle (on at 6:00am, off at 6:00 pm). Surgery was carried out on mice between the ages of 5–12 weeks. All experimental procedures were approved by the University of British Columbia Animal Care Committee (protocol numbers A15-0248 and A19-0298).

#### Subcutaneous transplants

Disaggregated cells and organoids were resuspended in 100–200 mL of a 1:1 v/v mixture of ice-cold DMEM: Matrigel (BD Biosciences, San Jose, CA, USA) and kept on ice until transplantation. Mice were lightly anesthetized with isoflurane, then the cell/organoid suspension was injected under the skin on the flank using a pre-cooled 1 mL syringe and 21 gauge needle.

#### Mammary fat pad transplants

Mice were anesthetized using isoflurane, and administered Meloxicam non-steroidal anti-inflammatory drug (5 mg/kg). The skin in the flank close to one of the #4 inguinal fat pads was shaved, cleaned with 70% isopropyl alcohol and Hibitaine soap, and a line block of 0.25% bupivacaine hydrochloride (Marcaine, AstraZeneca, Cambridge, UK) applied. The skin was opened by making a 3–5 mm skin incision in the blocked area. The fat pad was eased from the skin using blunt dissection, then exteriorized with forceps. Innocula of organoids suspended in 60–70 µL of a 50:40:10 v/v mixture of cold Matrigel: DMEM: trypan blue (Sigma) were injected into the fat pads using a pre-cooled 1 mL syringe and 21 gauge needle. The blue dye in the medium was used for visual verification that the injected bolus was contained within the fat. The fat pad was returned and the incision closed with 1 or 2 absorbable sutures.

#### Propagation of CRISPR-transduced tissue

Following transplantation, mice were monitored regularly for the appearance of palpable tumours. Once palpable, tumours were measured three times per week using calipers, and the size estimated using the formula 0.52 × *length* × *width*^2^. The animals were euthanized when tumours reached target size of 0.1– 0.2 mL. The institutionally-approved humane endpoint tumour size of 1 mL was not exceeded in any experiment. The tumours were excised aseptically, and transferred to Falcon tubes containing 5 mL ice-cold Hanks Balanced Salt Solution (Corning) supplemented with 2% FCS (HF) before tissue processing.

#### Generation and use of dual sgRNA-barcode lenti-CRISPR viral vector

##### Design of barcode and sgRNA pools

Barcode design used a partially degenerate DNA sequence NNATCNNGATSSAAANNGGTNNAACNN, where N = A/C/T/G, S = C/G, randomly incorporated during oligo synthesis (*>*4 × 10^6^ permutations)^24^. Forward (NNATCNNGATSSAAANNGGTNNAACNN) and reverse (NNGTTNNACCNNTTTSSATCNNGATNN) barcode sequence oligos pools flanked by EcoRI and NheI restriction sequences were ordered from Integrated DNA Technologies (IDT, Coralville, IA, USA), and mixed together. A second pair of forward and reverse barcode sequence oligo pools flanked by PacI and KpnI restriction sequences was ordered (IDT) and mixed together.

20 nucleotide sgRNA sequences were designed using the CHOPCHOP web tool (version 3, https://chopchop.cbu.uib.no/)^45^ specifying the PAM motif NGG, or selected from the published Avana and Brunello libraries^12,46^. Three different sgRNAs were chosen per targeted gene, based on predicted efficiency score^46^, and where possible targeting exons present in all common splice variants. Preference was also given to sgRNAs with 40-70% G-C content, and those previously identified as generating hits in cell lines screens (genomeCRISPR.org). sgRNA libraries also included a set of 20–25 non-targeting controls; either “non-targeting”, with no matching sequences in the human genome (from previously published lists^13^), or “non-gene targeting”, with matching sequences in presumed non-coding regions at least 5k base pairs distant from the nearest gene encoding region. The sgRNA sequences were obtained as complementary forward and reverse oligos from Integrated DNA Technologies, flanked by overhangs for cloning (forward: CACCG preceding sgRNA sequence; reverse: AAAC preceding and C following complementary sgRNA sequence). Individual forward and reverse oligos were mixed, then pooled as libraries for cloning.

##### Generation of dual barcode-sgRNA lentiCRISPR vector

The lentiCRISPRv2 plasmid (Addgene plasmid # 52961, a gift from Dr Feng Zhang) was initially modified by replacing the puromycin resistance gene with a dsRed-Express2 reporter gene, to enable integration detection by flow cytometry. Barcode and sgRNA pools were then incorporated in successive steps.

To facilitate efficient barcode insertion, we inserted a 60-bp random sequence filler between a pair of restriction sites either closely upstream (PacI/KpnI) or downstream (EcoRI/NheI) of the sgRNA locus in the plasmid. The plasmid was digested with one of the restriction enzymes (PacI or EcoRI), run through an agarose gel and gel purified using Monarch Gel Extraction kit (New England Biolabs, Ipswich, MA, USA) to isolate the digested plasmid backbone. The filler sequence was ligated into the plasmid with T4 ligase. The plasmid was amplified by heat-shock transformation into Stbl3 bacteria, and transformants cultured on Luria-Bertani-ampicillin-agar (LB-amp-agar) solid medium overnight. A single colony was picked and further cultured with LB-amp liquid medium, and plasmid extracted using Monarch Miniprep Kit (New England Biolabs). Presence of the filler was verified with Sanger sequencing. For barcode pool insertion, filler-containing lentiCRISPRv2 plasmids were digested with a restriction enzyme pair (PacI/KpnI or EcoRI/NheI), gel purified, and ligated with a pool of generate dsDNA oligonucleotides containing the barcode pool. Barcoded plasmids were electroporated into ElectroMAX DH10B T1R bacteria according to the manufacturer’s instructions and cultured overnight in 200 ml LB-amp. The barcode-containing plasmid was extracted using PureLink HiPure Plasmid Filter Maxiprep Kit (Thermo Fisher Scientific, Waltham, MA, USA), and frozen for future use as recipient for sgRNA pools.

Pooled sgRNA oligonucleotides were incorporated into BsmBI/Esp3I (Thermo Fisher Scientific) digested lentiCRISPRv2 plasmids at 3:1 insert-to-vector molar ratio with Quick Ligase (New England Bio labs). Ligated vectors were purified with NEB Monarch PCR & DNA Cleanup Kit (New England Biolabs) and subsequently transformed into electrocompetent ElectroMAX DH10B T1R E. coli (Thermo Fisher Scientific) using a 1 mm cuvette in a BioRad GenePulser Xcell System at 1800V, 25 µF and 200 ohms. Electroporated cells were incubated overnight in 200 mL of BD Difco LB broth Miller (Thermo Fisher Scientific) with ampicillin at 37 °C. Overnight culture was collected by spinning in a Beckman Coulter Avanti JXN-26 high-speed centrifuge at 6000 rpm for 10 min and the plasmids were extracted using a PureLink HiPure Plasmid Filter Maxiprep Kit (Thermo Fisher Scientific). Purified vectors were validated by targeted sequencing of guide inserts on Illumina MiSeq 2 × 150bp runs.

Sequence diversity of sgRNA and barcodes was validated by targeted sequencing of a PCR amplicon containing both sgRNA and barcode (as described below in Sequencing Library Preparation). This showed 90th/10th centile guide count ratio of 3.94, 5.27 and 3.01 for libraries Signaling1, Signaling2 and Notch respectively, which are in the expected range for pooled cloning.

Testing for any impact of UMI incorporation on efficacy of CRISPR-mediated knockdown was carried out by cloning an sgRNA targeting eGFP into the lentiCRISPRv2 vector. Then eGFP-expressing HEK293T cells (a clonal line derived from parental HEK293T cells) were transduced with plasmid vector using TransIT-Lenti Transfection Reagent (Mirus Bio, Madison WI, USA) following manufacturer’s instructions. After 8 days’ culture, cells were harvested with trypsin/EDTA and incubated with propidium iodide (Sigma) for identification of viable cells. The knockdown proportion (eGFP low) was determined by flow cytometry (LSRFortessa, BD Biosciences, San Jose, CA, USA) using FlowJo software version 10.7.1 (Beckton Dickinson).

##### Lentiviral packaging and titration

HEK293T packaging cells (ATCC) were transfected using TransIT-Lenti Transfection Reagent following manufacturer’s instructions. Briefly, 3 plasmids containing sgRNA-barcoded lentiviral vector (20 µg), PAX2 (15 µg) and VSV-G (10 µg) were dissolved in 4.5 mL Opti-MEM Reduced Serum Medium (Thermo Fisher Scientific), and 135 µL transfection reagent added. After 30 minutes the mixture was added dropwise to HEK293T cells at 70–90% confluent culture in a 150 mm dish. Cells were cultured in DMEM supplemented with 10% FCS and 10 mM Hepes (STEMCELL Technologies), with a change to fresh medium the following morning. Virus-containing supernatant was harvested four times, between 24 and 72 h following transduction. Harvests were pooled and pelleted by ultracentrifugation (Beckman Coulter Optima XE-90, spun at 107,000 × g for 90 min), and resuspended in DMEM (~1.2 mL final volume per 6 × 150 mm plates’ culture). Functional viral titre was determined by transducing a limiting dilution series of viral doses into 184hTERT-L9 cells (a clonal line derived from parental 184hTERT cells, a gift from Martha Stampfer, University of Berkeley, CA, USA). Cells were harvested 3 days following transduction, and the proportion of dsRed-expressing cells determined by flow cytometry (LSRFortessa) using FloJo software version 10.7.1. Titre was calculated using Poisson statistics for those dilutions that resulted in 5–25% dsRed+ cells. Typical functional titres of 0.5–3 × 10^8^ infectious units per ml were obtained, comparable with non-UMI-containing vectors.

##### Ex-vivo lentiviral transduction of PDX-derived cells

Mice were euthanized, then PDX tumours were removed aseptically. The tissue was minced with scalpels and mechanically disaggregated with a Seward Stomacher 80 paddle blender (Seward, Worthing, UK) for 1 minute in cold DMEM. A suspension of small organoids and cells was separated from larger tissue fragments by drawing into a P1000 pipette tip, then transferred to an Eppendorf tube. After centrifugation (5 minutes at 1200 rpm), the size of the pellet was estimated visually from the Eppendorf tube volume scale. The material was then divided into aliquots of suitable size for transplantation into individual mice (each ~0.025–0.030 cm^3^ pellet volume, typically 3–6 aliquots per originating 1 cm^3^ excised tumour volume). Red blood cell lysis was performed by incubating in ice-cold 0.8% w/v ammonium chloride solution (STEMCELL Technologies) for 10 minutes and washing with HF. Each aliquot was resuspended in 0.25 ml pre-warmed DMEM supplemented with 5% FCS and lentivirus at the desired dose (typically, 7–10 × 10^6^ infectious units, Table S2). Aliquots were transferred to individual wells of a pre-warmed 24-well non-tissue-culture treated plate and incubated at 37 °C for 4 hours. Following incubation, well contents were transferred to Eppendorf tubes and residual virus removed by washing 3 times with cold HF. Aliquots intended for subcutaneous transplantation were resuspended in 150 µL ice-cold 1:1 v/v mixture of Matrigel: DMEM. Aliquots intended for mammary fat transplantation were resuspended in 65 µL ice-cold 0.45: 0.45: 0.1 v/v mixture of matrigel: DMEM: trypan blue. The aliquots were kept on ice for up to 2 hours awaiting transplantation into mice.

Transduction efficiency was estimated by flow cytometry following in vitro culture. Wells of a 24-well plate were coated by incubating at 37 °C for 1 hour with a 1:60 dilution of collagen I (Sigma) in PBS. Aliquots of virally-transduced tissue were added to coated wells in 1 mL DMEM supplemented with 5% FCS. After 3 days, single cell suspensions were prepared by incubating in trypsin/EDTA (Sigma), then dispase (STEMCELL Technologies) supplemented with DNaseI (Sigma), then passing through a 40 micron filter. Cell pellets were resuspended in ice-cold HF supplemented with 4‘,6-diamidino-2-phenylindole (DAPI, Sigma). Transduced cell proportions were determined by flow cytometry (LSRFortessa) as the fraction of dsRed+ cells within a viable (DAPI-) gate.

### Drug assays

#### Ex vivo cytotoxicity assays in organoid culture

Freshly harvested PDX tissue was dissociated to single cells using a gentleMACS dissociator with human dissociation kit (Miltenyi Biotec, Germany) and incubated at 37 °C with constant agitation. Red blood cells were lysed using ice-cold 0.8% w/v ammonium chloride solution (STEMCELL Technologies). Cells were filtered through a 70 micron filter before being resuspended in 1:1 mix of Matrigel: breast cancer organoid media (composition as specified in^47^). 3000 cells per 20 µL were seeded into each well in a white 384 well plate (Corning) using an Integra Assist Plus automated pipettor. Plates were incubated at 37 °C for 15 minutes to allow Matrigel to solidify before an additional 10 µL breast cancer organoid culture medium was added to each well. 60 µL PBS was added to surrounding wells to minimize evaporation artifacts. Organoids were allowed to form for 3 days, after which 10 µL of 3× concentrated drug diluted in culture medium to account for the cell: Matrigel mix. Organoids were incubated with drugs for 96 hours before being lysed, and viability measured using CellTitreGlo 3D Viability Assay (Promega, Madison, WI, USA) following manufacturer’s instructions, with luminescence measured by microplate reader (SpectraMax 3, Molecular Devices, Sunnyvale, CA, USA). Drug response curves and EC50 concentrations were fitted with the R tidydrc package (a wrapper for the drc package), using four parameter log-logistic function LL.4 (https://cran.r-project.org/web/packages/drc/drc). Organoid PDX-drug assay data were filtered to remove a minority that failed to fit with the LL.4 model (1/63 assays), fit with positive slope (fit parameter b < 0, 1/63 assays), or where the dose-response fit covered a lower than 20% range over the drug doses tested (4/63 assays). Drugs used were Idasanutlin (Medkoo Biosciences, Morrisville, NC, USA), Nutlin-3a (MedChemExpress, Monmouth Junction, NJ, USA), Infigratinib (MedChemExpress), Erdafitinib (MedChemExpress), JQ-1 (MedChemExpress), Rapamycin (Selleck Chemicals, Houston, TX, USA) and Everolimus (Selleck Chemicals).

#### In vivo drug administration

Vehicle was prepared by dissolving 20 mg/ml Klucel hydroxypropyl cellulose average Mw 100,000 (Sigma), 1 mg/ml Tween-80 (Sigma), 0.9 mg/ml methylparaben (Sigma) and 0.1 mg/ml propylparaben (Sigma) in sterile water, and kept refrigerated. Mice bearing replicate PDX tumours were randomized into control and drug-treated groups when the average tumour size was ~0.05 ml. For 14 consecutive days mice received a dose by oral gavage either of vehicle alone (control group) or 100 mg/kg Idasanutlin (MedKoo Biosciences) suspended in vehicle (drug-treated group). Mice were euthanized on the day of the final drug dose, four hours after drug dosing, and tumours were harvested for processing.

#### CRISPR screen sequence generation

##### Sequencing library preparation

Flash frozen xenograft tumour samples were defrosted. Genomic DNA was extracted using the QIAAmp DNA Mini kit (Qiagen), following manufacturer’s protocol for extraction from tissues, with the exception that double the volume of reagents was used prior to loading onto DNA columns (buffers AL and ATL, proteinase K, ethanol). DNA yield was quantified using QUBIT fluorometer and dsDNA BR Assay Kit (Thermo Fisher Scientific). Aliquots containing 8–10 mg DNA were set aside for sequencing library preparation.

Nextera adaptor sequences were appended to the 5’ end of the sgRNA region primers with the addition of single nucleotides to allow downstream barcoded adaptor attachment by PCR. Multiplex PCR was performed using a T100 Thermal Cycler and Platinum Multiplex PCR Master Mix (Bio-Rad Laboratories, Hercules, CA, USA). Enzymatic cleanup of the PCR product was carried out using ExoSAP-IT PCR Product Cleanup Reagent (Thermo Fisher Scientific). Barcode PCR was completed using T100 Thermal Cycler, Nextera XT Index Kit v2 (Bio-Rad Laboratories), and FastStart High Fidelity PCR System (Sigma). The PCR products (approximately 400 bp, including adaptors) were size selected and cleaned using SPRIselect beads (Beckman Coulter) with a 1:1.2 library:beads ratio. The DNA size was analyzed using a High Sensitivity DNA Kit (Agilent Technologies, Santa Clara, CA, USA) on an 2100 Bioanalyzer (Agilent Technologies) and the DNA was quantified using dsDNA BR Assay Kit (Invitrogen, Carslbad, CA, USA) on a Qubit 4 Fluorometer (Invitrogen). Samples were normalized and pooled to create a library and each library was denatured to 2 nM or 4 nM. Sequencing runs pooling up to 12 sample libraries were carried out on a MiSeq benchtop sequencer (Illumina, San Diego, CA, USA, running Illumina Control Software, currently version 4.0.0.1769) according to manufacturer’s protocol for 2 × 151 bp pair-end runs using MiSeq Reagent Kits v2 300 Cycle (Illumina). Sequencing runs pooling 13–47 samples libraries were carried out on a NextSeq sequencer (Illumina) according to manufacturer’s protocol for 2 × 151 bp pair-end runs using NextSeq 500 System Mid-Output Kit 300 Cycle (Illumina).

##### Extraction of sgRNA and UMI sequences

FASTQ files were first filtered to remove paired reads for which either forward or reverse reads had a lower average phred quality score than 30. The forward read, containing the sgRNA sequence, was trimmed to a substring from 24 to 43 nucleotides downstream from invariant vector sequence TCTTGTGGAAAGGACGAAACACCG. sgRNAs were identified within the substring by exact string matching against the set of known 20 nucleotides targeting sequences in the library used, or failing that a string match with no more than 1 nucleotide mismatch. The reverse read, containing the UMI sequence, was trimmed to a 50 nucleotides substring down stream of invariant vector sequence AGCCTCACTGGCCGTCGTTTTACA. UMI sequences were identified within the substring by exact string matching to the sequence NNGTTNNACCNNTTTSSATCNNGATNN, complementary to the UMI reference, or failing that a string match with no more than 1 nucleotide mismatch. Stringency of FASTQ filtering was relaxed to allow for up to 3 nucleotides mismatches for model selection analysis. To standardize dataset depth, and reduce the confounding effect of duplicate reads in more deeply sequenced datasets, extracted sequences were downsampled to a maximum of 2 × 10^6^ sgRNA-UMI reads. Reads with the same combination of sgRNA and UMI sequence were considered a separate clone, and the numbers of downsized reads matching that sgRNA-UMI sequence the clone size.

Previous studies have documented deviations in UMI sequences from the sequences originally introduced by transduction, presumed to arise through errors in replication, PCR or optical sequence reading^25^. To adjust for this, we carried out merging of clones with closely similar sgRNA-UMI sequence, using the UMI-Tools Directional algorithm^25^. For each sgRNA in turn, UMI sequence clones are represented as a network of nodes, with edges drawn connecting pairs of nodes where: (i) UMI sequences differ at exactly 1 position, and (ii) *n*_*a*_ > 2*n*_*b*_ − 1, where *n*_*a*_ and *n*_*b*_ are the larger and smaller clone sizes for the pair. Any cluster of interconnected nodes, in which each node can be connected to any other by series of uninterrupted edges, is then merged into a single clone. The merged clone has size equal to the sum of constituent node clone sizes, and is assigned the UMI of the constituent node with greatest clone size. Comparison of downstream fitness estimates made with or without pre-processing with UMI-Tools indicated that clone merging has a very small effect on median fitness estimates but results in somewhat wider dispersions consistent with the implied reduction in number of independent datapoints modeled.

### Whole genome and transcriptome sequence data analysis

#### Sequence data generation

For each patient PDX line in the study, tissue aliquots were collected from (i) a sample PDX tumour; (ii) the patient tumour that initiated the PDX line; and (iii) normal tissue (saliva or blood buffy coat) from the patient. Genomic DNA was extracted using the QiaAmp or Allprep kits (Qiagen). Library construction and sequencing was carried out using Illumina HiSeq2500 whole genome shotgun v4 chemistry with paired end 125 bp reads, targeting an average genome-wide coverage of 80 for PDX and patient samples and 40 for normal samples.

RNA was extracted from a tissue aliquot from a sample PDX tumour (from the same PDX line but not necessarily the same tumour as used for the PDX DNA aliquot), using the Allprep kit (Qiagen). ssRNAseq libraries were prepared and sequenced on a HiSeq2500 sequencer, pooling 3 samples per sequencing lane.

#### Alignment

Reads were aligned to the hg19 reference genome using bwa mem v0.7.6a. Duplicates were marked with picard MarkDuplicates v2.18.14 (http://broadinstitute.github.io/picard/). RNAseq libraries were aligned using the STAR pipeline^48^ version 2.4.2a (https://github.com/alexdobin/STAR).

#### Bioinformatic removal of mouse reads

Whole genome and whole transcriptome sequence libraries from PDXs were depleted for putative contaminating mouse reads using species filter Biobloom^49^, retaining reads that were unambiguously human-classified. To avoid false-positive downstream mutation calls when comparing with other library types, tumour and normal libraries WGS were also processed with this filter. Human-classified reads were realigned to the hg19 reference genome using bwa mem^50^ for downstream variant calling.

#### Single nucleotide variant and indel calling

Somatic indels were identified using Strelka v1.0.14^51^, and both germline and somatic SNVs were called using MutationSeq v4.2.0^52^. Both algorithms were applied using default parameters. GENCODE release 19 was used to annotate variants with gene name, while the predicted effects of SNVs were annotated using SnpEff 4.0e. Additionally variants were annotated with ClinVar (https://www.ncbi.nlm.nih.gov/clinvar)^53^ and COSMIC (https://cancer.sanger.ac.uk/cosmic)^54^ variant database entries having exact sequence matches. A subset of predicted high impact somatic variants was selected as those with (i) mutationSeq probability > 0.85, and (ii) variant allele frequency > 0.3, and (iii) predicted as high impact by at least one of SnpEff (any of the terms: stop_gained, splice_donor_variant, splice_acceptor_variant, missense_variant, stop_lost) or ClinVar (any of the terms: pathogenic, likely pathogenic). For BRCA1 and BRCA2 genes, predicted high impact variants in the germline were also included.

#### Breakpoint calling

Breakpoint prediction was conducted using deStruct^55^ version 0.4.3 (https://github.com/amcpherson/destruct/) to generate per cell breakpoint counts. Breakpoints were filtered for a minimum of 5 split reads, and with predicted sequences anchored by at least 250 nucleotides on either side of the predicted breakpoint.

#### Copy number calling

TITAN version 1.0.10^56^ was run on WGS data to infer logR copy number values. 10 separate TITAN runs were made with parameters 1, 2, 3, 4 or 5 subclonal clusters and initializing ploidy of 2 or 4. The best fitting run was selected for each dataset, based on lowest *S*_*Dbw* Validity Index score.

#### Single cell sequencing with DLP+

The sample genome-wide single cell copy number profile displayed for PDX series C2553 was prepared with the Direct Library Preparation (DLP+) protocol as described in^31^.

#### Mutation signature analysis

We derived mutation signature probabilities using the MMCTM method, given signatures previously inferred from a set of high grade serous ovarian carcinomas and triple negative breast cancers^28,57^. The PDX sample datasets were clustered together with the other datasets to form clusters defined solely by signature activity using hierarchical clustering with Euclidean distance and Ward’s linkage method.

#### Inference of mutational clonal clusters

We inferred the number of genomic clonal clusters in PDXs by applying population structure model PyClone-VI (https://github.com/Roth-Lab/pyclone-vi)^26^ to mutation data derived from bulk WGS. Model inputs for single nucleotide variants were from MutationSeq v4.2.0^52^. Model inputs for major and minor allele copy number were from TITAN version 1.0.10^56^, using the best fitting run based on *S*_*Dbw* Validity Index score.

#### Differential pathway analysis

To identify pathways differentially active in high vs low UMI diversity PDX lines, we first ordered PDX lines based on the average SDI per million reads of tumours generated from those lines. We selected a group of 6 lines with lowest diversity (C3466, C2438, C1557, C1375, C1373, C0468) and a group of 6 lines with highest diversity (C3278, C1368, C0331, C2271, C2191 and C3037). We next generated a set of genes differentially expressed between the two groups. For this, raw RNAseq read counts were TMM-normalized using edgeR (https://bioconductor.org/packages/release/bioc/html/edgeR.html). Limma-voom (https://bioconductor.org/packages/release/bioc/html/limma.html) was used to adjust for heteroskedasticity in count data, and generate log2-fold gene expression differences and *p*-values by linear modelling. A subset of genes with *p*-value no greater than 0.1 was passed to GSEA^27^ for differential pathway analysis, using the Reactome 2016 Hallmark set of curated pathways (https://reactome.org/), and a false discovery rate of 0.05.

### Reporting summary

Further information on research design is available in the Nature Research Reporting Summary linked to this article.

## Supplementary information

Supplementary Information

Description to Additional Supplementary Information

Supplementary Data

Reporting Summary

## Data Availability

Raw data from targeted sequencing, whole genome sequencing and RNAseq have been deposited in the NCBI Sequence Read Archive (SRA) under BioProject ID PRJNA842677. Derived UMI-sgRNA count files have been deposited at Zenodo 10.5281/zenodo.6584802. Mutation annotation was carried out using ClinVar (https://www.ncbi.nlm.nih.gov/clinvar) and COSMIC (https://cancer.sanger.ac.uk/cosmic). Source data are provided with this paper.

## Source data

Source data

## Code availability

Fitness modeling used the Blang probabilistic programming language. The model code and sample datasets are available on Github (https://github.com/aroth85/humi_pipeline) (10.5281/zenodo.6596290)^58^. Blang code is available at https://github.com/UBC-Stat-ML/nowellpack (10.5281/zenodo.6595131)^59^. Pre- and post-processing of results used custom scripts in Python and R version 3.6.3. Mutation-calling algorithms mutationSeq, TITAN, deStruct and signature inference MMCTM are published, and cited in the text. The CHOPCHOP webtool (version 3, https://chopchop.cbu.uib.no) was used for CRISPR guide design.
