# Supplementary material for: Accurate determination of CRISPR-mediated gene fitness in transplantable tumours
Source: Nat Commun. 2022 Aug 4;13:4534. doi: 10.1038/s41467-022-31830-2 (PMC9352714; doi:10.1038/s41467-022-31830-2)
Supplement: Supplementary file 1 — Supplementary Information [file 41467_2022_31830_MOESM1_ESM.pdf]

Supplementary Data and Methods

Eirew et al.; Accurate determination of CRISPR-mediated gene fitness in transplantable tumours

Contents

Supplementary Figures **3**

Supplementary Tables **23**

Supplementary Methods: Inference of fitness informed by barcode distributions **40**

1.1 Mathematical notation 40

1.1.1 Types; targeting and non-targeting sgRNA 40

1.1.2 Final condition measurements 40

1.1.3 Initial condition measurements 42

1.1.4 Experimental replicates and conditions 42

1.2 Summary statistics 42

1.3 Definition of fitness 43

1.3.1 Choice of test function 44

1.4 Frequentist approach 44

1.4.1 Confidence intervals 46

1.4.2 Empirical validation of the calibration of the delta method confidence intervals . . 47

1.5 Bayesian approach 48

1.5.1 General structure of the models and graphical models 49

1.5.2 Clone size distribution families 50
1.5.3 Relaxed assumptions on $I_{e,t}$ 52
1.5.4 Multiple infection model 53
1.5.5 Inference 54
1.5.6 Goodness of fit 56
1.5.7 Bayesian model selection 58
1.5.8 Prior sensitivity 58
1.6 Choice of reference 61
1.7 Poset framework 61
1.8 Difference criteria in comparisons 61
1.9 Comparison with cell line essentiality dataset 63

Supplementary Figures

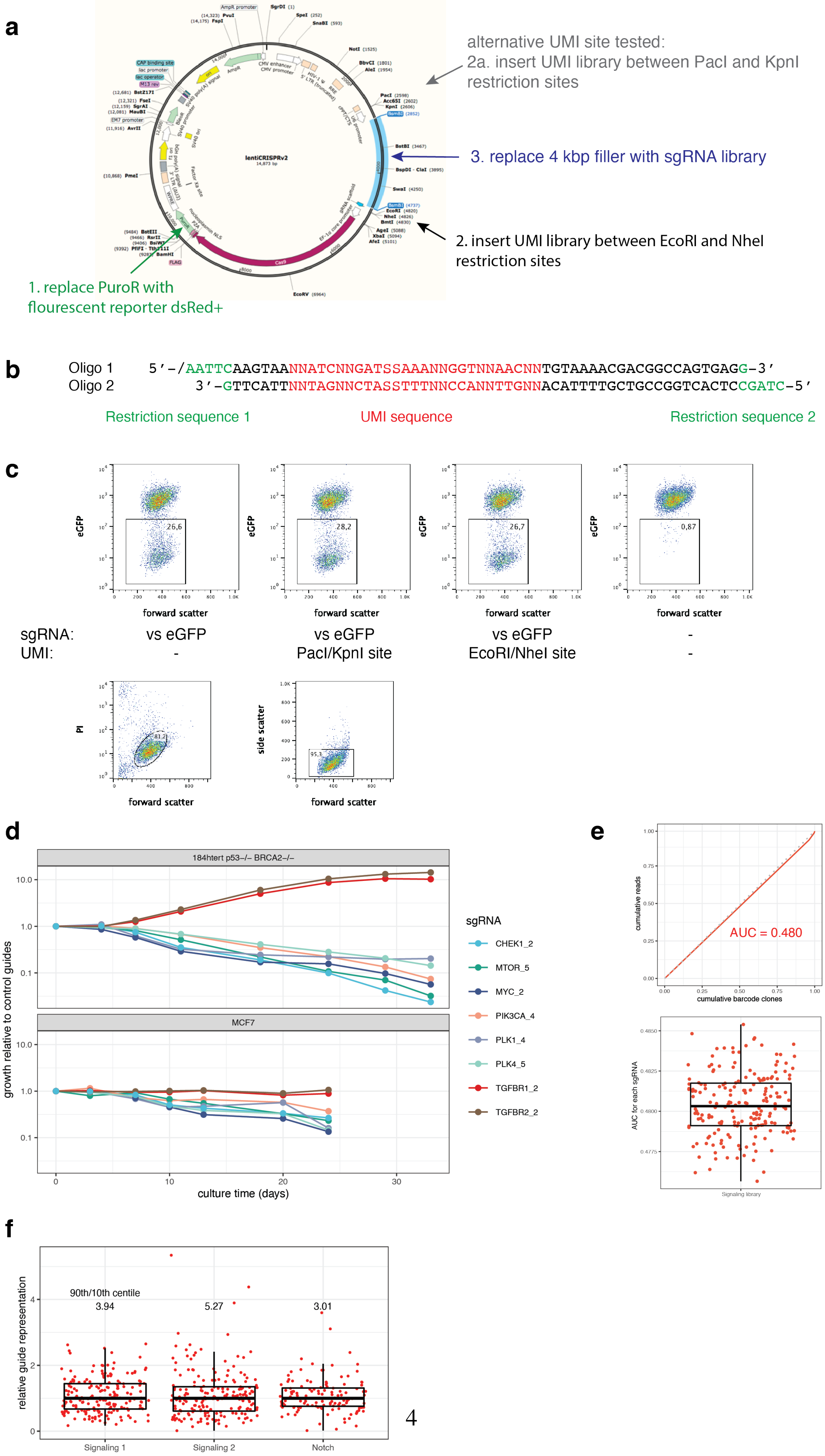
a
lentiCRISPRv2
14,873 bp
alternative UMI site tested:
2a. insert UMI library between PacI and KpnI restriction sites
3. replace 4 kbp filler with sgRNA library
2. insert UMI library between EcoRI and NheI restriction sites
1. replace PuroR with flourescent reporter dsRed+
b
Oligo 1 5'-/AATTCAAGTAANNATCNNGATSSAAANNGGTNNAACNNTGTAAAACGACGGCCAGTGAGG-3'
Oligo 2 3'-GTTCATTNNTAGNNCTASSTTTNNCCANNTTGNNACATTTTGCTGCCGGTCACTCCGATC-5'
Restriction sequence 1
UMI sequence
Restriction sequence 2
c
eGFP
forward scatter
26,6
28,2
26,7
0,87
sgRNA: vs eGFP vs eGFP vs eGFP -
UMI: - PacI/KpnI site EcoRI/NheI site -
PI
side scatter
81,2
95,3
d
184htert p53–/– BRCA2–/–
MCF7
growth relative to control guides
culture time (days)
sgRNA
CHEK1_2
MTOR_5
MYC_2
PIK3CA_4
PLK1_4
PLK4_5
TGFBR1_2
TGFBR2_2
e
cumulative reads
cumulative barcode clones
AUC = 0.480
AUC for each sgRNA
Signaling library
f
relative guide representation
90th/10th centile
3.94
5.27
3.01
Signaling 1
Signaling 2
Notch

Figure S 1 **a** Map of lentiCRISPRv2 plasmid, and sequential cloning steps to generate dual UMI-sgRNA vector. **b** Forward and reverse oligo sequences used to generate UMI library for cloning. Degenerate nucleotide positions indicated by N (C, T, A or G) and S (G or C). **c** Upper panels are FACS plots showing eGFP-expressing HEK293T cells transfected with plasmid containing an eGFP-targeting sgRNA and a UMI sequence absent or cloned into sites upstream or downstream of the sgRNA. Gene silencing efficiency is comparable with or without the UMI sequence in the plasmid. Lower panels show prior gating applied, excluding non-viable (forward scatter low or PI+) then side scatter high populations. **d** Population depletion of selected gene-targeting guides within a 192-guide pooled lenti-Cas9-sgRNA-UMI library propagated in two cell lines. The expected depletion of guides targeting known essential and growth-promoting genes (*PLK1*, *PLK4*, *MYC*, *CHEK1*, *MTOR*, *PIK3CA*) is observed. Guides targeting mediators of contact inhibition (*TGFBR1*, *TGFBR2*) are enriched in the 184hTERT-derived normal-like line. **e** Upper panel: Lorenz curve (red) of cumulative reads vs cumulative clone numbers in UMI-sgRNA plasmid library containing 192 signaling guides. Area under the curve is close to 0.5 (dotted line diagonal), indicating a high level of clone size homogeneity. Lower panel: area under Lorenz curve shown for each of the 192 guides in the library, indicating a high level of clone size homogeneity in all. Boxplot lines at 25th, 50th and 75th centiles, vertical lines extend to furthest datapoint within 1.5 interquartile range distance of box limits. **f** Relative count representation of guides in the sgRNA libraries used (median=1). Boxplot lines at 25th, 50th and 75th centiles, vertical lines extend to furthest datapoint within 1.5 interquartile range distance of box limits. Summary statistic shows the ratio of the 90th to 10th centile guide by representation.

Source data are provided as Source Data files.

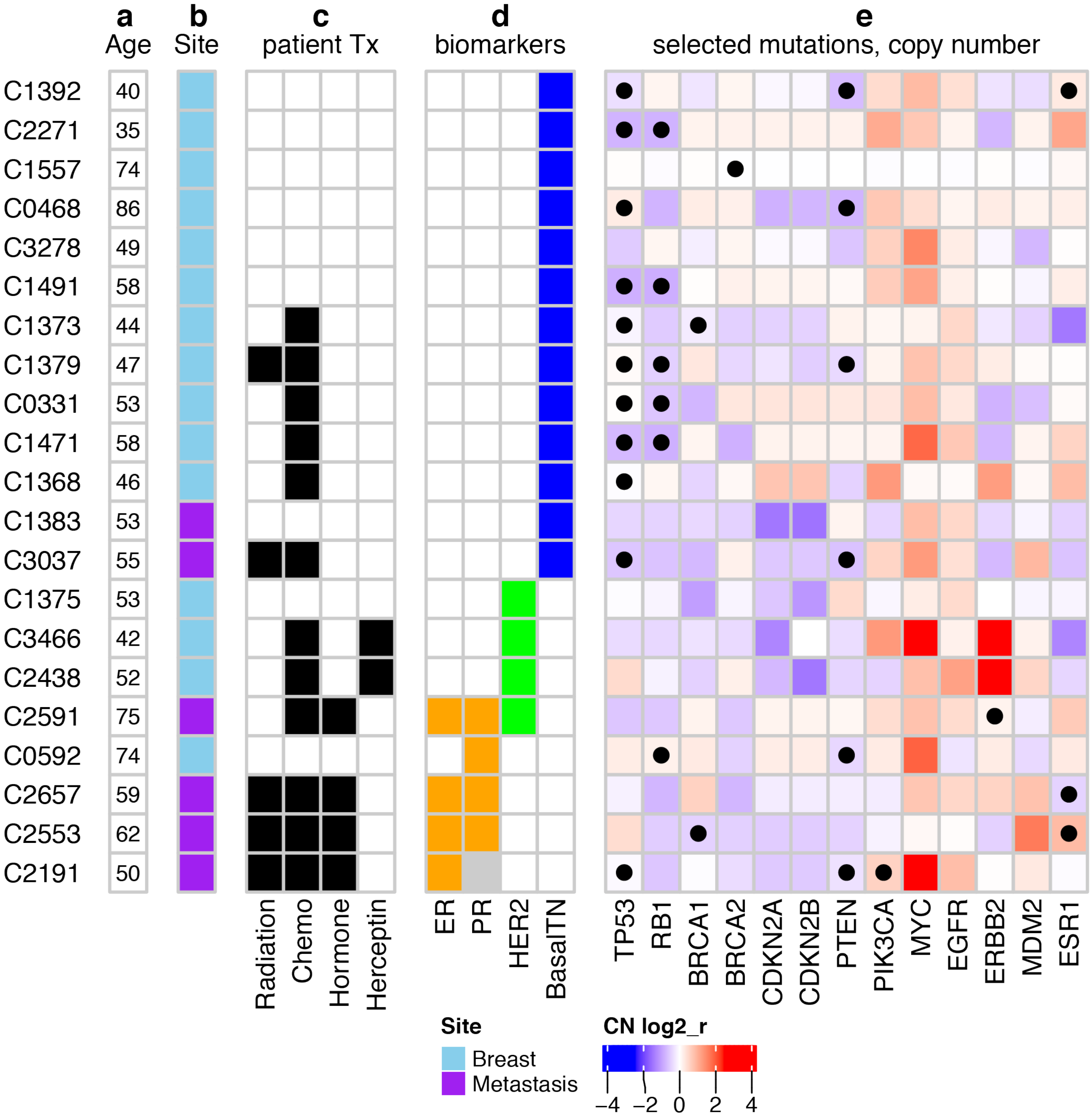

Figure S 2 Clinical and mutational summary

Figure S 2 a Patient age at time of biopsy. **b** Biopsy site. **c** Treatment modalities prior to biopsy (black). **d** Clinical biomarkers, assayed by IHC or FISH (for HER2). BasalTN indicates ER-, PR- and HER2-, and at least one of CK5/6+, EGFR+, Nestin+ or INPP4B-. **e** Heatmat displays copy number at selected gene loci, determined by TITAN (log2 ratio relative to genome-wide copy number average). Black dots indicate presence of single nucleotide variants, insertions or deletions.

Source data are provided as Source Data files.

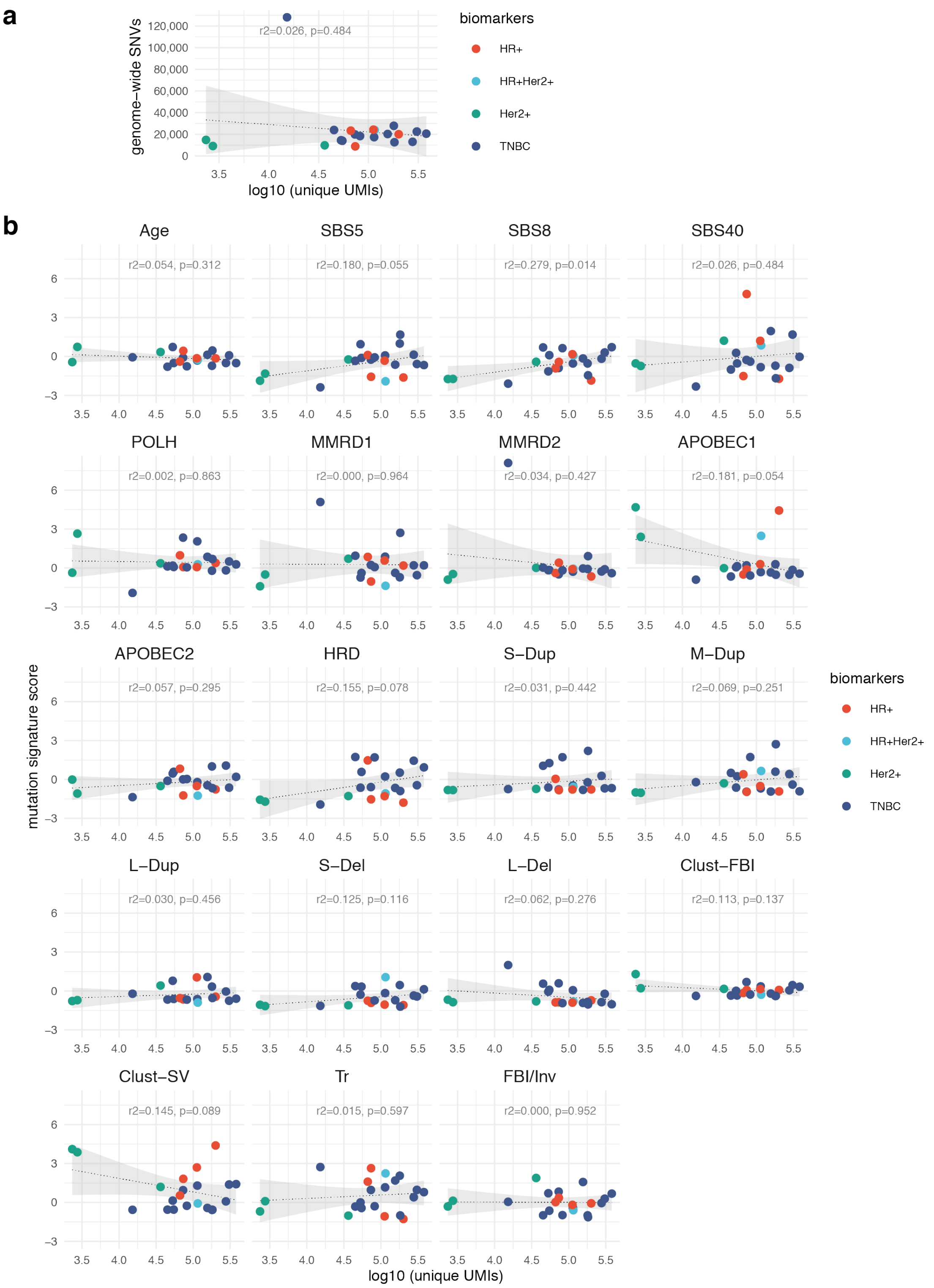

Figure S 3 UMI diversity and mutation signatures

Figure S 3 **a** Total number of genome-wide somatic SNVs (called by mutationSeq, pr>0.85) plotted against the mean UMI counts per million reads for 22 PDX series. Plot displays linear regression line, 95% confidence band, r squared statistic and the p-value testing for slope coefficient=0 (highlighted where $p<0.05$). **b** Panels show the standardized relative probability of 19 mutation signatures in PDX lines against the mean UMI counts per million reads. Plots display linear regression line, 95% confidence band, r squared statistic and the p-value testing for slope coefficient=0 (highlighted where $p<0.05$).

Source data are provided as Source Data files.

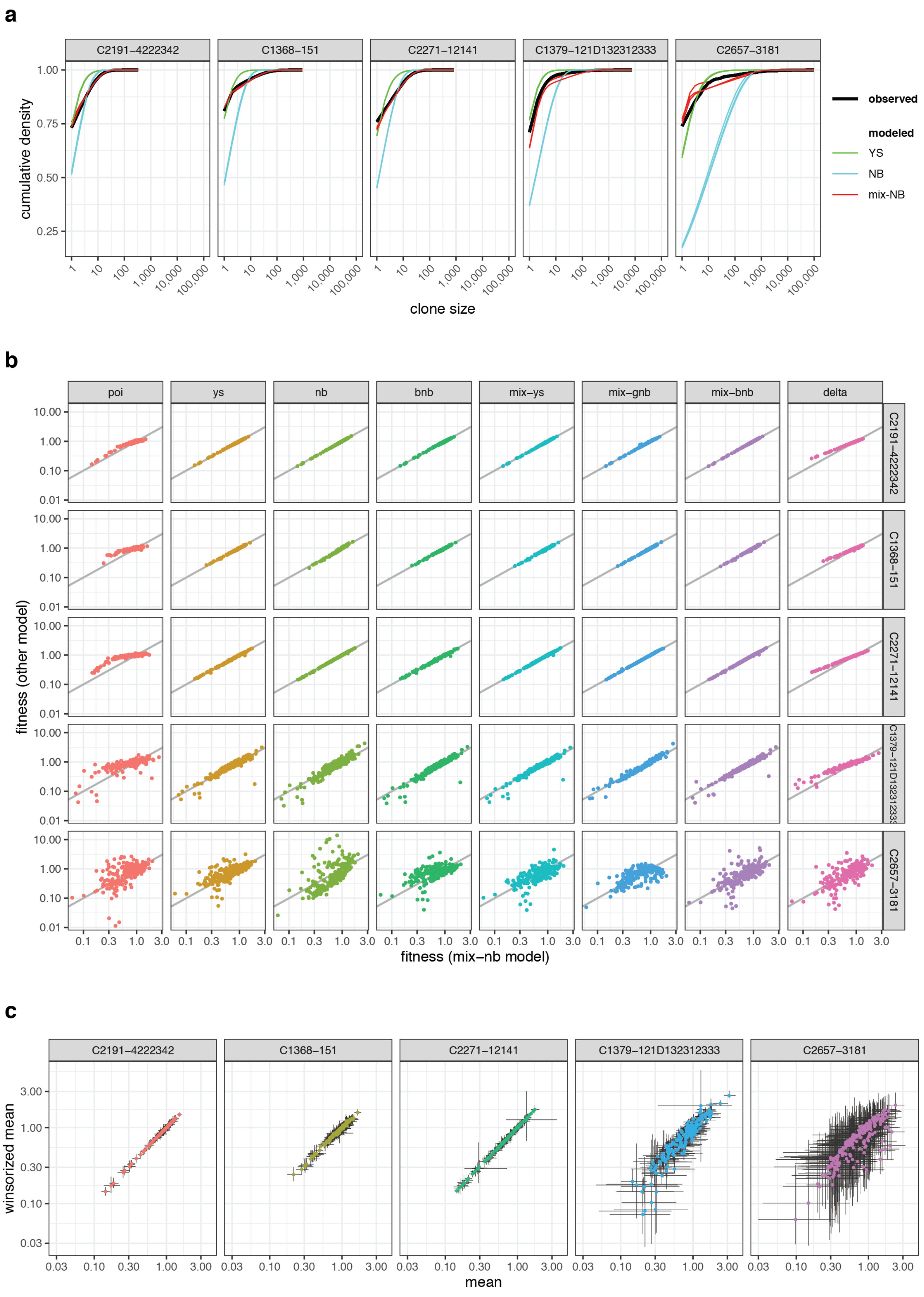

Figure S 4 **Model parameter testing**

Figure S 4 **a** Comparison of observed and fitted clone size distributions with different generative functions. Black line shows the observed cumulative clone size distribution, for all non-targeting guides combined, in a representative tumour from five PDX lines. Red line shows three MCMC samples fitting a mixture model of two negative-binomial distributions to the observed data. Green and cyan lines show MCMC samples fitting single Yule Simon or negative binomial distributions. The greater parametric space in the mixture model results in closer fit to the observed distribution in all five tumours. **b** Comparison of median fitness estimates from Bayesian mix-NB model vs other Bayesian or frequentist models. Scatter plots show median fitness estimates with different models in representative tumours from 5 PDX lines. Left 7 columns use a Bayesian model with a different generative function, the right column uses a frequentist delta model. The estimates are well correlated between models, with the exception of the poorly fitting Poisson model. The series are ordered from high Shannon diversity (top row) to low (bottom row). The model correlation becomes less strong due to sampling noise in tumours with lower diversity. (Model abbreviations: poi=Poisson, ys=Yule Simon, nb=negative binomial, bnb=beta negative binomial, mix=weighted linear mix of 2 distributions of the same model type but different parameters.) **c** Effect of winsorization of fitness estimates. Scatter plots compare the fitness estimates (median, bars=95% CI) with or without winsorization of the fitted distribution (mix-nb model, 0.98 winsorization cutoff). Data from a representative tumour from five PDX lines is shown. The effect of winsorization is greater in lower diversity tumour (right two panels).

Source data are provided as Source Data files.

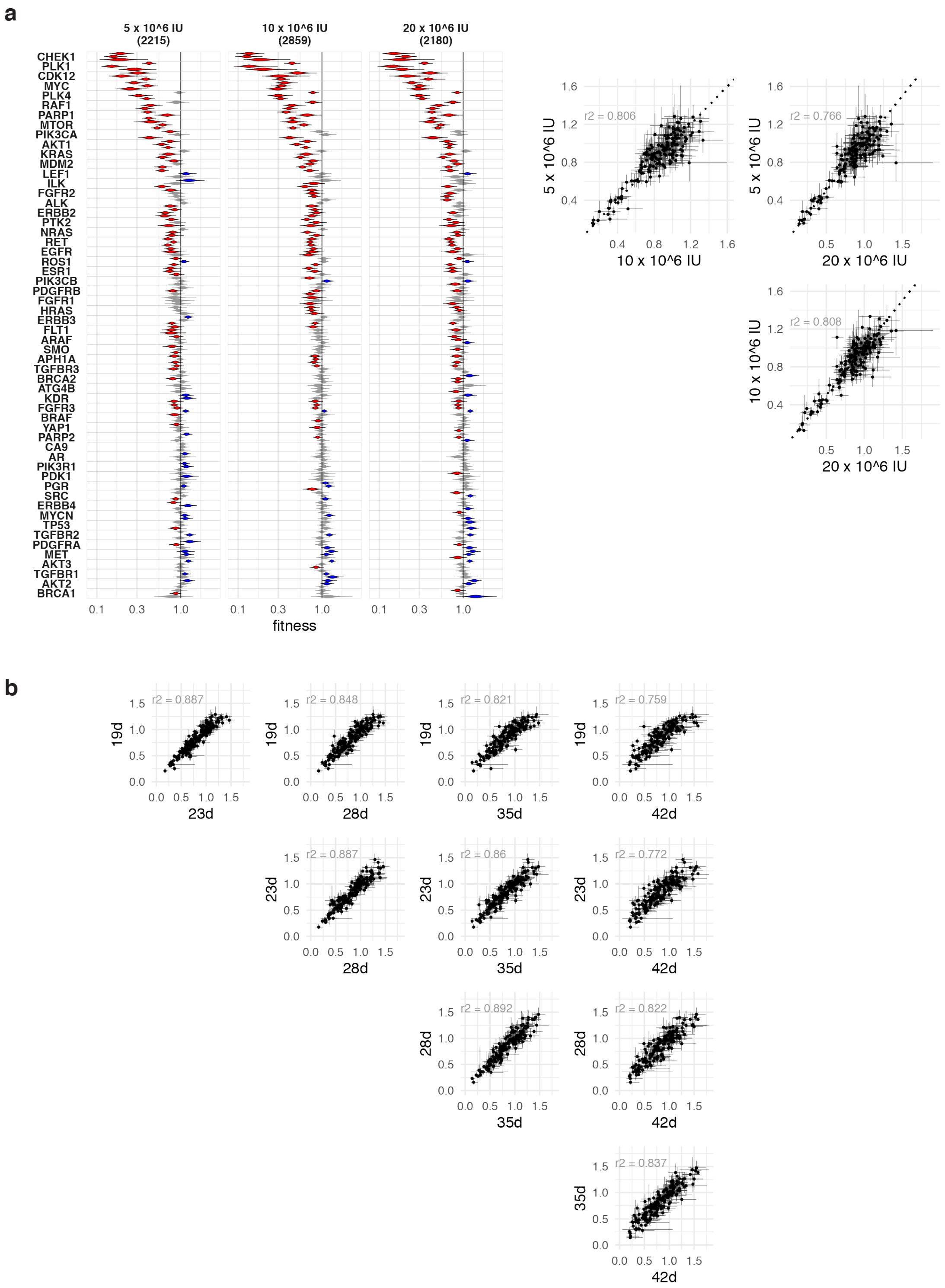

Figure S 5 Transplants at varying multiplicity of infection or propagation time *in vivo*

Figure S 5 **a** Left panel shows fitness estimates for three transplants using the same PDX material from series C2271 transduced with virus dose over a 5×10^6–20×10^6 infectiousunits range (1 PDX tumour per dose). Guide distributions coloured red (low fitness) and blue (high fitness) indicate estimates that differ from neutrality (fitness=1) with false discovery rate < 0.05 (Benjamini-Hochberg method). Right panel shows pairwise comparison of guide fitness estimates (median, bars=95% CI) between these transplants. **b** Pairwise comparison of guide fitness estimates (median, bars=95% CI) between transplants using the same PDX material from series C2271 harvested after varying times *in vivo* (1 PDX tumour for each of 5 timepoints).

Source data are provided as Source Data files.

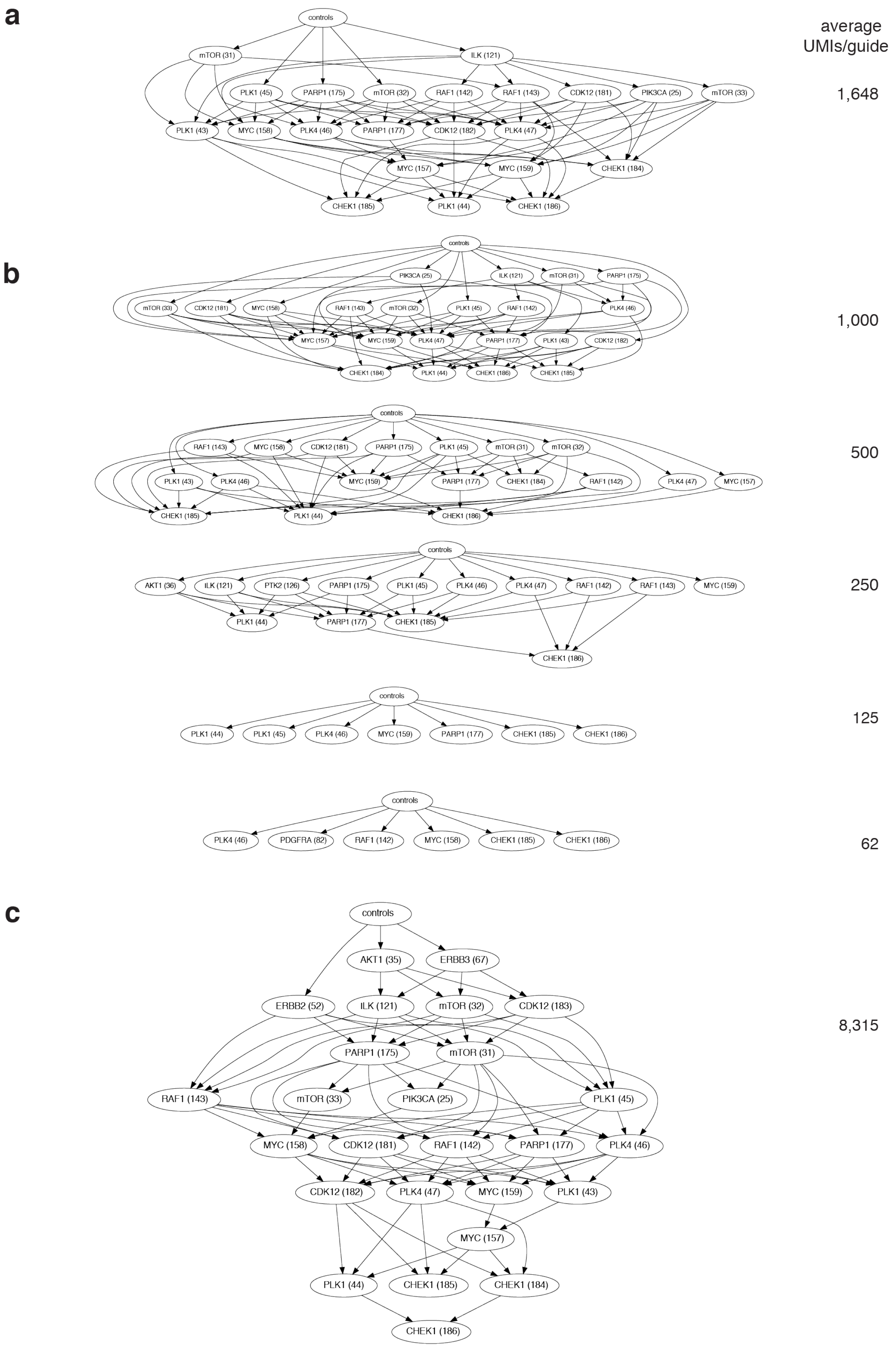

Figure S 6 Partially ordered set (poset) Hasse diagrams, with clone sub-sampling or dataset pooling

Figure S 6 **a** Partially ordered set (poset) Hasse diagram representation of guide fitness within dataset C2191-4222341. Nodes represent sgRNAs (annotated with gene target and index within the sgRNA library), with edges connecting a minimal set of nodes with non-overlapping 95% Bayesian credible intervals (fitness $a < b$, and no c such that $a < c < b$). The average number of unique UMIs per sgRNA is shown to the right of the diagram. **b** Hasse diagrams representing progressively smaller *in silico* random sub-sampling of clones from the dataset depicted in (a). As fewer clones are sampled, a smaller numbers of sgRNA pairs can be identified with differentially resolved fitness. **c** Hasse diagram representing grouped analysis of the dataset depicted in (a) together with three biological replicate datasets derived from transplants from the same initiating PDX. Pooling of replicates increases the number of sgRNA pairs with differentially resolved fitness

Source data are provided as Source Data files.

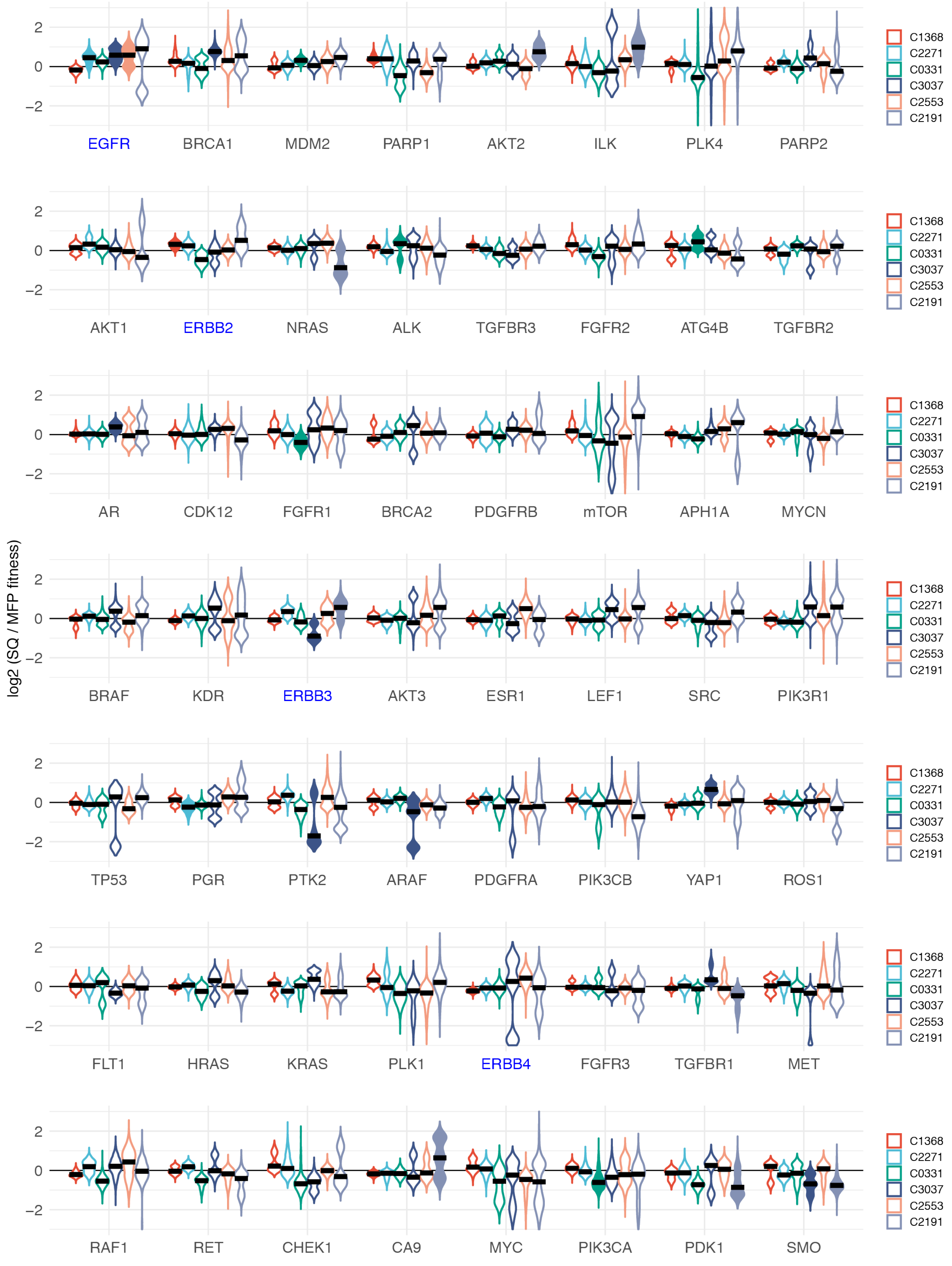

Figure S 7 Site fitness differences in six PDX series

Figure S 7 Posterior distribution of the fitness difference between subcutanous and mammary fat sites in six PDX lines (21 PDX tumours total), for all genes in the signaling library (three targeting guides per gene). Solid shading indicates comparisons satisfying outlying guide criteria. Genes are ordered based on median fitness difference (from highest relative sensitivity in mammary fat pad to highest relative sensitivity in subcutaneous site). *EGFR*/*ERBB* receptor genes are highlighted.

Source data are provided as Source Data files.

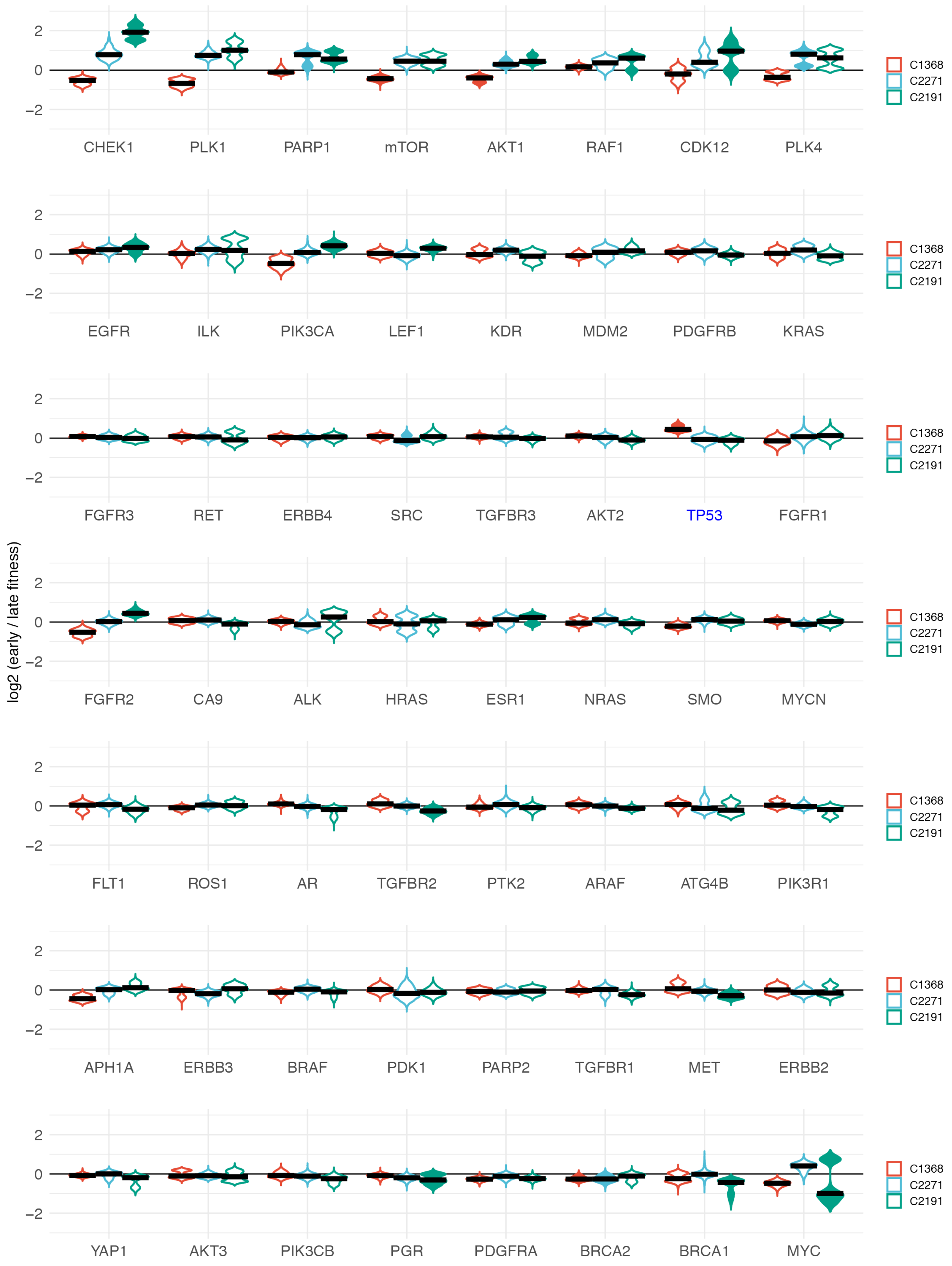

Figure S 8 Early vs late passage fitness differences in three PDX series

Figure S 8 Posterior distribution of the fitness difference between early and late PDX passages in three PDX lines (18 PDX tumours total), for all genes in the signaling library (three targeting guides per gene). Solid shading indicates comparisons satisfying outlying guide criteria. Genes are ordered based on median fitness difference (from highest relative sensitivity at late passage to highest relative sensitivity at early passage). *TP53* gene is highlighted.

Source data are provided as Source Data files.

Source data are provided as Source Data files.

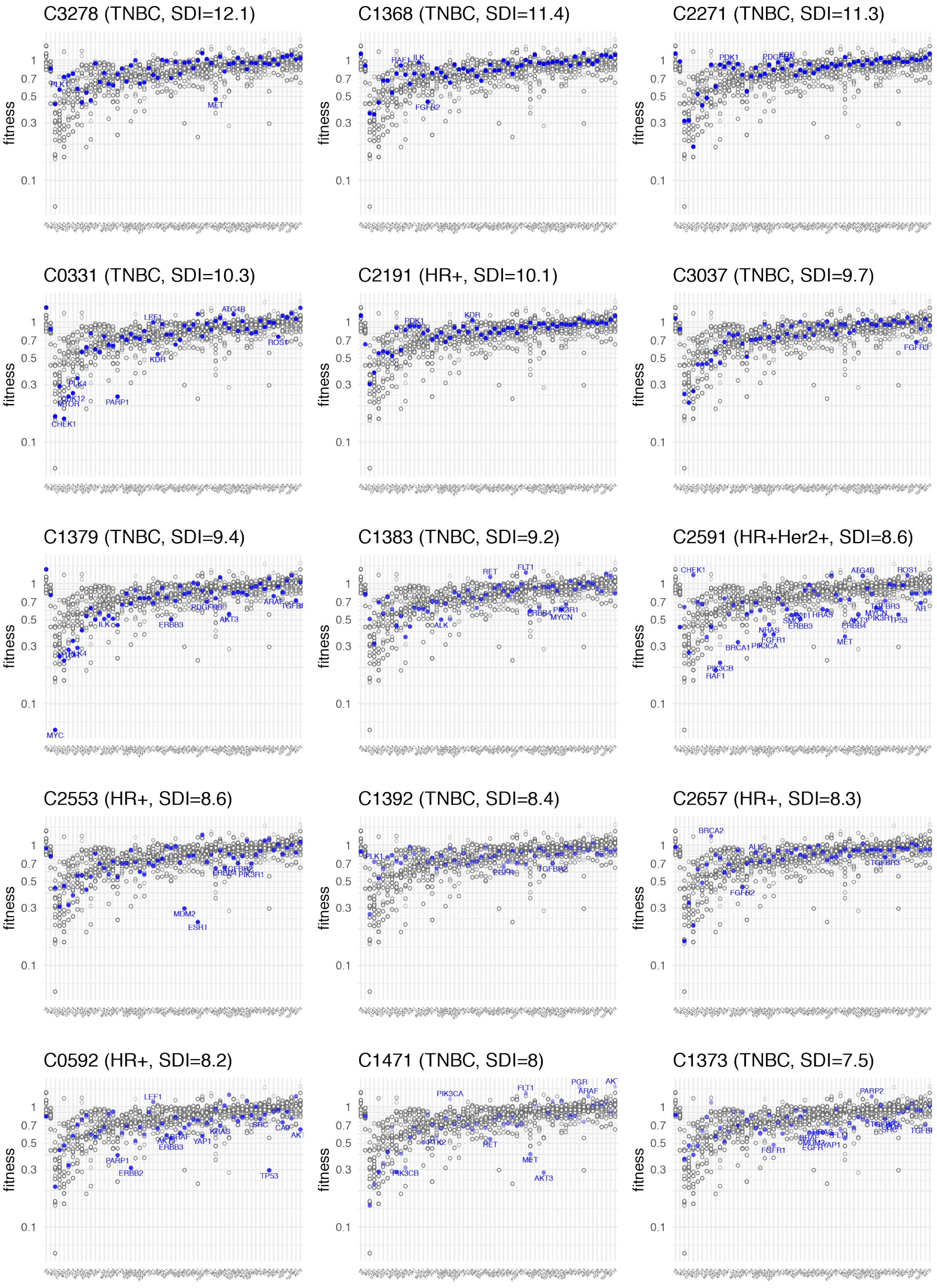

Figure S 9 Gene fitness by PDX series

Figure S 9 Gene fitness variation by PDX line. Panels show median gene fitness estimates (as main Fig 5a, 135 PDX tumours total), with points representing each series highlighted (blue). Gene points that lie more than two mean absolute deviations from the gene median are labeled. Panels are ordered from highest to lowest Shannon diversity.

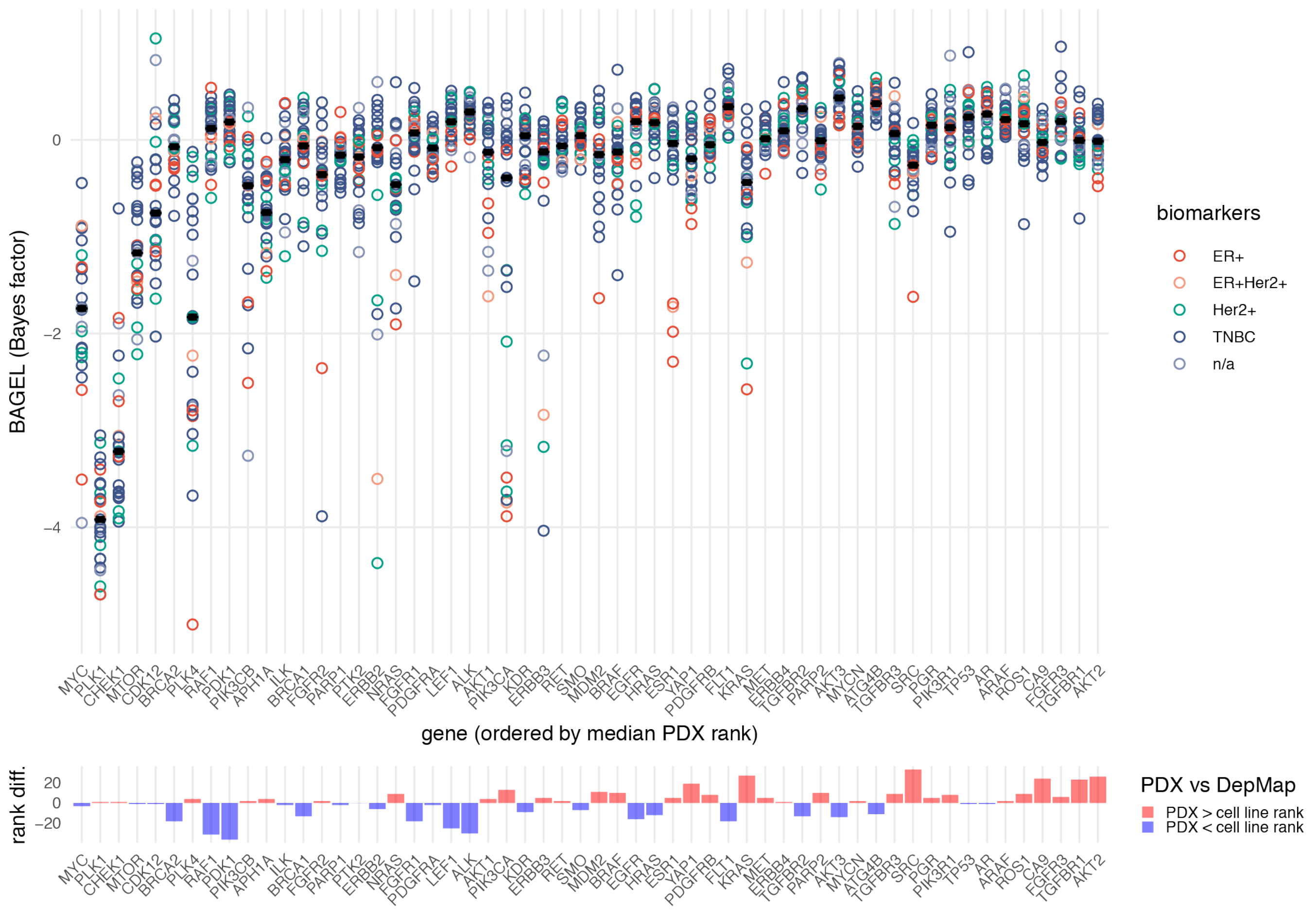

Figure S 10 **Quantitative assessment of gene fitness in cell lines**

Figure S 10 Fitness comparison between *in vivo* and *in vitro* datasets. Upper panel displays the fitness (BAGEL model) for 56 signaling genes from pooled in vitro CRISPR assays in breast cancer cell lines (Cancer Dependency Map, CCLE, Sanger Institute, n=25 lines). Genes are ordered as in main Fig 5a (i.e. in rank order from PDX-CRISPR assays). Lower panel shows the difference between the ranked position of each gene in PDX-CRISPR assays and this *in vitro* dataset with the ranked position within this study's in vivo dataset.

Source data are provided as Source Data files.

Supplementary Tables

Table S1 Details of CRISPR guides in each library.

Signaling1 Library

index	guide	sequence	gene	type
1	ctl_22	GAGTGATGCTTAGACTCCGT	ctl	non-targeting control
2	ctl_23	TTCGCACGATTGCACCTTGG	ctl	non-targeting control
3	ctl_24	CGCAAGGTGTCGGTAACCCT	ctl	non-targeting control
4	ctl_25	CGCTAGTACGCTCCTCTATA	ctl	non-targeting control
5	ctl_26	TTCCATTGGCTGGAATCTGA	ctl	non-targeting control
6	ctl_27	CGCGTGTAGCTGGAGACAAG	ctl	non-targeting control
7	ctl_28	ATTGAGAATTCGTTTCAAGG	ctl	non-targeting control
8	ctl_29	ATCAAAGTGTCTGACTTATT	ctl	non-targeting control
9	ctl_30	GTAAGGCCCGCGTACGAGCT	ctl	non-targeting control
10	ctl_31	CTTAAGGCGAGAAAAATTAG	ctl	non-targeting control
11	ctl_32	AAAGATATAGCAAATTATGG	ctl	non-targeting control
12	ctl_33	TTCTAAGCGCCCTGGGGACA	ctl	non-targeting control
13	ctl_34	CGGGTTATGTGATGCCATCT	ctl	non-targeting control
14	ctl_35	GCGGCGTCTGGGAATCGTTC	ctl	non-targeting control
15	ctl_36	TATCAATCGTCCGGGTCACT	ctl	non-targeting control
16	ctl_37	CATTGCACTCGTTGCCAACG	ctl	non-targeting control
17	ctl_38	CCCAGGTGTATAATGAAACG	ctl	non-targeting control
18	ctl_39	GTGCGTGAGTATTAACGCTC	ctl	non-targeting control
19	ctl_40	GTGTATGAATGTTAATTCCG	ctl	non-targeting control
20	ctl_41	TGCCCCACCCCAGTGCTCAG	ctl	non-targeting control
21	ctl_42	CAGCGCCGAAACTCTTTCCG	ctl	non-targeting control
22	ctl_43	CCGTTGGACTATGGCGGGTC	ctl	non-targeting control
23	ctl_44	TACTAACGCCGCTCCTACAG	ctl	non-targeting control
24	ctl_45	ACGCTGACGAGTAAAAGCGG	ctl	non-targeting control
25	PIK3CA_4	GTTCGAACAGGTATCTACCA	PIK3CA	gene-targeting
26	PIK3CA_5	TGGTCTGTATCCCGAGAAGC	PIK3CA	gene-targeting
27	PIK3CA_6	AGTTCACCTGATGATGGTCG	PIK3CA	gene-targeting
28	PIK3CB_4	AAAGAGCACTTGGTAATCGG	PIK3CB	gene-targeting
29	PIK3CB_5	GATGAGGTATCTTCTGCAGG	PIK3CB	gene-targeting
30	PIK3CB_6	TCATCCTTGAAGCATACTGC	PIK3CB	gene-targeting
31	mTOR_4	GAATAGGCTCCATGTAGGGG	mTOR	gene-targeting
32	mTOR_5	GAGCCGGAATGAGGAAACCA	mTOR	gene-targeting
33	mTOR_6	CAGCTCGTTAAGGATCAACA	mTOR	gene-targeting
34	AKT1_4	GAAGGTGCGTTCGATGACAG	AKT1	gene-targeting
35	AKT1_5	GGGCAAGGGCACTTTCGGCA	AKT1	gene-targeting
36	AKT1_6	AGGTGCCATCATTCTTGAGG	AKT1	gene-targeting
37	AKT2_4	ACGGAGAAGTTGTTTAAGGG	AKT2	gene-targeting
38	AKT2_5	ATGACAAAGGTGTTGGGTCG	AKT2	gene-targeting
39	AKT2_6	AAGTGGCGGTCAGCAAGGCA	AKT2	gene-targeting

Signaling1 Library

index	guide	sequence	gene	type
40	AKT3_4	AAGAAGGTTGGGTTCAGAAG	AKT3	gene-targeting
41	AKT3_5	TAAGGTAAATCCACATCTTG	AKT3	gene-targeting
42	AKT3_6	TTTTCCATTTGTCGAGAGAG	AKT3	gene-targeting
43	PLK1_4	AACCAAAGTCGAATATGACG	PLK1	gene-targeting
44	PLK1_5	AGCCAAGCACAATTTGCCGT	PLK1	gene-targeting
45	PLK1_6	GTTAATGGTTGGGCGGGCAG	PLK1	gene-targeting
46	PLK4_4	ACTGTGTCAGTGTCGAAGGG	PLK4	gene-targeting
47	PLK4_5	CAGAGGAATAAGCTCTACGA	PLK4	gene-targeting
48	PLK4_6	TGAGGTGTCGGGTCTGCAAA	PLK4	gene-targeting
49	EGFR_4	AGGGTTGTTGCTGAACCGCA	EGFR	gene-targeting
50	EGFR_5	AGCGATGCGACCCTCCGGGA	EGFR	gene-targeting
51	EGFR_6	AGCTATGAGATGGAGGAAGA	EGFR	gene-targeting
52	ERBB2_4	CCGGCACAGACATGAAGCTG	ERBB2	gene-targeting
53	ERBB2_5	TGAGTCCATGCCCAATCCCG	ERBB2	gene-targeting
54	ERBB2_6	AAGGAGGGGTCTTGATCCAG	ERBB2	gene-targeting
55	ESR1_4	CTCGGGGTAGTTGTACACGG	ESR1	gene-targeting
56	ESR1_5	GCAAAAGTATTACATCACGG	ESR1	gene-targeting
57	ESR1_6	TCAGATAATCGACGCCAGGG	ESR1	gene-targeting
58	SMO_4	CTCTGGTCGGGTAAGTGCGG	SMO	gene-targeting
59	SMO_5	CACGGCAGACGATCTCTCGG	SMO	gene-targeting
60	SMO_6	GCCCAAGTGTGAGAATGACC	SMO	gene-targeting
61	LEF1_4	TCAGGAGCCCTACCACGACA	LEF1	gene-targeting
62	LEF1_5	CGCATGGGATGGCTGCACCA	LEF1	gene-targeting
63	LEF1_6	GATCGCCCTCGTCCTTGAAG	LEF1	gene-targeting
64	APH1A_4	GGCTGCGCTGAATACTTCGG	APH1A	gene-targeting
65	APH1A_5	ACCAGGATGATAACGCGAAG	APH1A	gene-targeting
66	APH1A_6	GACACCACTGATGATACCGA	APH1A	gene-targeting
67	ERBB3_1	CACTGTACAAGCTCTACGAG	ERBB3	gene-targeting
68	ERBB3_2	CTTGCAAACCTCATGACAGG	ERBB3	gene-targeting
69	ERBB3_3	TTCTCTACTGGCGTGGGCGC	ERBB3	gene-targeting
70	ERBB4_1	AAAGCCACAGCTTTACCCGC	ERBB4	gene-targeting
71	ERBB4_2	TTATGAGGATCGATATGCCT	ERBB4	gene-targeting
72	ERBB4_3	GCTTTACCCGCAGGAAGGAG	ERBB4	gene-targeting
73	MET_1	TCAGCTTCCCAACTTCACCG	MET	gene-targeting
74	MET_2	GTCAACGCGCTGCAAAGCTG	MET	gene-targeting
75	MET_3	TCCATTGGTTCGGCAGAATC	MET	gene-targeting
76	FLT1_1	CTTACCATATATATGCACTG	FLT1	gene-targeting
77	FLT1_2	TATACTTGTCGTGTAAGGAG	FLT1	gene-targeting
78	FLT1_3	TTAGGTGACGTAACCCGGCA	FLT1	gene-targeting
79	KDR_1	GGGCTCCTTACCCACAGAGG	KDR	gene-targeting
80	KDR_2	GGTAACCAAGGTACTTCGCA	KDR	gene-targeting
81	KDR_3	CTGCACAGGTGTACAATCCT	KDR	gene-targeting
82	PDGFRA_1	CTAAGACCAGGAACGCCGGA	PDGFRA	gene-targeting
83	PDGFRA_2	GGATAGAGGGTAATGAAAGC	PDGFRA	gene-targeting

Signaling1 Library

index	guide	sequence	gene	type
84	PDGFRA_3	TCGGGATCAGTTGTGCGACA	PDGFRA	gene-targeting
85	PDGFRB_1	CATTGTGATCGGGAATGAGG	PDGFRB	gene-targeting
86	PDGFRB_2	GTTCCTCGGCATCATTAGGG	PDGFRB	gene-targeting
87	PDGFRB_3	TAACTCTGCAGGACACCATG	PDGFRB	gene-targeting
88	ALK_1	AGACAAGCTGCGGTTTCCAC	ALK	gene-targeting
89	ALK_2	GTGGCTCGGGACATTGCCTG	ALK	gene-targeting
90	ALK_3	CATCCTGCTGGAGCTCATGG	ALK	gene-targeting
91	ROS1_1	AAGGCTGTGTCTACTTGGAA	ROS1	gene-targeting
92	ROS1_2	CGCAGAGTCAGTTTTTCCCG	ROS1	gene-targeting
93	ROS1_3	AGGGGCTTGACCACATAGGA	ROS1	gene-targeting
94	RET_1	TGGCGTACTCCACGATGAGG	RET	gene-targeting
95	RET_2	GTCCCGAGATGTTTATGAAG	RET	gene-targeting
96	RET_3	GAAGAAGGAGAAGTATACGC	RET	gene-targeting
97	FGFR1_1	AAGTTCAAATGCCCTTCCAG	FGFR1	gene-targeting
98	FGFR1_2	GATCCGGTCAAATAATGCCT	FGFR1	gene-targeting
99	FGFR1_3	TCAGTGCATACACCGAGACC	FGFR1	gene-targeting
100	FGFR2_1	GCAGCGAAACTTGACAGTGT	FGFR2	gene-targeting
101	FGFR2_2	TTCCTCTACATTTGCAGGGG	FGFR2	gene-targeting
102	FGFR2_3	CGTGTTGTTATCCTCACCAG	FGFR2	gene-targeting
103	FGFR3_1	CAGGTCGTGTGTGCAGTTGG	FGFR3	gene-targeting
104	FGFR3_2	AAGGTGAGCTGCTCCTCGGG	FGFR3	gene-targeting
105	FGFR3_3	CTCGGGAGATGACGAAGACG	FGFR3	gene-targeting
106	PIK3R1_1	ACGAGCTTGATAAGAAGAGG	PIK3R1	gene-targeting
107	PIK3R1_2	AAACTTCGAGATACAGCAGA	PIK3R1	gene-targeting
108	PIK3R1_3	AGCGTAAGCCAATACTGATG	PIK3R1	gene-targeting
109	PDK1_1	GCGTAGAAGTCCACCTGGCC	PDK1	gene-targeting
110	PDK1_2	CCGTTCAATTGGTACAAAGC	PDK1	gene-targeting
111	PDK1_3	ACATACCAGAGGCACTGCGC	PDK1	gene-targeting
112	PGR_1	GAAGCTTGACAAACTCCTGT	PGR	gene-targeting
113	PGR_2	TTGTGTGTGATGCAAGACAG	PGR	gene-targeting
114	PGR_3	AGATCCTACAAACACGTCAG	PGR	gene-targeting
115	AR_1	GTATCCGAAGGCAGCAGCAG	AR	gene-targeting
116	AR_2	CAGCAGCAGCAAACTGGCGC	AR	gene-targeting
117	AR_3	CCTTAAAGACATCCTGAGCG	AR	gene-targeting
118	SRC_1	ATCGGCGTGTTCTGAGGAGG	SRC	gene-targeting
119	SRC_2	GAACCTGGTGTGCAAAGTGG	SRC	gene-targeting
120	SRC_3	TCAATGCAGAGAACCCGAGA	SRC	gene-targeting
121	ILK_1	CGGAGAACGACCTCAACCAG	ILK	gene-targeting
122	ILK_2	GGATCAATGTAATGAACCGT	ILK	gene-targeting
123	ILK_3	CGGATCAATGTAATGAACCG	ILK	gene-targeting
124	PTK2_1	GCGAGGTTCCATTCACCAGC	PTK2	gene-targeting
125	PTK2_2	TGGGCCAGTATTATCAGGCA	PTK2	gene-targeting
126	PTK2_3	AGTGAACCTCCTCTGACCGC	PTK2	gene-targeting
127	KRAS_1	GTAGTTGGAGCTGGTGGCGT	KRAS	gene-targeting

Signaling1 Library

index	guide	sequence	gene	type
128	KRAS_2	TCTCGACACAGCAGGTCAAG	KRAS	gene-targeting
129	KRAS_3	TTGGATATTCTCGACACAGC	KRAS	gene-targeting
130	NRAS_1	AGAGACCAATACATGAGGAC	NRAS	gene-targeting
131	NRAS_2	CCTTGTTACATCACCACACA	NRAS	gene-targeting
132	NRAS_3	GACTGAGTACAAACTGGTGG	NRAS	gene-targeting
133	HRAS_1	TCTCAGGGAGCAGATCAAAC	HRAS	gene-targeting
134	HRAS_2	ATTCCGTCATCGCTCCTCAG	HRAS	gene-targeting
135	HRAS_3	TGATGGGGAGACGTGCCTGT	HRAS	gene-targeting
136	ARAF_1	AGTGTCCAGGATTTGTCCGG	ARAF	gene-targeting
137	ARAF_2	ATGAGCTCCTCGCCATCCAG	ARAF	gene-targeting
138	ARAF_3	AGAAAGTGGTATGCTCGAGG	ARAF	gene-targeting
139	BRAF_1	ATACCCAATAGAGTCCGAGG	BRAF	gene-targeting
140	BRAF_2	GGCTCTCGGTTATAAGATGG	BRAF	gene-targeting
141	BRAF_3	GCCATCTTATAACCGAGAGC	BRAF	gene-targeting
142	RAF1_1	GCCGAACAAGCAAAGAACAG	RAF1	gene-targeting
143	RAF1_2	CGTGCCAGCACAAAGAGAGC	RAF1	gene-targeting
144	RAF1_3	GACATCGAAATCCATTGAGC	RAF1	gene-targeting
145	TGFBR1_1	TGGCAGAAACACTGTAACGC	TGFBR1	gene-targeting
146	TGFBR1_2	CGATTTGGAGAAGTTTGGAG	TGFBR1	gene-targeting
147	TGFBR1_3	CCATCGAGTGCCAAATGAAG	TGFBR1	gene-targeting
148	TGFBR2_1	TGTGGAGGTGAGCAATCCCC	TGFBR2	gene-targeting
149	TGFBR2_2	GGGGAAAGGTCGCTTTGCTG	TGFBR2	gene-targeting
150	TGFBR2_3	CAGAGCCATGGAGTAGACAT	TGFBR2	gene-targeting
151	TGFBR3_1	AATTGGGCCCGAAAAGAGTA	TGFBR3	gene-targeting
152	TGFBR3_2	ATAGTAGACCACACCATCAA	TGFBR3	gene-targeting
153	TGFBR3_3	GTCTTCAGATGCCACACCAG	TGFBR3	gene-targeting
154	YAP1_1	GCACGATCTGATGCCCGGCG	YAP1	gene-targeting
155	YAP1_2	ATCAGATCGTGCACGTCCGC	YAP1	gene-targeting
156	YAP1_3	ACGAGGTTACCTGTCGGGAG	YAP1	gene-targeting
157	MYC_1	GCCGTATTTCTACTGCGACG	MYC	gene-targeting
158	MYC_2	CTCTGAGACGAGCTTGGCGG	MYC	gene-targeting
159	MYC_3	AGGGTAGGGGAAGACCACCG	MYC	gene-targeting
160	MYCN_1	GGGGGGATGACACTCTTGAG	MYCN	gene-targeting
161	MYCN_2	CGCGCTTGTTCACGGGAAAG	MYCN	gene-targeting
162	MYCN_3	GGGAAAGGGGAAGACCACGG	MYCN	gene-targeting
163	MDM2_1	GTGGTTACAGCACCATCAGT	MDM2	gene-targeting
164	MDM2_2	AGGGTCTCTTGTTCCGAAGC	MDM2	gene-targeting
165	MDM2_3	CACCTCACAGATTCCAGCTT	MDM2	gene-targeting
166	TP53_1	GTTGCAAACCAGACCTCAGG	TP53	gene-targeting
167	TP53_2	GCTTGTAGATGGCCATGGCG	TP53	gene-targeting
168	TP53_3	GTTGATTCCACACCCCCGCC	TP53	gene-targeting
169	BRCA1_1	TCTTGTGCTGACTTACCAGA	BRCA1	gene-targeting
170	BRCA1_2	CAGTTCCAAGGTTAGAGAGT	BRCA1	gene-targeting
171	BRCA1_3	GTTTCTATCATCCAAAGTAT	BRCA1	gene-targeting

Signaling1 Library

index	guide	sequence	gene	type
172	BRCA2_1	GAGCACAGTAGAACTAAGGG	BRCA2	gene-targeting
173	BRCA2_2	AAGACACGCTGCAACAAAGC	BRCA2	gene-targeting
174	BRCA2_3	CTGTCTACCTGACCAATCGA	BRCA2	gene-targeting
175	PARP1_1	TCCAACAGAAGTACGTGCAA	PARP1	gene-targeting
176	PARP1_2	CTTCCCTGCGAGGAATGCTC	PARP1	gene-targeting
177	PARP1_3	CGAGTCGAGTACGCCAAGAG	PARP1	gene-targeting
178	PARP2_1	ATTGAGGATTGTATTCGGGC	PARP2	gene-targeting
179	PARP2_2	TCAGCGTTCGAATTCCATGG	PARP2	gene-targeting
180	PARP2_3	TCTACGAGTTTTCTTGGCAG	PARP2	gene-targeting
181	CDK12_1	GTCTTTGAGTGAGTAGAAGG	CDK12	gene-targeting
182	CDK12_2	TGGCCTTCAAACTAGACCGA	CDK12	gene-targeting
183	CDK12_3	GCTTGTGCTTCGATACCAAG	CDK12	gene-targeting
184	CHEK1_1	CATAAGGAAAGACCTGTGCG	CHEK1	gene-targeting
185	CHEK1_2	TTTCTGGAGTACTGTAGTGG	CHEK1	gene-targeting
186	CHEK1_3	CTTACTGCAATGCTCGCTGG	CHEK1	gene-targeting
187	ATG4B_1	GTTGGCGAAGGCAAGTCCAT	ATG4B	gene-targeting
188	ATG4B_2	GCGCAAATGGGAGTTGGCGA	ATG4B	gene-targeting
189	ATG4B_3	TTTCTACCCAGTATCCAAAC	ATG4B	gene-targeting
190	CA9_1	CTGCGCAACAATGGCCACAG	CA9	gene-targeting
191	CA9_2	GGTAGATCCTCCTCTCCGGG	CA9	gene-targeting
192	CA9_3	CAGAGTCATTGGCGCTATGG	CA9	gene-targeting

Signaling2 Library

index	guide	sequence	gene	type
1	ctl_t_1	GAGTCCGATGTTCCAGCACG	ctl	non-gene-targeting control
2	ctl_t_2	ATCGAAGGTAGAATTGCAAG	ctl	non-gene-targeting control
3	ctl_t_3	ACTGGATTCACTGTACTCAT	ctl	non-gene-targeting control
4	ctl_t_4	CCAACATTTAGGCTAACAGA	ctl	non-gene-targeting control
5	ctl_t_5	ATATGCGTTCCTACATTCGT	ctl	non-gene-targeting control
6	ctl_t_6	TGTTGTCAAGCAGAGGGCAA	ctl	non-gene-targeting control
7	ctl_t_7	GTTTGCCAAAGAAAGTGACA	ctl	non-gene-targeting control
8	ctl_t_8	TGCCTGAACGCCATGCACAG	ctl	non-gene-targeting control
9	ctl_t_9	ACCCTTTCGCCCCAAATCAG	ctl	non-gene-targeting control
10	ctl_t_10	GTGGCCATGTGATTCAGGGT	ctl	non-gene-targeting control
11	ctl_t_11	ACCTATCTTAGGCTTTCCCG	ctl	non-gene-targeting control
12	ctl_t_12	TCAGAGAAGACATCCGCCAG	ctl	non-gene-targeting control
13	ctl_t_13	TGGCTGTGGTCCAAGAGTGG	ctl	non-gene-targeting control
14	ctl_t_14	GTTTGGAGGGCAGAACGAAT	ctl	non-gene-targeting control
15	ctl_t_15	CCACAATCCAGATAGGAAGG	ctl	non-gene-targeting control
16	ctl_t_16	TAGCAAAGCCACACCAGCAA	ctl	non-gene-targeting control
17	ctl_t_17	GCTCACTTTGGAGCAACACC	ctl	non-gene-targeting control
18	ctl_t_18	TGTTATGGTTCTCTGCTCAG	ctl	non-gene-targeting control

Signaling2 Library

index	guide	sequence	gene	type
19	ctl_t_19	CTCTTAGTACACAAGGTCAG	ctl	non-gene-targeting control
20	ctl_t_20	GAGTTCTCCACATAAACCTA	ctl	non-gene-targeting control
21	ctl_t_21	GGGTGGTGAACCTTGCCTGG	ctl	non-gene-targeting control
22	ctl_t_22	CCCTTTATACCCATTCCAGA	ctl	non-gene-targeting control
23	ctl_t_23	TCCCAAGGGACATAACGCCT	ctl	non-gene-targeting control
24	ctl_t_24	TCCTCTGTAAATATTCCTGA	ctl	non-gene-targeting control
25	PIK3CA_4	GTTCGAACAGGTATCTACCA	PIK3CA	gene-targeting
26	PIK3CA_1	GAAGCTGTATAATGCTTGGG	PIK3CA	gene-targeting
27	PIK3CA_6	AGTTCACCTGATGATGGTCG	PIK3CA	gene-targeting
28	PIK3CB_4	AAAGAGCACTTGGTAATCGG	PIK3CB	gene-targeting
29	PIK3CB_7	TGATACCTCTGAGCTTACGA	PIK3CB	gene-targeting
30	PIK3CB_6	TCATCCTTGAAGCATACTGC	PIK3CB	gene-targeting
31	mTOR_4	GAATAGGCTCCATGTAGGGG	mTOR	gene-targeting
32	mTOR_5	GAGCCGGAATGAGGAAACCA	mTOR	gene-targeting
33	mTOR_6	CAGCTCGTTAAGGATCAACA	mTOR	gene-targeting
34	AKT1_4	GAAGGTGCGTTCGATGACAG	AKT1	gene-targeting
35	AKT1_5	GGGCAAGGGCACTTTCGGCA	AKT1	gene-targeting
36	AKT1_6	AGGTGCCATCATTCTTGAGG	AKT1	gene-targeting
37	AKT2_4	ACGGAGAAGTTGTTTAAGGG	AKT2	gene-targeting
38	AKT2_5	ATGACAAAGGTGTTGGGTCG	AKT2	gene-targeting
39	AKT2_6	AAGTGGCGGTCAGCAAGGCA	AKT2	gene-targeting
40	AKT3_4	AAGAAGGTTGGGTTCAGAAG	AKT3	gene-targeting
41	AKT3_5	TAAGGTAAATCCACATCTTG	AKT3	gene-targeting
42	AKT3_3	GCCACAGAATGTCTTCATGG	AKT3	gene-targeting
43	PLK1_4	AACCAAAGTCGAATATGACG	PLK1	gene-targeting
44	PLK1_5	AGCCAAGCACAATTTGCCGT	PLK1	gene-targeting
45	PLK1_2	AGGATTCCACGGCTTTTTCG	PLK1	gene-targeting
46	PLK4_2	GGTGAGCATACTTGATTCAG	PLK4	gene-targeting
47	PLK4_5	CAGAGGAATAAGCTCTACGA	PLK4	gene-targeting
48	PLK4_7	TCATGCACCAGATCATCACA	PLK4	gene-targeting
49	EGFR_4	AGGGTTGTTGCTGAACCGCA	EGFR	gene-targeting
50	EGFR_5	AGCGATGCGACCCTCCGGGA	EGFR	gene-targeting
51	EGFR_6	AGCTATGAGATGGAGGAAGA	EGFR	gene-targeting
52	ERBB2_4	CCGGCACAGACATGAAGCTG	ERBB2	gene-targeting
53	ERBB2_5	TGAGTCCATGCCCAATCCCG	ERBB2	gene-targeting
54	ERBB2_1	GTCACCTCTTGGTTGTGCAG	ERBB2	gene-targeting
55	ESR1_4	CTCGGGGTAGTTGTACACGG	ESR1	gene-targeting
56	ESR1_5	GCAAAAGTATTACATCACGG	ESR1	gene-targeting
57	ESR1_6	TCAGATAATCGACGCCAGGG	ESR1	gene-targeting
58	SMO_4	CTCTGGTCGGGTAAGTGCGG	SMO	gene-targeting
59	SMO_5	CACGGCAGACGATCTCTCGG	SMO	gene-targeting
60	SMO_6	GCCCAAGTGTGAGAATGACC	SMO	gene-targeting
61	LEF1_4	TCAGGAGCCCTACCACGACA	LEF1	gene-targeting
62	LEF1_7	GTAGGGATATCAGGAGCTGG	LEF1	gene-targeting

Signaling2 Library

index	guide	sequence	gene	type
63	LEF1_6	GATCGCCCTCGTCCTTGAAG	LEF1	gene-targeting
64	APH1A_4	GGCTGCGCTGAATACTTCGG	APH1A	gene-targeting
65	APH1A_5	ACCAGGATGATAACGCGAAG	APH1A	gene-targeting
66	APH1A_6	GACACCACTGATGATACCGA	APH1A	gene-targeting
67	ERBB3_1	CACTGTACAAGCTCTACGAG	ERBB3	gene-targeting
68	ERBB3_2	CTTGCAAACCTCATGACAGG	ERBB3	gene-targeting
69	ERBB3_3	TTCTCTACTGGCGTGGGCGC	ERBB3	gene-targeting
70	ERBB4_1	AAAGCCACAGCTTTACCCGC	ERBB4	gene-targeting
71	ERBB4_2	TTATGAGGATCGATATGCCT	ERBB4	gene-targeting
72	ERBB4_3	GCTTTACCCGCAGGAAGGAG	ERBB4	gene-targeting
73	MET_1	TCAGCTTCCCAACTTCACCG	MET	gene-targeting
74	MET_2	GTCAACGCGCTGCAAAGCTG	MET	gene-targeting
75	MET_4	GCTAATCTTGGGACATCAGA	MET	gene-targeting
76	FLT1_4	TCAGGGATCAAAGTGTCAAG	FLT1	gene-targeting
77	FLT1_2	TATACTTGTCGTGTAAGGAG	FLT1	gene-targeting
78	FLT1_3	TTAGGTGACGTAACCCGGCA	FLT1	gene-targeting
79	KDR_1	GGGCTCCTTACCCACAGAGG	KDR	gene-targeting
80	KDR_2	GGTAACCAAGGTACTTCGCA	KDR	gene-targeting
81	KDR_4	ACACCTGTGCAGCATCCAGT	KDR	gene-targeting
82	PDGFRA_1	CTAAGACCAGGAACGCCGGA	PDGFRA	gene-targeting
83	PDGFRA_2	GGATAGAGGGTAATGAAAGC	PDGFRA	gene-targeting
84	PDGFRA_3	TCGGGATCAGTTGTGCGACA	PDGFRA	gene-targeting
85	PDGFRB_1	CATTGTGATCGGGAATGAGG	PDGFRB	gene-targeting
86	PDGFRB_2	GTTCCTCGGCATCATTAGGG	PDGFRB	gene-targeting
87	PDGFRB_4	GGTTCGCGTGAAGGTGGCAG	PDGFRB	gene-targeting
88	ALK_1	AGACAAGCTGCGGTTTCCAC	ALK	gene-targeting
89	ALK_2	GTGGCTCGGGACATTGCCTG	ALK	gene-targeting
90	ALK_3	CATCCTGCTGGAGCTCATGG	ALK	gene-targeting
91	ROS1_1	AAGGCTGTGTCTACTTGGAA	ROS1	gene-targeting
92	ROS1_4	AGGCTGTGTCTGTAGTACAA	ROS1	gene-targeting
93	ROS1_3	AGGGGCTTGACCACATAGGA	ROS1	gene-targeting
94	RET_1	TGGCGTACTCCACGATGAGG	RET	gene-targeting
95	RET_2	GTCCCGAGATGTTTATGAAG	RET	gene-targeting
96	RET_3	GAAGAAGGAGAAGTATACGC	RET	gene-targeting
97	FGFR1_1	AAGTTCAAATGCCCTTCCAG	FGFR1	gene-targeting
98	FGFR1_2	GATCCGGTCAAATAATGCCT	FGFR1	gene-targeting
99	FGFR1_3	TCAGTGCATACACCGAGACC	FGFR1	gene-targeting
100	FGFR2_1	GCAGCGAAACTTGACAGTGT	FGFR2	gene-targeting
101	FGFR2_2	TTCCTCTACATTTGCAGGGG	FGFR2	gene-targeting
102	FGFR2_3	CGTGTTGTTATCCTCACCAG	FGFR2	gene-targeting
103	FGFR3_4	CATCGCAAGGCTGTCCTCAG	FGFR3	gene-targeting
104	FGFR3_2	AAGGTGAGCTGCTCCTCGGG	FGFR3	gene-targeting
105	FGFR3_3	CTCGGGAGATGACGAAGACG	FGFR3	gene-targeting
106	PIK3R1_1	ACGAGCTTGATAAGAAGAGG	PIK3R1	gene-targeting

Signaling2 Library

index	guide	sequence	gene	type
107	PIK3R1_4	TCCAGTATCCAAATACCAAC	PIK3R1	gene-targeting
108	PIK3R1_3	AGCGTAAGCCAATACTGATG	PIK3R1	gene-targeting
109	PDK1_4	ATACAAGGAGAGCTTTGGGG	PDK1	gene-targeting
110	PDK1_2	CCGTTCAATTGGTACAAAGC	PDK1	gene-targeting
111	PDK1_3	ACATACCAGAGGCACTGCGC	PDK1	gene-targeting
112	PGR_1	GAAGCTTGACAAACTCCTGT	PGR	gene-targeting
113	PGR_2	TTGTGTGTGATGCAAGACAG	PGR	gene-targeting
114	PGR_4	AGTCAGAGTTGTGAGAGCAC	PGR	gene-targeting
115	AR_1	GTATCCGAAGGCAGCAGCAG	AR	gene-targeting
116	AR_2	CAGCAGCAGCAAACTGGCGC	AR	gene-targeting
117	AR_3	CCTTAAAGACATCCTGAGCG	AR	gene-targeting
118	SRC_1	ATCGGCGTGTTCTGAGGAGG	SRC	gene-targeting
119	SRC_4	TGTCCTTCAAGAAAGGCGAG	SRC	gene-targeting
120	SRC_3	TCAATGCAGAGAACCCGAGA	SRC	gene-targeting
121	ILK_1	CGGAGAACGACCTCAACCAG	ILK	gene-targeting
122	ILK_2	GGATCAATGTAATGAACCGT	ILK	gene-targeting
123	ILK_4	CCTGTCCTGCAGCTATGGAA	ILK	gene-targeting
124	PTK2_1	GCGAGGTTCCATTCACCAGC	PTK2	gene-targeting
125	PTK2_4	CGATCATACTGGGAGATGCG	PTK2	gene-targeting
126	PTK2_3	AGTGAACCTCCTCTGACCGC	PTK2	gene-targeting
127	KRAS_1	GTAGTTGGAGCTGGTGGCGT	KRAS	gene-targeting
128	KRAS_2	TCTCGACACAGCAGGTCAAG	KRAS	gene-targeting
129	KRAS_4	AGAGGAGTACAGTGCAATGA	KRAS	gene-targeting
130	NRAS_1	AGAGACCAATACATGAGGAC	NRAS	gene-targeting
131	NRAS_2	CCTTGTTACATCACCACACA	NRAS	gene-targeting
132	NRAS_3	GACTGAGTACAAACTGGTGG	NRAS	gene-targeting
133	HRAS_1	TCTCAGGGAGCAGATCAAAC	HRAS	gene-targeting
134	HRAS_2	ATTCCGTCATCGCTCCTCAG	HRAS	gene-targeting
135	HRAS_3	TGATGGGGAGACGTGCCTGT	HRAS	gene-targeting
136	ARAF_1	AGTGTCCAGGATTTGTCCGG	ARAF	gene-targeting
137	ARAF_2	ATGAGCTCCTCGCCATCCAG	ARAF	gene-targeting
138	ARAF_3	AGAAAGTGGTATGCTCGAGG	ARAF	gene-targeting
139	BRAF_1	ATACCCAATAGAGTCCGAGG	BRAF	gene-targeting
140	BRAF_2	GGCTCTCGGTTATAAGATGG	BRAF	gene-targeting
141	BRAF_3	GCCATCTTATAACCGAGAGC	BRAF	gene-targeting
142	RAF1_1	GCCGAACAAGCAAAGAACAG	RAF1	gene-targeting
143	RAF1_2	CGTGCCAGCACAAAGAGAGC	RAF1	gene-targeting
144	RAF1_3	GACATCGAAATCCATTGAGC	RAF1	gene-targeting
145	TGFBR1_1	TGGCAGAAACACTGTAACGC	TGFBR1	gene-targeting
146	TGFBR1_2	CGATTTGGAGAAGTTTGGAG	TGFBR1	gene-targeting
147	TGFBR1_4	AGAACGTTCGTGGTTCCGTG	TGFBR1	gene-targeting
148	TGFBR2_1	TGTGGAGGTGAGCAATCCCC	TGFBR2	gene-targeting
149	TGFBR2_2	GGGGAAAGGTCGCTTTGCTG	TGFBR2	gene-targeting
150	TGFBR2_3	CAGAGCCATGGAGTAGACAT	TGFBR2	gene-targeting

Signaling2 Library

index	guide	sequence	gene	type
151	TGFBR3_4	AGGAGAGTCTGGAATGAAGA	TGFBR3	gene-targeting
152	TGFBR3_2	ATAGTAGACCACACCATCAA	TGFBR3	gene-targeting
153	TGFBR3_3	GTCTTCAGATGCCACACCAG	TGFBR3	gene-targeting
154	YAP1_1	GCACGATCTGATGCCCGGCG	YAP1	gene-targeting
155	YAP1_2	ATCAGATCGTGCACGTCCGC	YAP1	gene-targeting
156	YAP1_3	ACGAGGTTACCTGTCGGGAG	YAP1	gene-targeting
157	MYC_1	GCCGTATTTCTACTGCGACG	MYC	gene-targeting
158	MYC_2	CTCTGAGACGAGCTTGGCGG	MYC	gene-targeting
159	MYC_3	AGGGTAGGGGAAGACCACCG	MYC	gene-targeting
160	MYCN_4	GCTACAGCCCTGCTTCTACC	MYCN	gene-targeting
161	MYCN_2	CGCGCTTGTTCACGGAAAG	MYCN	gene-targeting
162	MYCN_3	GGGAAAGGGGAAGACCACGG	MYCN	gene-targeting
163	MDM2_1	GTGGTTACAGCACCATCAGT	MDM2	gene-targeting
164	MDM2_4	AGGGTCTCTTGTTCCGAAGC	MDM2	gene-targeting
165	MDM2_3	CACCTCACAGATTCCAGCTT	MDM2	gene-targeting
166	TP53_1	GTTGCAAACCAGACCTCAGG	TP53	gene-targeting
167	TP53_2	GCTTGTAGATGGCCATGGCG	TP53	gene-targeting
168	TP53_3	GTTGATTCCACACCCCCGCC	TP53	gene-targeting
169	BRCA1_1	TCTTGTGCTGACTTACCAGA	BRCA1	gene-targeting
170	BRCA1_2	CAGTTCCAAGGTTAGAGAGT	BRCA1	gene-targeting
171	BRCA1_3	GTTTCTATCATCCAAAGTAT	BRCA1	gene-targeting
172	BRCA2_1	GAGCACAGTAGAACTAAGGG	BRCA2	gene-targeting
173	BRCA2_2	AAGACACGCTGCAACAAAGC	BRCA2	gene-targeting
174	BRCA2_3	CTGTCTACCTGACCAATCGA	BRCA2	gene-targeting
175	PARP1_1	TCCAACAGAAGTACGTGCAA	PARP1	gene-targeting
176	PARP1_2	CTTCCCTGCGAGGAATGCTC	PARP1	gene-targeting
177	PARP1_3	CGAGTCGAGTACGCCAAGAG	PARP1	gene-targeting
178	PARP2_1	ATTGAGGATTGTATTCGGGC	PARP2	gene-targeting
179	PARP2_2	TCAGCGTTCGAATTCCATGG	PARP2	gene-targeting
180	PARP2_3	TCTACGAGTTTTCTTGGCAG	PARP2	gene-targeting
181	CDK12_1	GTCTTTGAGTGAGTAGAAGG	CDK12	gene-targeting
182	CDK12_2	TGGCCTTCAAACTAGACCGA	CDK12	gene-targeting
183	CDK12_4	GTAGGAGTCATAATTGCTCG	CDK12	gene-targeting
184	CHEK1_1	CATAAGGAAAGACCTGTGCG	CHEK1	gene-targeting
185	CHEK1_2	TTTCTGGAGTACTGTAGTGG	CHEK1	gene-targeting
186	CHEK1_3	CTTACTGCAATGCTCGCTGG	CHEK1	gene-targeting
187	ATG4B_1	GTTGGCGAAGGCAAGTCCAT	ATG4B	gene-targeting
188	ATG4B_2	GCGCAAATGGGAGTTGGCGA	ATG4B	gene-targeting
189	ATG4B_4	CGATACGTGGAGCTCCTTGG	ATG4B	gene-targeting
190	CA9_1	CTGCGCAACAATGGCCACAG	CA9	gene-targeting
191	CA9_4	CACTGTGGCCATTGTTGCGC	CA9	gene-targeting
192	CA9_3	CAGAGTCATTGGCGCTATGG	CA9	gene-targeting

Notch Library

index	guide	sequence	gene	type
1	ctl_t_10	GTGGCCATGTGATTCAGGGT	ctl	non-gene-targeting control
2	ctl_t_11	ACCTATCTTAGGCTTTCCCG	ctl	non-gene-targeting control
3	ctl_t_12	TCAGAGAAGACATCCGCCAG	ctl	non-gene-targeting control
4	ctl_t_13	TGGCTGTGGTCCAAGAGTGG	ctl	non-gene-targeting control
5	ctl_t_14	GTTTGGAGGGCAGAACGAAT	ctl	non-gene-targeting control
6	ctl_t_15	CCACAATCCAGATAGGAAGG	ctl	non-gene-targeting control
7	ctl_t_16	TAGCAAAGCCACACCAGCAA	ctl	non-gene-targeting control
8	ctl_t_17	GCTCACTTTGGAGCAACACC	ctl	non-gene-targeting control
9	ctl_t_18	TGTTATGGTTCTCTGCTCAG	ctl	non-gene-targeting control
10	ctl_t_19	CTCTTAGTACACAAGGTCAG	ctl	non-gene-targeting control
11	ctl_t_20	GAGTTCTCCACATAAACCTA	ctl	non-gene-targeting control
12	ctl_t_21	GGGTGGTGAACCTTGCCTGG	ctl	non-gene-targeting control
13	NOTCH1_1	CCTCAGAGCAGTTCACCCCG	NOTCH1	gene-targeting
14	NOTCH1_2	TGTGCACTGTGAGATCAACG	NOTCH1	gene-targeting
15	NOTCH1_3	TGCAGGTCAGTACTGTACCG	NOTCH1	gene-targeting
16	NOTCH1_4	TTGACGTCGATCTCGCATCG	NOTCH1	gene-targeting
17	NOTCH2_1	AACCCTTCAGACACTCACAG	NOTCH2	gene-targeting
18	NOTCH2_2	GGCAGCGGTTCTTCTCACAG	NOTCH2	gene-targeting
19	NOTCH2_3	CCGACTGCAGAGATTCACCA	NOTCH2	gene-targeting
20	NOTCH2_4	CATTGGTGGATACAGATGCG	NOTCH2	gene-targeting
21	NOTCH3_1	GCCACTATGTGAGAACCCCG	NOTCH3	gene-targeting
22	NOTCH3_2	CACAGTAGAAAGAAGCCACG	NOTCH3	gene-targeting
23	NOTCH3_3	TGTAGCCCCCCATATAGCCA	NOTCH3	gene-targeting
24	NOTCH3_4	CACGCTGTGTGATCGCAACG	NOTCH3	gene-targeting
25	NOTCH3_5	ACATGGCGAGGAGTACCCGG	NOTCH3_PEST	gene-targeting
26	NOTCH3_6	AGCCGGAAGTTACCCCCAAG	NOTCH3_PEST	gene-targeting
27	NOTCH3_7	AATCGAGGGGCACAGCCACA	NOTCH3_PEST	gene-targeting
28	NOTCH3_8	GGGGAGTCCAGCGAGTCCAC	NOTCH3_PEST	gene-targeting
29	NOTCH4_1	GGTACCCCCATTGAAGCACG	NOTCH4	gene-targeting
30	NOTCH4_2	AGCTCCCTGCCTGAACCACG	NOTCH4	gene-targeting
31	NOTCH4_3	ACACCCCTCCATTAACACAT	NOTCH4	gene-targeting
32	NOTCH4_4	TGTGTCAGCCTGGCTATTCG	NOTCH4	gene-targeting
33	DLL1_1	AGTGTTCGTCACACACGAAG	DLL1	gene-targeting
34	DLL1_2	GGTAGTGCTTGAGGCACACG	DLL1	gene-targeting
35	DLL1_3	GCCCCCTTACCATTAGAACA	DLL1	gene-targeting
36	DLL1_4	CCGGCCAGGTGAAGCCGAAG	DLL1	gene-targeting
37	DLL4_1	CTGCCTTAAGCACTTCCAGG	DLL4	gene-targeting
38	DLL4_2	AAGAAGCGCAATGACCACTT	DLL4	gene-targeting
39	DLL4_3	TCGGGCTGTCATGAACAGAA	DLL4	gene-targeting
40	DLL4_4	CAGCACGCACCGTTGGCACA	DLL4	gene-targeting
41	DTX1_1	GTGTGGGAGTGGGAGAACGA	DTX1	gene-targeting
42	DTX1_2	GTGCTGAAGGAGGACGCTCG	DTX1	gene-targeting
43	DTX1_3	GGTTCGAAGATACATGCAGA	DTX1	gene-targeting
44	DTX1_4	CCACGACTGCGACTTAGGGA	DTX1	gene-targeting

Notch Library

index	guide	sequence	gene	type
45	JAG1_1	CTATTTCTGCAAGTGCCCCG	JAG1	gene-targeting
46	JAG1_2	GCGCAGCGATGCGTTCCCCA	JAG1	gene-targeting
47	JAG1_3	ATGGGCCCCGAATGTAACAG	JAG1	gene-targeting
48	JAG1_4	TCATCAGCCGTGTCTCAACG	JAG1	gene-targeting
49	JAG2_1	TGAAGTGCAGGCTCTTCCAG	JAG2	gene-targeting
50	JAG2_2	GCTCGCTGCTATAACCTGGA	JAG2	gene-targeting
51	JAG2_3	GTGTGCTTCGAACCCGTGTG	JAG2	gene-targeting
52	JAG2_4	CGTACGTGTCGCACTCGTCG	JAG2	gene-targeting
53	ADAM10_1	GGATTCATCCAGACTCGTGG	ADAM10	gene-targeting
54	ADAM10_2	CATAAATACGGTCCTCAGGG	ADAM10	gene-targeting
55	ADAM10_3	GGAAATGGAATGGTAGAACA	ADAM10	gene-targeting
56	ADAM10_4	TTCCATCAATAACAGACCCA	ADAM10	gene-targeting
57	ADAM17_1	GTAGACAGAGAACCACCTGA	ADAM17	gene-targeting
58	ADAM17_2	TGGTGAAAAGCACTACAACA	ADAM17	gene-targeting
59	ADAM17_3	CATCGCTTCTACAGATACAT	ADAM17	gene-targeting
60	ADAM17_4	ACAAAATTTCAAGGTCGTGG	ADAM17	gene-targeting
61	APH1A_7	AGTGATCAAGAAAAGCGCGA	APH1A	gene-targeting
62	APH1A_8	GCGTTATCATCCTGGTCGCA	APH1A	gene-targeting
63	APH1A_6	GACACCACTGATGATACCGA	APH1A	gene-targeting
64	APH1A_9	GCTCACCATAGGCCATCTGG	APH1A	gene-targeting
65	PSEN1_1	CCACCATGCAGAGAGTCACA	PSEN1	gene-targeting
66	PSEN1_2	ATTTATACAGAACCACCAGG	PSEN1	gene-targeting
67	PSEN1_3	GCCACGCAGTCCATTCAGGG	PSEN1	gene-targeting
68	PSEN1_4	ACCTGCCGGGAGTTACCCTG	PSEN1	gene-targeting
69	PSEN2_1	TCTGTAGTGGGGTTCCCGGG	PSEN2	gene-targeting
70	PSEN2_2	GCTGACTGTCTGGAACTTCG	PSEN2	gene-targeting
71	PSEN2_3	GAGGTACTTGATGAACACTA	PSEN2	gene-targeting
72	PSEN2_4	ACTGAGGACACACCCTCGGT	PSEN2	gene-targeting
73	NCSTN_1	TCTCCAGCAGAACCATGTAA	NCSTN	gene-targeting
74	NCSTN_2	CTCACTGCAGAGAAATACAG	NCSTN	gene-targeting
75	NCSTN_3	ATGGTCTACGATATGGAGAA	NCSTN	gene-targeting
76	NCSTN_4	CAGTGCCCAAATGATGGGTT	NCSTN	gene-targeting
77	PSENEN_1	CATCTTCTGGTTCTTCCGAG	PSENEN	gene-targeting
78	PSENEN_2	TGACCAACCAGAGAAAAGGC	PSENEN	gene-targeting
79	PSENEN_3	CTGTGCCGGAAGTACTACCT	PSENEN	gene-targeting
80	PSENEN_4	TGAGCACTATCACCCAGAAG	PSENEN	gene-targeting
81	POFUT1_1	GGGAGGCCTTACCCATGCAG	POFUT1	gene-targeting
82	POFUT1_2	GCTGGTACTCAATCCAAGGA	POFUT1	gene-targeting
83	POFUT1_3	AGCCCAGTTCCCCGTCCTAG	POFUT1	gene-targeting
84	POFUT1_4	CCTCCAAGCTGATGACCCGA	POFUT1	gene-targeting
85	HES1_1	GCACAGACCCAAGTGTGCTG	HES1	gene-targeting
86	HES1_2	GAAAAATTCCTCGTCCCCGG	HES1	gene-targeting
87	HES1_3	CCTACTCTATGCAGAGCTCG	HES1	gene-targeting
88	HES1_4	TCAATGCCATGACCTACCCC	HES1	gene-targeting

Notch Library

index	guide	sequence	gene	type
89	HES5_1	TGCTGGAGCAGGAGTTCGCG	HES5	gene-targeting
90	HES5_2	ACTACAGCGAAGGCTACTCG	HES5	gene-targeting
91	HES5_3	AGCTACCTGAAGCACAGCAA	HES5	gene-targeting
92	HES5_4	CGGCGCATCTTCTCCACCAC	HES5	gene-targeting
93	HEY1_1	TCAACAACTACGCTTCCCAG	HEY1	gene-targeting
94	HEY1_2	TGTCCGAAGACGGTCCCCCA	HEY1	gene-targeting
95	HEY1_3	ACTCCGATAGTCCATAGCAA	HEY1	gene-targeting
96	HEY1_4	TGTTATTGATCCGGTCTCGT	HEY1	gene-targeting
97	HEY2_1	CATGGACGAGACCATCGACG	HEY2	gene-targeting
98	HEY2_2	AGTGCCTAACAGAAGTTGCG	HEY2	gene-targeting
99	HEY2_3	GGGTTGACTCTGAGGCATGG	HEY2	gene-targeting
100	HEY2_4	AAGATGCTTCAGGCAACAGG	HEY2	gene-targeting
101	MAML1_1	GCATAAGAAGACTCGCCGGG	MAML1	gene-targeting
102	MAML1_2	GTTAGGCTCTCCACAAGTGA	MAML1	gene-targeting
103	MAML1_3	AGAACTCCGCGAATAACCAG	MAML1	gene-targeting
104	MAML1_4	GGAGTGGAAGGAGCTCATCG	MAML1	gene-targeting
105	MAML2_1	AGAGTGTTAGTCTTTCGCAG	MAML2	gene-targeting
106	MAML2_2	ATGTCTGCTGACCAAAAGAA	MAML2	gene-targeting
107	MAML2_3	GGTGGCCTCCAAATAAACAA	MAML2	gene-targeting
108	MAML2_4	AGTTCAAGGTCACTCATGGG	MAML2	gene-targeting
109	NCOR2_1	TGATACTGCCGGGTGTCGGA	NCOR2	gene-targeting
110	NCOR2_2	GCTCTGGGCTCAGTTCCGGG	NCOR2	gene-targeting
111	NCOR2_3	GCTGCTGCAAGATCTCATCG	NCOR2	gene-targeting
112	NCOR2_4	TGGTGATCTCTCGGTCCACG	NCOR2	gene-targeting
113	FBXW7_1	CGAACTGGAGGCTCTCTGAG	FBXW7	gene-targeting
114	FBXW7_2	AAGAGCGGACCTCAGAACCA	FBXW7	gene-targeting
115	FBXW7_3	ACATTAGTGGGACATACAGG	FBXW7	gene-targeting
116	FBXW7_4	GTTGGAGTAGAACCTAGACC	FBXW7	gene-targeting
117	NUMB_1	GATGAAGAAGGCGTTCGCAC	NUMB	gene-targeting
118	NUMB_2	AGTTGCCTTCCACTATGCAG	NUMB	gene-targeting
119	NUMB_3	GGCCACCTTACCCGAACATG	NUMB	gene-targeting
120	NUMB_4	CTATCGTCTGGTCAACTATG	NUMB	gene-targeting
121	PLK1_7	GCCCCAGCACATCAACCCCG	PLK1	gene-targeting
122	PLK1_8	CGTTGTCCTCGAAAAAGCCG	PLK1	gene-targeting
123	PLK1_4	AACCAAAGTCGAATATGACG	PLK1	gene-targeting
124	PLK1_5	AGCCAAGCACAATTTGCCGT	PLK1	gene-targeting
125	PLK4_7	TCATGCACCAGATCATCACA	PLK4	gene-targeting
126	PLK4_8	TTGACTGTGTCAGTGTCGAA	PLK4	gene-targeting
127	PLK4_9	CGATTCTGATAACCCCATGG	PLK4	gene-targeting
128	PLK4_5	CAGAGGAATAAGCTCTACGA	PLK4	gene-targeting

Table S2 Experimental information for *in vivo* pooled CRISPR screens in 181 PDX tumours. Columns show patient ID, tumour ID (dataset), PDX passage number used as input material for transduction, sgRNA library, viral dose (infectious units x 10^6), site (subcutaneous or mammary fat pad), number of days *in vivo* and drug (if administered).

patient	tumour	passage	library	IU	site	days	drug
C1368	C1368-1211231	6	Notch	8	SQ	31	
C1368	C1368-1211232	6	Notch	8	SQ	27	
C1368	C1368-1211233	6	Notch	8	SQ	23	
C1368	C1368-1211234	6	Notch	8	SQ	34	
C2271	C2271-121232222111	12	Notch	8	SQ	23	
C2271	C2271-121232222112	12	Notch	8	SQ	29	
C2271	C2271-121232222113	12	Notch	8	SQ	29	
C2271	C2271-121232222114	12	Notch	8	SQ	26	
C0331	C0331-2234311	6	Notch	8.6	SQ	35	
C0331	C0113-2234312	6	Notch	8.6	SQ	35	
C0331	C0331-2234313	6	Notch	8.6	SQ	35	
C0331	C0331-2234314	6	Notch	8.6	SQ	35	
C0331	C0331-2231	3	Signaling1	20.3	SQ	28	
C0331	C0331-2232	3	Signaling1	20.3	SQ	28	
C0331	C0331-2233	3	Signaling1	20.3	SQ	28	
C0331	C0331-231	2	Signaling1	24.6	SQ	34	
C0331	C0331-232	2	Signaling1	24.6	SQ	40	
C0592	C0592-221	2	Signaling1	8.3	SQ	56	
C0592	C0592-222	2	Signaling1	8.3	SQ	56	
C0592	C0592-223	2	Signaling1	2.8	SQ	64	
C0592	C0592-224	2	Signaling1	2.8	SQ	56	
C0592	C0592-225	2	Signaling1	2.8	SQ	64	
C1368	C1368-12111211	7	Signaling1	7.7	SQ	30	
C1368	C1368-12111212	7	Signaling1	7.7	SQ	29	
C1368	C1368-121112431131	11	Signaling1	10	MFP	36	
C1368	C1368-121112431132	11	Signaling1	10	SQ	30	
C1368	C1368-121112431133	11	Signaling1	10	MFP	35	
C1368	C1368-12111243131	10	Signaling1	20	SQ	15	
C1368	C1368-12111243132	10	Signaling1	20	SQ	22	
C1368	C1368-12111243133	10	Signaling1	20	SQ	22	
C1368	C1368-151	2	Signaling1	18.7	SQ	28	
C1368	C1368-152	2	Signaling1	18.7	SQ	28	
C1368	C1368-153	2	Signaling1	18.7	SQ	56	
C1368	C1368-154	2	Signaling1	18.7	SQ	28	
C1373	C1373-23121524111	10	Signaling1	7	SQ	34	
C1373	C1373-23121524112	10	Signaling1	7	SQ	40	
C1373	C1373-2312152413411	12	Signaling1	8.7	SQ	59	
C1373	C1373-2312152413412	12	Signaling1	8.7	SQ	46	
C1373	C1373-2312152413413	12	Signaling1	8.7	SQ	52	
C1373	C1373-2351	3	Signaling1	8.7	SQ	83	
C1373	C1373-2352	3	Signaling1	8.7	SQ	56	

patient	tumour	passage	library	IU	site	days	drug
C1379	C1379-121D1323111	10	Signaling1	8.8	SQ	33	
C1379	C1379-121D1323112	10	Signaling1	8.8	SQ	33	
C1379	C1379-121D1323114	10	Signaling1	8.8	SQ	33	
C1392	C1392-124624341	8	Signaling1	19.3	SQ	50	
C1392	C1392-124624342	8	Signaling1	19.3	SQ	39	
C1392	C1392-124624343	8	Signaling1	19.3	SQ	25	
C1392	C1392-1246245	6	Signaling1	7.7	SQ	41	
C2191	C2191-4222341	6	Signaling1	9	SQ	30	
C2191	C2191-4222342	6	Signaling1	9	SQ	23	
C2191	C2191-4222343	6	Signaling1	9	SQ	29	
C2191	C2191-4222344	6	Signaling1	9	SQ	30	
C2191	C2191-4251	3	Signaling1	9.4	SQ	79	
C2191	C2191-4252	3	Signaling1	9.4	SQ	50	
C2191	C2191-4253	3	Signaling1	9.4	SQ	50	
C2191	C2191-4254	3	Signaling1	9.4	SQ	50	
C2271	C2271-11314A681133311	14	Signaling1	8	SQ	70	
C2271	C2271-11314A681133312	14	Signaling1	8	SQ	49	
C2271	C2271-11314A681133313	14	Signaling1	8	SQ	14	
C2271	C2271-11314A681133314	14	Signaling1	8	SQ	21	
C2271	C2271-11314A681133331	14	Signaling1	9	SQ	17	
C2271	C2271-11314A681133332	14	Signaling1	9	SQ	35	
C2271	C2271-11314A681133333	14	Signaling1	9	SQ	42	
C2271	C2271-11314A681133341	14	Signaling1	8	SQ	42	
C2271	C2271-11314A681133342	14	Signaling1	8	SQ	21	
C2271	C2271-11314A681133343	14	Signaling1	8	SQ	28	
C2271	C2271-11314A681133344	14	Signaling1	8	SQ	35	
C2271	C2271-11314A681141	11	Signaling1	7.2	SQ	8	
C2271	C2271-11314A681142	11	Signaling1	7.2	SQ	8	
C2271	C2271-11314A6841421	12	Signaling1	20	SQ	30	
C2271	C2271-11314A6841422	12	Signaling1	10	SQ	31	
C2271	C2271-11314A6841423	12	Signaling1	5	SQ	31	
C2271	C2271-11314A6841433	12	Signaling1	20	SQ	28	
C2271	C2271-11314A6841434	12	Signaling1	20	SQ	30	
C2271	C2271-11314A6841443	12	Signaling1	9.4	SQ	34	
C2271	C2271-11314A6841444	12	Signaling1	9.4	SQ	62	
C2271	C2271-1151	3	Signaling1	20	MFP	50	
C2271	C2271-1152	3	Signaling1	20	MFP	42	
C2271	C2271-1153	3	Signaling1	20	SQ	43	
C2271	C2271-1161	3	Signaling1	20	SQ	24	
C2271	C2271-1162	3	Signaling1	20	SQ	23	
C2271	C2271-1163	3	Signaling1	20	SQ	24	
C2271	C2271-1212322211	9	Signaling1	9.2	SQ	30	
C2271	C2271-1212322215	9	Signaling1	9.2	SQ	30	
C2271	C2271-1212322216	9	Signaling1	9.2	SQ	30	
C2553	C2553-1112112111321	12	Signaling1	9.4	SQ	37	idasanutlin
C2553	C2553-1112112111322	12	Signaling1	9.4	SQ	37	vehicle

patient	tumour	passage	library	IU	site	days	drug
C2553	C2553-1112112111323	12	Signaling1	9.4	SQ	37	idasanutlin
C2553	C2553-111211211140	11	Signaling1	8	SQ	48	
C2553	C2553-111211211142	11	Signaling1	8	SQ	48	
C2553	C2553-111211211147	11	Signaling1	8	SQ	60	
C2553	C2553-111211211149	11	Signaling1	8	SQ	65	
C2553	C2553-221	2	Signaling1	20.3	SQ	87	
C2553	C2553-222	2	Signaling1	20.3	SQ	87	
C2553	C2553-223	2	Signaling1	20.3	SQ	111	
C2657	C2657-31332111	7	Signaling1	9.4	SQ	28	
C2657	C2657-31332112	7	Signaling1	9.4	SQ	25	
C2657	C2657-31332113	7	Signaling1	9.4	SQ	25	
C2657	C2657-31332114	7	Signaling1	9.4	SQ	21	
C2657	C2657-3171	3	Signaling1	20	SQ	43	
C2657	C2657-3172	3	Signaling1	10	SQ	41	
C2657	C2657-3173	3	Signaling1	5	SQ	41	
C3037	C3037-141431	5	Signaling1	10.6	SQ	62	
C3037	C3037-141432	5	Signaling1	10.6	SQ	39	
C3037	C3037-141433	5	Signaling1	10.6	SQ	39	
C3037	C3037-141434	5	Signaling1	10.6	SQ	45	
C0331	C0331-2235221	6	Signaling2	8.2	SQ	33	
C0331	C0331-2235226	6	Signaling2	8.2	SQ	27	
C0331	C0331-22371	4	Signaling2	7.5	SQ	46	
C0331	C0331-22372	4	Signaling2	7.5	MFP	35	
C0331	C0331-22373	4	Signaling2	7.5	MFP	31	
C0331	C0331-22374	4	Signaling2	7.5	SQ	38	
C0468	C0468-241	2	Signaling2	7.5	SQ	58	
C0468	C0468-242	2	Signaling2	7.5	SQ	58	
C1368	C1368-15820	4	Signaling2	7.5	SQ	28	
C1368	C1368-15821	4	Signaling2	7.5	SQ	19	
C1368	C1368-15822	4	Signaling2	7.5	SQ	23	
C1368	C1368-15823	4	Signaling2	7.5	SQ	35	
C1368	C1368-15824	4	Signaling2	7.5	SQ	43	
C1368	C1368-15825	4	Signaling2	7.5	SQ	19	
C1368	C1368-15826	4	Signaling2	7.5	SQ	23	
C1368	C1368-15827	4	Signaling2	7.5	SQ	35	
C1368	C1368-15828	4	Signaling2	7.5	SQ	40	
C1368	C1368-15829	4	Signaling2	7.5	SQ	28	
C1368	C1368-1582A	4	Signaling2	7.5	SQ	42	
C1368	C1368-1582B	4	Signaling2	7.5	SQ	42	
C1375	C1375-41413411	7	Signaling2	7.8	SQ	79	
C1375	C1375-41413412	7	Signaling2	7.8	SQ	62	
C1375	C1375-41413413	7	Signaling2	7.8	SQ	97	
C1379	C1379-121D132312331	12	Signaling2	7.5	SQ	41	
C1379	C1379-121D132312332	12	Signaling2	7.5	SQ	38	
C1379	C1379-121D132312333	12	Signaling2	7.5	SQ	27	
C1379	C1379-121D132312334	12	Signaling2	7.5	SQ	27	

patient	tumour	passage	library	IU	site	days	drug
C1379	C1379-171	2	Signaling2	8.8	SQ	70	
C1379	C1379-172	2	Signaling2	8.8	SQ	70	
C1379	C1379-173	2	Signaling2	8.8	SQ	70	
C1379	C1379-174	2	Signaling2	8.8	SQ	70	
C1383	C1383-21	1	Signaling2	8	SQ	41	
C1383	C1383-22	1	Signaling2	8	SQ	52	
C1383	C1383-23	1	Signaling2	8	SQ	41	
C1471	C1471-11	1	Signaling2	8	SQ	121	
C1471	C1471-12	1	Signaling2	8	SQ	139	
C1471	C1471-13	1	Signaling2	8	SQ	56	
C1471	C1471-14	1	Signaling2	8	SQ	56	
C1491	C1491-21	1	Signaling2	8	SQ	73	
C1491	C1491-22	1	Signaling2	8	SQ	71	
C1491	C1491-23	1	Signaling2	8	SQ	69	
C1491	C1491-24	1	Signaling2	8	SQ	73	
C1557	C1557-21	1	Signaling2	9.2	SQ	71	
C1557	C1557-22	1	Signaling2	9.2	SQ	68	
C2191	C2191-42223331	7	Signaling2	8	MFP	52	
C2191	C2191-42223332	7	Signaling2	8	MFP	59	
C2191	C2191-42223333	7	Signaling2	8	SQ	73	
C2191	C2191-42223334	7	Signaling2	8	SQ	59	
C2271	C2271-12141	4	Signaling2	8	MFP	33	
C2271	C2271-12142	4	Signaling2	8	MFP	22	
C2271	C2271-12143	4	Signaling2	8	SQ	27	
C2271	C2271-12144	4	Signaling2	8	SQ	29	
C2438	C2438-1211	3	Signaling2	7.5	SQ	227	
C2438	C2438-1212	3	Signaling2	7.5	SQ	175	
C2438	C2438-1213	3	Signaling2	7.5	SQ	161	
C2438	C2438-1214	3	Signaling2	7.5	SQ	141	
C2553	C2553-2131	3	Signaling2	8	MFP	45	
C2553	C2553-2132	3	Signaling2	8	MFP	45	
C2553	C2553-2133	3	Signaling2	8	SQ	45	
C2553	C2553-2134	3	Signaling2	8	SQ	45	
C2591	C2591-521	2	Signaling2	12.8	SQ	138	
C2591	C2591-522	2	Signaling2	12.8	SQ	138	
C2591	C2591-531	2	Signaling2	11.2	SQ	125	
C2591	C2591-533	2	Signaling2	11.2	SQ	95	
C2591	C2591-612	2	Signaling2	7.5	SQ	154	
C3037	C3037-1414211	6	Signaling2	9	MFP	38	
C3037	C3037-1414212	6	Signaling2	9	MFP	59	
C3037	C3037-1414213	6	Signaling2	9	SQ	38	
C3037	C3037-1414214	6	Signaling2	9	SQ	50	
C3278	C3278-11	1	Signaling2	7.5	SQ	28	
C3278	C3278-12	1	Signaling2	7.5	SQ	28	
C3278	C3278-13	1	Signaling2	7.5	SQ	28	
C3278	C3278-14	1	Signaling2	7.5	SQ	28	

patient	tumour	passage	library	IU	site	days	drug
C3466	C3466-32	1	Signaling2	8	MFP	76	
C3466	C3466-33	1	Signaling2	8	MFP	49	

Supplementary Methods: Inference of fitness informed by barcode distributions

1.1 Mathematical notation Refer to **Figure S11** for a quick reference on the mathematical notation. Circles represent individual cells while colours encode bar codes. We introduce notation for two types of measurements. On the left of **Figure S11**, we show the "final condition measurements" where sequencing is performed after several weeks of time *in vivo* in the PDX model. On the right, we show "initial condition measurements" corresponding to the sequenced barcoded sgRNA library plasmid that was packaged into lentiviruses used to transduce the PDX cells that initiated the transplant. The role of the initial condition measurements is to factor out differences in clone sizes due to technical effects such as differences in packaging or transduction efficiencies between sgRNAs.

1.1.1 Types; targeting and non-targeting sgRNA

Let $t \in \mathscr{T}$ denote an index for sgRNA libraries, shown as rows in **Figure S11**. For example, in the experiments shown in the main paper using breast PDX models, t takes values in a set $\mathscr{T}$ of size 192, structured into 168 sgRNAs targeting 56 genes in triplicate plus 24 non-targeting control sgRNAs. Since we will define fitness relative to a reference set of sgRNAs, the non-targeting control sgRNAs can be used to estimate a "reference fitness," although we discuss other normalization strategies in Section 1.8. Collectively, we refer to the set $\mathscr{T}$ indexing both targeting and non-targeting sgRNAs as the set of sgRNA *types*.

1.1.2 Final condition measurements

Since the targeted sequencing amplicon contains both the sgRNA and barcode libraries, we assume there is a known mapping from barcode (colours in **Figure S11**) to corresponding sgRNA index t as described in Methods (Extraction of sgRNA and UMI sequences). Let I_t denote the number of distinct barcodes of type t, each corresponding to an individual cancer cell transduced in the PDX model. The variable I_t is not observed.

As the barcode is replicated during cell division, clonal expansion gives rise to one birth-death process for each barcode. We now introduce notation tracking the dynamics of those $I = \sum_t I_t$ distinguisable birth-death processes. After a fixed time period τ—several weeks *in vivo* —, we take a snapshot of this population of branching processes. Each of the branching processes will be either extinct or have a number of progenies. Let D_t^{*j} denote the number of cells at time τ containing the j-th barcode associated with the t-th sgRNA type. Extinction of one of the barcode lineages corresponds to the event $D_t^{*j} = 0$ for some j and t. The population at time τ is sampled by targeted sequencing of the tumour extracted from the mouse. Let D_t^j denote the number of reads matched to the j-th barcode associated with the t-th sgRNA type. The sequencing and extraction of counts, accounting for sequence errors in the barcodes is detailed in Methods (Extraction of sgRNA and UMI sequences).

The random variables D_t^j are observed in a censored fashion: only those taking value greater or equal to one are measured. We let S_t denote the number of distinct barcodes of type t such that the corresponding

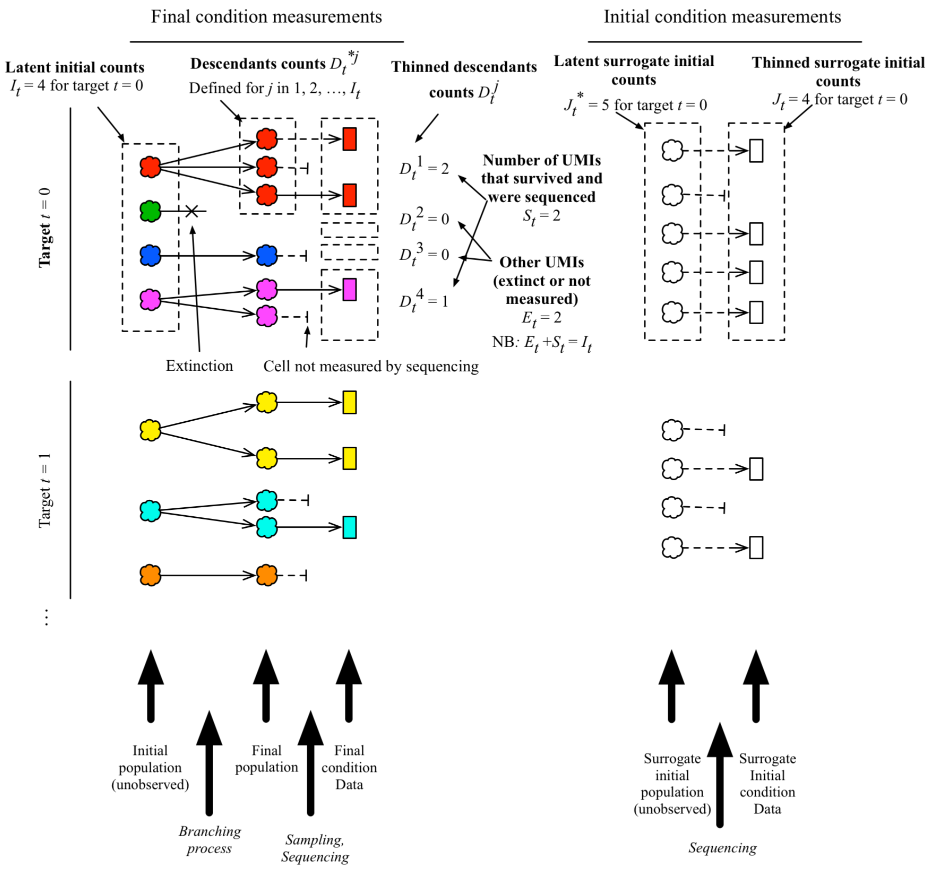

Figure S 11 Notation used for defining the fitness model and associated inference methods.

birth-death process has at least one survivor at time τ that was sequenced, i.e.,

$$S_t = \sum_{j=1}^{I_t} \mathbf{1}[D_t^j > 0],$$

where $\mathbf{1}[\ldots]$ denotes the indicator function, defined for a boolean expression b by

$$\mathbf{1}[b] = \begin{cases} 1 & \text{if } b \text{ is true} \\ 0 & \text{otherwise.} \end{cases}$$

Similarly, the number of distinct barcodes of type t with no sequenced descendants is denoted by E_t. By construction, $I_t = E_t + S_t$, that is the total number of barcodes for a target is the sum of the observed and unobserved barcodes.

1.1.3 Initial condition measurements

We now introduce notation for the initial condition measurement (see **Figure S11**, right). As in the final condition measurements, the initial condition measurements segregate a collection of cells into $|\mathscr{T}|$ types. Let J_t^* denote the number of cells of type $t \in \mathscr{T}$. After performing targeted sequencing, we observe J_t reads of type t.

1.1.4 Experimental replicates and conditions

For simplicity, we have shown the notation in this section based on a single replicate and a single experimental condition. When several replicates and/or experimental conditions are involved, all the variables involved in final condition measurements gain an additional subscript, $e \in \mathscr{E}$, becoming $I_{t,e}$, $E_{t,e}$, $S_{t,e}$, $D_{t,e}^{*j}$, and $D_{t,e}^j$. We will omit the experimental subscript e when describing methods such as the ratio estimator which consider a single set of final measurements at a time.

1.2 Summary statistics

Diversity of datasets was assessed using the following metrics:

Average UMIs per guide:

$$\overline{S} = \frac{1}{|\mathscr{T}|} \sum_{t \in \mathscr{T}} S_t$$

Shannon Diversity Index: Let

$$
\begin{aligned}
D &= \sum_{t=1}^{|\mathscr{T}|} \sum_{j=1}^{I_t} D_t^j, \qquad (1) \\
p_t &= \frac{1}{D} \sum_{j=1}^{I_t} D_t^j,
\end{aligned}
$$

then the Shannon Diversity Index (SDI) H is defined as follows

$$
H = -\sum_{t=1}^{|\mathscr{T}|} p_t \log p_t.
$$

Area under Lorenz curve: (or alternatively gini coefficient = 1 - 2 x AUC): Define p_t as for the SDI. Let $p_{(t)}$ be the order statistics for $\{p_t\}_{t=1}^{|\mathscr{T}|}$ i.e. sorted ordering from smallest to largest. Define:

$$
\begin{aligned}
X_i &= \frac{i}{|\mathscr{T}|}, \\
Y_i &= \sum_{j=1}^{i} p_{(j)}.
\end{aligned}
$$

The Lorenz curve is the continuous piecewise linear curve defined by the set of points (X_i, Y_i). The area under the curve is computed as the sum of trapezoidal areas.

1.3 Definition of fitness Each random variable D_t^{*j} can be conceptualized as the marginal distribution of a stochastic process observed at time τ. One popular class of approaches to define fitness in this context is to select a parametric family of stochastic processes, for example a time homogeneous birth-death process, and to define fitness in terms of the inferred stochastic process parameters [1]. However in the context of the experimental setup considered here, where repeated measurements of the same realization of a stochastic process is not available, and where time-homogeneity is unrealistic due to initial population bottleneck effect due to engraftment, we opt for an alternative method based on direct modelling of marginal distributions.

The approach we propose is based on the following assumptions.

Assumption 1. *[Exchangeability.] We assume for each type $t \in \mathscr{T}$, that there is an infinite exchangeable collection $\{D_t^{*j} : j \in \{0, 1, 2, \dots\}\}$, from which the set which we seek to model is defined as the following set of random finite size: $\{D_t^{*j} : j \in \{0, 1, 2, \dots, I_t\}\}$.*

This mathematical assumption encodes the hypothesis that the barcodes have a negligible effect on fitness. The assumption implies that the random variables $D_t^{*1}, D_t^{*2}, \dots$ are identically distributed and we denote the common marginal by D_t^*.

We seek an approach to compare the fitness of any two types, without loss of generality $t = 0$ and $t = 1$. One approach is to compare the expectations $\mathbb{E}_\tau[f(D_0^*)]$ and $\mathbb{E}_\tau[f(D_1^*)]$ for a suitable function f, for

example the simple mean $f(x) = x$, or a truncated version when the distribution on D_t^* is modelled using heavy tail distributions (see Section 1.3.1 for details). Here we add a subscript τ when we wish to reinforce that the distribution of the random variables D_t^* depend on the in vivo time period τ. We denote the growth model by $k_{t,f}(\tau) = \mathbb{E}_\tau[f(D_t^*)]$. To ensure that ordinal quantification of fitness is not sensitive on τ, we make the following assumption on the growth model:

Assumption 2. *[Rank-preserving growth model.] We assume that for any τ, τ', t, t', we have $k_{t,f}(\tau) < k_{t',f}(\tau)$ implies $k_{t,f}(\tau') < k_{t',f}(\tau')$.*

We emphasize that this assumption should be viewed as an approximation: there are several phenomena that could cause violation of this assumption in real biological systems, for example if the environment change over time [2].

We also assume that the processes of sequencing and sampling cells during final condition measurements are not preferential with respect to the types t, i.e. that

$$\frac{\mathbb{E}[f(D_1^*)]}{\mathbb{E}[f(D_0^*)]} \approx \frac{\mathbb{E}[f(D_1)]}{\mathbb{E}[f(D_0)]} =: R. \tag{2}$$

For example, if $f(x) = x$ and D_t is obtained by Poisson thinning of D_t^*, then the above holds exactly. Based on Equation (2), in the following we can analyze the random variables D_t instead of D_t^*.

1.3.1 Choice of test function

We make two technical assumptions:

Assumption 3. *The test function f is such that:*

1. *it is zero at zero, $f(0) = 0$;*
2. *for all $t \in \mathscr{T}$, the random variable $|f(D_t)|$ is integrable, $\mathbb{E}|f(D_t)| < \infty$.*

The first assumption is used when analyzing the effect of the censoring of the clones (clones with clone size of zero are not observed in the final conditions), while the second is used to invoke the law of large number when deriving frequentist estimators. This assumption is satisfied for example with the identity function $f(x) = x$ when D_t has light tails. However, in order to perform model selection over classes of models containing heavy tail distributions (such as the Yule and Beta-Negative-Binomial families [3]), we also consider a conditional-Winsorized mean, defined as $f(d) = \mathbf{1}[d < d^*]d + \mathbf{1}[d \geq d^*]d^*$, where $d^* = \inf\{d : 98\% \leq \mathbb{P}(D \leq d | D > 0)\}$.

1.4 Frequentist approach As a computationally inexpensive baseline, we start with a frequentist approach to estimating the fitness along with confidence intervals. The method is based on a ratio estimator combined with confidence intervals built using a delta method asymptotic analysis. In the following we consider the variables I_t, J_t^* as deterministic, and we strengthen Assumption 1 into independence and identical distribution.

Before describing the ratio estimator, consider first the naive Monte Carlo estimate,

$$\mathbb{E}[f(D_t)] \approx \frac{\sum_{j=1}^{I_t} f(D_t^j)}{I_t},$$

where $t \in \{0, 1\}$ and $D_t^1, D_t^2, \ldots, D_t^{I_i}$ are I_t clone sizes. The motivation for using more complicated estimator is that the naive Monte Carlo estimate cannot be computed from the observed data. Since we do not observed the D_i^j's that are equal to zero, the value of the denominator I_t is unknown. To make progress, we give up on computing $\mathbb{E}[f(D_0)]$ and $\mathbb{E}[f(D_1)]$ separately and instead compute $\mathbb{E}[f(D_1)]/\mathbb{E}[f(D_0)]$ while at the same time adding another source of measurement. We can think of $t = 0$ as a control sgRNA and $t = 1$ as the targeting sgRNA of interest.

The additional source of measurement consists in an estimate for the initial conditions, i.e. of the initial proportions of barcodes associated to type $t = 0$ versus $t = 1$. To simplify exposition, let us first consider the hypothetical case where there is no sampling error involved in the initial condition measurement. We measure an initial count for each sgRNA, J_0^* and J_1^*, with the assumed property that:

Assumption 4. *There is a positive constant λ such that $I_0 \approx \lambda J_0^*$ and $I_1 \approx \lambda J_1^*$.*

The ratio estimator does not require knowing or estimating the numerical value of λ. We rewrite the ratio of expectations as follows:

$$\begin{aligned} R = R_{1,0} := \frac{\mathbb{E}[f(D_1)]}{\mathbb{E}[f(D_0)]} &= \frac{\mathbb{E}[f(D_1)\mathbf{1}[D_1 > 0]]}{\mathbb{E}[f(D_0)\mathbf{1}[D_0 > 0]]} \text{ (from Assumption 3.1)} \\ &= \frac{\mathbb{E}[f(D_1)|D_1 > 0]}{\mathbb{E}[f(D_0)|D_0 > 0]} \frac{\mathbb{P}(D_1 > 0)}{\mathbb{P}(D_0 > 0)} \\ &=: \frac{F_1}{F_0} \frac{P_1}{P_0}. \end{aligned}$$

We now show how to estimate each of F_0, F_1 and P_1/P_0. For F_t, simply take average among the surviving clones:

$$\hat{F}_t := \frac{\sum_{j:D_t^j>0} f(D_t^j)}{S_t} = \frac{\sum f(D_t^j)}{S_t} \to F_t, \tag{3}$$

almost surely as $S_t \to \infty$,[1] based on Assumptions 3.1 and 3.2. For P_1/P_0, we use the additional source of data J_t:

$$\begin{aligned} \frac{S_1}{J_1^*} \frac{J_0^*}{S_0} &= \frac{S_1}{\lambda J_1^*} \frac{\lambda J_0^*}{S_0} \\ &\approx \frac{S_1}{I_1} \frac{I_0}{S_0} \\ &\to \frac{P_1}{P_0}, \end{aligned} \tag{4}$$

[1]To be more precise, let us introduce an asymptotic parameter n which can be interpreted as a sequencing effort. Let S_t be a function of n, $S_t(n)$, such that $S_t(n) \to \infty$ as $n \to \infty$. Based on this notation, from now on, all convergence statements are with respect to $n \to \infty$.

almost surely. Combining Equation (3) and (4), we obtain the ratio estimator:

$$\begin{aligned}\hat{R} &:= \frac{\sum_{j:D_0^j>0} f(D_0^j)}{J_0^*} \frac{J_1^*}{\sum_{j:D_1^j>0} f(D_1^j)} \\ &= \frac{\frac{1}{S_0}\sum_{j:D_0^j>0} f(D_0^j)}{\frac{1}{S_1}\sum_{j:D_1^j>0} f(D_1^j)} \frac{S_0}{J_0^*} \frac{J_1^*}{S_1} \\ &\to R,\end{aligned}$$

almost surely.

1.4.1 Confidence intervals

To compute asymptotic confidence intervals, we analyze the asymptotic distribution of each of the four terms in the right hand side of the following error decomposition:

$$\log \hat{R} - \log R = (\log \hat{F}_1 - \log F_1) - (\log \hat{F}_0 - \log F_0) + (\log \hat{P}_1 - \log P_1) - (\log \hat{P}_0 - \log P_0). \quad (5)$$

From Assumption 1, the four terms are independent.

For the first two terms, assuming $\sqrt{S_t}(\hat{F}_t - F_t) \to \sigma_t Z_t$ in distribution, where Z_t are standard normal distributions, then the delta method implies under regularity conditions that $\sqrt{S_t}(\log \hat{F}_t - \log F_t) \to \frac{\sigma_t}{F_t} Z_t$ [4]. Here σ_t is estimated by applying the squared function into our conditional Monte Carlo estimator, i.e. by using $g(t) = (f(t))^2$,

$$\hat{G}_t := \frac{\sum_{j:D_t^j>0} g(D_t^j)}{S_t} \to G_t = \mathbb{E}[f^2(D_t)|D_t > 0],$$

from which we estimate σ_t with $\hat{\sigma}_t = \sqrt{\hat{G}_t - (\hat{F}_t)^2}$. Notice that if Assumption 3.1 holds for f, it automatically holds for g, however, Assumption 3.2 on g needs not follow from the same assumption on f.

Similarly, for P_t, we obtain that $(\log \hat{P}_t - \log P_t)$ can be approximated with a normal distribution of variance:

$$\begin{aligned}\frac{1}{I_t}\frac{P_t(1-P_t)}{P_t^2} &= \frac{1}{I_t}\frac{(1-P_t)}{P_t} \\ &= \frac{1}{I_t}\frac{(1-\frac{S_t}{I_t})}{\frac{S_t}{I_t}} \\ &= \frac{1}{S_t} - \frac{1}{I_t} \\ &\leq \frac{1}{S_t}.\end{aligned}$$

Putting it all together, we get that a conservative 95% asymptotic interval is given by:

$$\log \hat{R} \pm 1.96 \sqrt{\sum_{t=0}^{1} \frac{1}{S_t} \frac{\hat{G}_t}{(\hat{F}_t)^2}}. \tag{6}$$

So far we have ignored the fact that J_t^* is not known exactly but rather estimated. Suppose then that we observe from the initial condition barcodes (assumed distinct), J_0 of those corresponding to sgRNA $t = 0$ and J_1 of those corresponding to sgRNA $t = 1$. The ratio estimator $\hat{R}$ then becomes:

$$\begin{aligned} \hat{R}' &:= \frac{\sum_{j:D_0^j>0} f(D_0^j)}{J_0} \frac{J_1}{\sum_{j:D_1^j>0} f(D_1^j)} \\ &= R \frac{J_0^*}{J_0} \frac{J_1}{J_1^*}. \end{aligned}$$

To analyze R' we use a decomposition similar to Equation (5) but with additional terms taking into account the difference between J_t^* and J_t. The asymptotic variance of these additional terms are again analyzed using the delta method with the transform $\phi(x, y) = \log x - \log y$. We obtain the following inflation of the confidence interval:

$$\log \hat{R} \pm 1.96 \sqrt{\sum_{t=0}^{1} \frac{1}{S_t} \frac{\hat{G}_t}{(\hat{F}_t)^2} + \sum_{t=0}^{1} \frac{1}{J_t}}.$$

In our experiments we observed that the additional terms $1/J_t$ are dominated by the first terms.

1.4.2 Empirical validation of the calibration of the delta method confidence intervals

To empirically validate the calibration of the asymptotic confidence intervals described in the last section, we used a bootstrap strategy to simulate realistic synthetic datasets. In the following we focus on testing the estimator in Equation (6).

We first describe how we use the real data to inform the construction of the synthetic data. The main ingredient required to generate bootstrapped synthetic datasets is an assumed probability mass function $\hat{p}_t(d)$ for the random variables D_t. To do so we start by building an unnormalized histogram $\tilde{p}_t(d)$ from which we obtain $\hat{p}_t(d)$ by normalization, $\hat{p}_t(d) = \tilde{p}_t(d) / \sum_{d'=0}^{\infty} \tilde{p}_t(d)$. For values of d greater than zero, we set $\tilde{p}_t(d)$ based on empirical counts in a real dataset, i.e. for $d > 0$,

$$\tilde{p}_t(d) = \sum_{j=1}^{I_t} \mathbf{1}[D_t^j = d].$$

For the value of $\tilde{p}_t(d)$ at $d = 0$, we use the relation $E_t + S_t = I_t$, combined with the assumed identity $I_t \approx \lambda J_t$ and set $\tilde{p}_t(0) = \lambda J_t - S_t$.

We then simulated ten independent and identically distributed datasets, which can essentially be

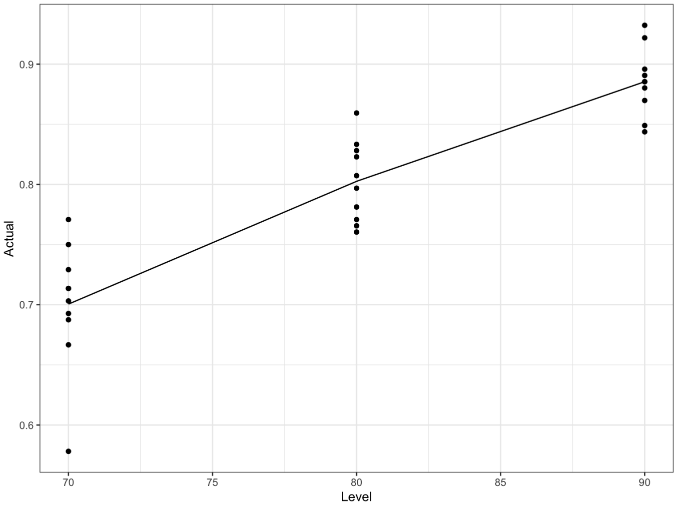

Figure S 12 Calibration of confidence intervals obtained using the delta method.

viewed as bootstrapped versions of a real dataset. More precisely, we re-sampled $\lfloor \lambda J_t \rfloor$ times from the distribution $\hat{p}_t(d)$, and dropped zeros. We used initial counts `PELib2_base_counts_2` with final conditions `C2191_4222341` and $\lambda = 10$. We computed 70%, 80%, and 90% confidence intervals using Equation (6) on ten bootstrapped datasets and looked at the fraction of intervals in each setting that contained the true value, defined as $\sum_{x=0}^{\infty} \hat{p}_t(x) f(x)$. The results shown in **Figure S12** confirm that the confidence intervals are calibrated.

1.5 Bayesian approach To gain statistical strength by combining several experiments, we also designed a Bayesian approach that estimates the fitness along with credible intervals. To move from a frequentist framework to a Bayesian one, in the following we change perspective and consider the variables I_t, J_t, S_t, E_t as random.

Recall Assumption 1, which encodes the hypothesis that the barcodes have a negligible effect on fitness. Encoding this hypothesis as exchangeability allows us to invoke de Finetti's theorem [5], which guarantees the existence of a random variable Ξ_t for each type t such that the observations D_t^{*j} are conditionally independent given Ξ_t. The random variable Ξ_t can be thought of as an abstract parameter governing the growth behaviour of type t.

De Finetti's theorem provides little information about the nature of Ξ and its distribution. For example Ξ can be an infinite dimensional object and this observation is a core motivation of Bayesian non-parametric statistics [6]. Since Bayesian non-parametric methods would be difficult to scale to the data at hand, we take an alternative method based on the construction of increasingly flexible parametric models $p(\cdot|\theta_t)$. The parameter θ_t is a vector characterizing the fitness behaviour of type t. We implemented and benchmarked a sequence of parametric families until a Bayesian goodness of fit check was passed.

Using these parametric models we can efficiently approximate the joint posterior distribution of $D_1, D_2, \ldots, D_{|\mathscr{T}|}$ conditionally on all data available Y. To summarize this posterior distribution, we use a Bayesian analogue B to the relative fitness relative fitness ratio R used for the frequentist ratio estimator

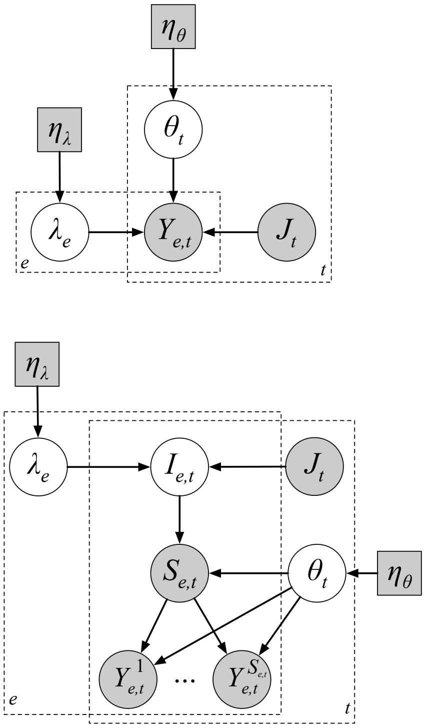

$$\begin{aligned} \lambda_e | \eta_\lambda &\sim \text{Exponential}(\cdot|\eta_\lambda) \\ I_{e,t} | \lambda_e, J_t &\sim \text{Poisson}(\cdot|\lambda_e J_t) \\ \theta_t | \eta_\theta &\sim G(\cdot|\eta_\theta) \\ D^j_{e,t} | \theta_t &\sim F(\cdot|\theta_t) \\ S_{e,t} &= \sum_{j=1}^{I_{e,t}} 1[D^j_{e,t} > 0] \end{aligned}$$

η_x	$\equiv$	Prior hyper-parameter for parameter x
J_t	$\equiv$	Initial counts of guide t measured from short run experiment
λ_e	$\equiv$	Poisson distribution scaling parameter for number of barcodes in experiment e
$\lambda_e J_t$	$\equiv$	Mean number of distinct barcodes of guide t in experiment e
$I_{e,t}$	$\equiv$	Number of distinct barcodes of guide t in experiment e
$S_{e,t}$	$\equiv$	Number of distinct barcodes of guide t in experiment e observed after censoring
θ_t	$\equiv$	Parameter controlling distribution of read counts for guide t
$F(\cdot|\theta_t)$	$\equiv$	Distribution of read counts for guide t e.g. mix-nb
$D^j_{e,t}$	$\equiv$	Number of reads for barcode j of guide t in experiment e

Figure S 13 Graphical models shared by all the Bayesian models considered in this paper. Upper: coarse view showing only the random variables used in the state space of the Markov chain Monte Carlo algorithm. Lower: more detailed generative process. The graphical model on the top is obtained by marginalization of the random variables $I_{e,t}$. Distributional assumptions and parameter descriptions are shown to the right.

Equation (2):

$$B = B_{1,0} := \frac{\mathbb{E}[f(D_1)|\Xi_1]}{\mathbb{E}[f(D_0)|\Xi_0]}. \tag{7}$$

Our method summarizes the posterior distribution on the random variable B conditionally on Y using both point estimates and credible intervals (Section 1.5.5).

1.5.1 General structure of the models and graphical models

The broad structure of the model is shown in **Figure S13**. The index e ranges over experimental replicates performed in the same conditions. The index $t \in \mathscr{T}$ ranges over sgRNA types, and the observed random variable J_t encodes initial condition measurement (see Section 1.1 for details).

The observations $Y_{t,e}$ consist in the subset of barcode counts $D^j_{t,e}$ such that $D^j_{t,e} > 0$. There is one such subset of observations available for each experimental replicate e. The random variables λ_e is introduced in Assumption 4. The Bayesian model posits the same assumption of proportionality as used by the ratio estimator, while allowing the proportionality factor to vary from one experimental replicate to another. In contrast to the ratio estimator, the proportionality factors are imputed. We denote the prior on each type-specific sgRNA parameter by $\theta_t \sim p(\cdot)$. We denote the shared probability mass function of the random variables $D^j_{t,e}$ by $p(\cdot|\theta_t)$. In all models considered, we use an exponential prior on the random variables λ_e

with rate $1/100$.

To understand how the assumed distribution on the observed final counts $Y_{e,t}$ is constructed, we describe now a more detailed account of the assumed generative process taking into account the censoring of the random variables with $D^j_{t,e} = 0$.

We posit

$$I_{e,t}|\lambda_e, J_t \sim \text{Poi}(\lambda J_t). \tag{8}$$

For computational tractability, during inference it is helpful to analytically marginalize the random variables $I_{e,t}$. This is done based on the following property: as a consequence of Equation (8), we can view the censoring as a thinned Poisson process [7]. To see why, note that the Poisson process can be seen as having $I_{e,t}$ points in it, each point associated with a variable $D^j_{t,e}$, $j \in \{1, 2, \ldots, I_{e,t}\}$. We simulate each variable $D^j_{t,e}$ in two steps: first, flip a Bernoulli to decide if $D^j_{t,e}$ takes value zero or not. Second, for the points where $D^j_{t,e} > 0$, sample from the conditional distribution

$$p(d|\theta_t, D_{t,e} > 0) = \frac{p(d|\theta_t)}{1 - p(0|\theta_t)}.$$

The first step is equivalent to a Poisson thinning based on Bernoulli random variables which keep points in the Poisson process with probability $\mathbb{P}(D_{t,e} > 0|\theta_t) = 1 - p(0|\theta_t)$. It follows from the Thinning Theorem [7] that $S_{e,t}|\lambda_e, \theta_t, J_t \sim \text{Poi}(\lambda_e J_t(1 - p(0|\theta_t)))$. Hence:

$$\mathbb{P}(S_{e,t} = s_{e,t}, Y_{e,t} = y_{e,t}|\lambda_e, \theta_t, J_t) = \text{Poi}(s_{e,t}; \lambda_e J_t(1 - p(0|\theta_t))) \prod_{j=1}^{S_{t,e}} p(y^j_{t,e}|\theta_t, D_{t,e} > 0), \tag{9}$$

where $y^j_{t,e}$ is the j-th non-zero element in $D^1_{t,e}, D^2_{t,e}, \ldots, D^{I_{t,e}}_{t,e}$. Notice that several factors in this product are identical, namely those with the same value $y^j_{t,e}$. Hence computation can be sped up by grouping these identical factors and exponentiating each one by the number of barcodes with a given size. A related fact is that the empirical histogram of barcode sizes is a sufficient statistic to compute the likelihood of our model.

We did not model the sampling noise between J_t and J^*_t since we found that this part of the noise had a negligible effect in the related ratio estimator. If outliers are present in the initial condition counts J_t, it may be useful to add a simple multinomial error model between J_t and J^*_t.

The main ingredient missing in this description is the specific form of the prior $p(\theta_t)$ along with the probability mass function of $D_{t,e}$, denoted $p(d|\theta_t)$. We address this in the next section.

1.5.2 Clone size distribution families

To build a suitable model for the distributions $D_{t,e}$, we performed Bayesian model selection and goodness of fit experiments comparing the following parametric families $p(\cdot|\theta_t)$: the Poisson and the Yule-Simon families, for which the parameter for one sgRNA type, θ_t, is univariate; the Negative-Binomial family, for

which θ_t is bivariate; and the Beta-Negative-Binomial family, which is trivariate. We then considered mixtures having two components, namely mixtures of Yule-Simon distributions, mixtures of Negative-Binomial distributions, and mixtures of Beta-Negative-Binomial distributions.

Priors for the parameters were set as follows: unless mentioned otherwise, for parameter components having a support on $(0, \infty)$, we used an exponential prior with rate $\eta = 1/100$. For mixture proportions π, unless mentioned otherwise, we used a uniform prior on $(0, 1/2)$ to break symmetry between the two mixture components. This forces the first mixture component to be the minor one and helps interpretability of the posterior distributions. For the Negative Binomial distribution, instead of using the traditional parameterization in terms of r and p we use an equivalent but more interpretable parameterization in terms of a mean parameter $\mu > 0$ and an overdispersion parameter $\tau > 0$.

The parametric families along with associated prior distributions are defined below. In what follows we use $\Gamma(x)$ to denote the Gamma function. The variables θ and π below are type-specific, but we omit the subscripts t for simplicity.

Poisson

$$\begin{aligned} \theta|\eta_\theta &\sim \text{Exponential}(\cdot|\eta_\theta) \\ p(x|\theta) &= \frac{\theta^x e^{-\theta}}{x!} \mathbf{1}[x \in \{0, 1, 2, \dots\}] \end{aligned}$$

Negative Binomial

$$\begin{aligned} \theta &= (\mu, \tau) \\ \theta_i|\eta_i &\sim \text{Exponential}(\cdot|\eta_i) \\ r &= \frac{\mu^2}{\tau} \\ p &= 1 - \frac{\mu}{\mu + \tau} \\ p(x|\theta) &= \binom{x + r - 1}{x} p^x (1 - p)^r \; \mathbf{1}[x \in \{0, 1, 2, \dots\}] \end{aligned}$$

Beta Negative Binomial

$$\begin{aligned} \theta &= (a, b, r) \\ \theta_i|\eta_i &\sim \text{Exponential}(\cdot|\eta_i) \\ p(x|\theta) &= \frac{\Gamma(x + r)}{x!\Gamma(r)} \frac{\Gamma(a + x)\Gamma(b + r)}{\Gamma(a + b + r + x)} \frac{\Gamma(a + b)}{\Gamma(a)\Gamma(b)} \mathbf{1}[x \in \{0, 1, 2, \dots\}] \end{aligned}$$

Yule Simon

$$\begin{aligned}\theta|\eta_\theta &\sim \text{Exponential}(\cdot|\eta_\theta)\\ p(x|\theta) &= \theta\frac{\Gamma(1+x)\Gamma(1+\theta)}{\Gamma(2+x+\theta)}\mathbf{1}[x\in\{0,1,2,\dots\}]\end{aligned}$$

Mixture

$$\begin{aligned}\theta &= (\theta_1,\theta_2)\\ \pi &\sim \text{Uniform}(0,1/2)\\ p(x|\theta) &= \pi p_1(x)+(1-\pi)p_2(x)\end{aligned}$$

To unify the notation across all these models, we denote the conditional distribution $\theta_t|\eta_\theta$ by $G(\cdot|\eta_\theta)$, and the conditional distribution of $D^j_{t,e}|\theta_t$ by $F(\cdot|\theta_t)$.

We observed that for two of the mixture models considered, namely the mixture of Negative Binomial distributions and the mixture of Yule Simon distributions, the automatic initialization of the model (described in [8]) led in certain cases to numerical problems in the evaluation of the likelihood at the first iteration (obtaining a log likelihood with value "NaN"). Since the initialization is based on the prior, we addressed this issue using a slight modification of our default prior choices: we used a rate of $\eta = 1$ for one of the components prior parameters, while leaving a vague prior for the other component. Since this makes the priors non-symmetric, we also changed the prior on π to be uniform on $(0,1)$ instead of $(0,1/2)$, the latter being only warranted for mixtures where the two components have identical prior distributions.

1.5.3 Relaxed assumptions on $I_{e,t}$

We also considered a relaxation of the distributional assumption in Equation (8), introducing one separate parameter $\lambda_{e,t}$ for each replicate e and target t, instead of sharing the parameter λ_e across the different types within one replicate:

$$\begin{aligned}\alpha_e|\eta_\alpha &\sim \text{Exp}(\eta_\alpha)\\ \beta_e|\eta_\beta &\sim \text{Exp}(\eta_\beta)\\ \lambda_{e,t}|\alpha_e,\beta_e &\sim \text{Gamma}(\alpha_e,\beta_e)\\ I_{e,t}|\lambda_{e,t},J_t &\sim \text{Poi}(\lambda_{e,t}J_t)\end{aligned}$$

We call this model the local-λ variant. We set $\eta_\alpha = \eta_\beta = 1/100$. The modified graphical model shown in **Figure S14**.

In the previous version of the model, the random variables $I_{e,t}$ were analytically marginalized. In the

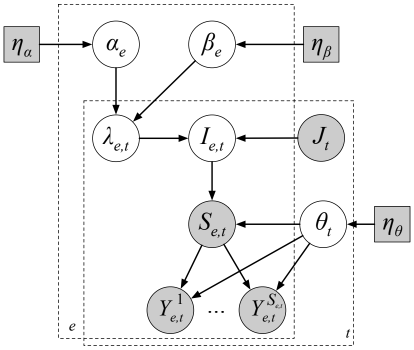

Figure S 14 Graphical model used in the local-λ variant.

local-λ variant, we marginalize both $\lambda_{e,t}$ and $I_{e,t}$. Equation (9) becomes:

$$\mathbb{P}(S_{e,t} = s_{e,t}, Y_{e,t} = y_{e,t}|\lambda_{e,t}, \theta_t, J_t) = \frac{(J_t(1-p(0|\theta_t))^{s_{e,t}}\Gamma(\alpha_e + s_{e,t})\beta_e^{\alpha_e}}{\Gamma(\alpha_e)\Gamma(s_{e,t}+1)(\beta_e + J_t(1-(1-p(0|\theta_t))^{\alpha_e+s_{e,t}}} \prod_{j=1}^{S_{t,e}} p(y^j_{t,e}|\theta_t, D_{t,e} > 0).$$

1.5.4 Multiple infection model

So far, we have assumed that each cancer cell was infected by at most one virus. In practise, cells can be infected by several viruses and hence may be affected by several distinct edits simultaneously. The distribution of the number of infections can be approximated by Poisson distribution with mean parameter given by the multiplicity of infection [9]. Here we propose a model extension that goes beyond simple estimation of the number of multiply infected cells, and "deconvolves" the signal by imputing a fraction of the variability coming from multiple infections.

Let $M^j_{t,e}$ denote the multiplicity of infection for UMI j, target t and experiment e. We consider the following decomposition:

$$D^{j,\text{MOI}\geq 1}_{t,e} = D^{j,\text{MOI}=1}_{t,e}\mathbf{1}[M^j_{t,e} = 1] + D^{j,\text{MOI}>1}_{t,e}\mathbf{1}[M^j_{t,e} > 1], \quad (10)$$

which separates the cases where a cell was infected once from the case where a cell was infected multiple times. We have $M^j_{t,e} \sim$ Bernoulli(π^{MOI}), and we assume a flat prior for the probability of multiple infection (which could be made experiment-specific if believed to markedly differ across replicates). We assume $D^{j,\text{MOI}=1}_{t,e} \overset{d}{=} D_t$ and $D^{j,\text{MOI}>1}_{t,e} \overset{d}{=} D_{\text{MOI}}$, where $D_{\text{MOI}} \sim$ NegativeBinomial$(\mu_{\text{MOI}}, \tau_{\text{MOI}})$, $\mu_{\text{MOI}} \sim$ Exponential(η_{MOI}), $\tau_{\text{MOI}} \sim$ Exponential(η_{MOI}).

The remaining of the model is build as before, yielding:

$$
\begin{aligned}
\lambda_e|\eta_\lambda &\sim \text{Exponential}(\cdot|\eta_\lambda)\\
I_{e,t}|\lambda_e, J_t &\sim \text{Poisson}(\cdot|\lambda_e, J_t)\\
\theta_t|\eta_\theta &\sim G(\cdot|\eta_\theta)\\
D_{t,e}^{j,\text{MOI}=1}|\theta_t &\sim F(\cdot|\theta_t)\\
\pi^{\text{MOI}} &\sim \text{Uniform}(\cdot|0,1)\\
M_{t,e}^j|\pi^{\text{MOI}} &\sim \text{Bernoulli}(\cdot|\pi^{\text{MOI}})\\
\mu_{\text{MOI}} &\sim \text{Exponential}(\cdot|\eta_{\text{MOI}})\\
\tau_{\text{MOI}} &\sim \text{Exponential}(\cdot|\eta_{\text{MOI}})\\
D_{t,e}^{j,\text{MOI}>1}|\mu_{\text{MOI}}, \tau_{\text{MOI}} &\sim \text{NegativeBinomial}(\cdot|\mu_{\text{MOI}}, \tau_{\text{MOI}})\\
D_{t,e}^{j,\text{MOI}\geq 1} &= D_{t,e}^{j,\text{MOI}=1}\mathbf{1}[M_{t,e}^j = 1] + D_{t,e}^{j,\text{MOI}>1}\mathbf{1}[M_{t,e}^j > 1]\\
S_{e,t} &= \sum_{j=1}^{I_{e,t}} \mathbf{1}[D_{t,e}^{j,\text{MOI}\geq 1} > 0].
\end{aligned}
$$

For inference, the random variables $M_{t,e}^j$, $D_{t,e}^{j,\text{MOI}>1}$ and $D_{t,e}^{j,\text{MOI}=1}$ are marginalized by noting that the conditional distribution of $D_{t,e}^{j,\text{MOI}\geq 1}$ given $\pi, \pi^{\text{MOI}}, \mu_{\text{MOI}}, \tau_{\text{MOI}}, \theta$ is:

$$
p(d|\pi, \pi^{\text{MOI}}, \mu_{\text{MOI}}, \tau_{\text{MOI}}, \theta) = \pi^{\text{MOI}} p_{\text{NB}}(d|\mu_{\text{MOI}}, \tau_{\text{MOI}}) + (1 - \pi^{\text{MOI}})(\pi p_{\text{NB}}(d|\theta_1) + (1 - \pi p_{\text{NB}}(d|\theta_2))).
$$

Based on this identity, the same simplifications as in Section 1.5.1 can be applied. Inference in both the multiple infection case and the simple case is described in more details in Section 1.5.5 — since they are mathematically equivalent, we describe inference in the latter case for notation simplicity.

The output of the multiple-infection model is then obtained from the single infection distribution component of the decomposition (10), namely by using Equation (7) on the random variable D_t equal in distribution to $D_{t,e}^{j,\text{MOI}=1}$.

1.5.5 Inference

All parametric families were implemented in the Blang probabilistic programming language [8], which automates inference based on Markov chain Monte Carlo and sequential Monte Carlo. One of the key motivations for choosing this probabilistic programming language is that it can provide reliable marginal likelihood estimates for large models, by automatically creating parallel "heated" chains (for example, in Section 1.5.7, we used 500 chains for one of the experiments).

The Blang code used can be found at `https://github.com/UBC-Stat-ML/nowellpack` (DOI: 10.5281/zenodo.6595131). All analysis performed in this manuscript used the tagged version `humi-v1.2` [10]. Pipeline code can be found at `https://github.com/aroth85/humi_pipeline` (10.5281/zenodo.6596290) [11]. The models described in the preceding section are implemented in the package:

https://github.com/UBC-Stat-ML/nowellpack/tree/master/src/main/java/humi/models.

The state space of the Markov chain consists in a vector θ_t for each type $t \in \mathscr{T}$ as well as one experimental condition specific scaling factor λ_e, $e \in \mathscr{E}$. For the λ-local variant, the state space is based instead of λ_e on a rate and scale parameter for each $e \in \mathscr{E}$, α_e, β_e. We use the notation $\theta = (\theta_1, \theta_2, \ldots, \theta_{|\mathscr{T}|})$ and $\lambda = (\lambda_1, \lambda_2, \ldots, \lambda_{|\mathscr{E}|})$. The random variables $I_{e,t}$ and π_t are marginalized analytically. In the λ-local version, $\lambda_{t,e}$ is also marginalized. Marginalization of $I_{e,t}$ is implemented in
src/main/java/humi/CensoredExchangeableCounts.bl,
joint marginalization of $\lambda_{e,t}, I_{e,t}$ in the λ-local model is implemented in
src/main/java/humi/MarginalizedGammaCensoredCounts.bl, marginalization of π_t is implemented in
src/main/java/humi/IntMixture.bl.
From the Markov chain Monte Carlo output, we obtain samples $(\theta^{(1)}, \lambda^{(1)}), (\theta^{(2)}, \lambda^{(2)}), \ldots, (\theta^{(n)}, \lambda^{(n)})$, where n denotes the number of samples and can be viewed as a tuning parameter controlling the quality of the approximation of the posterior distribution. In the λ-local version, the samples take the form $(\theta^{(1)}, \alpha^{(1)}, \beta^{(1)}), (\theta^{(2)}, \alpha^{(2)}, \beta^{(2)}), \ldots, (\theta^{(n)}, \alpha^{(n)}, \beta^{(n)})$.

We tested the correctness of the implementation of our method using Fisherian hypothesis test of equality of *marginal-conditional* and *successive-conditional* distributions [12], using the *exact invariance* testing framework implemented in Blang. Specifically, our tests, implemented in /src/test/java/humi/TestS
cover our implementation of the three analytic marginalization model components described above:

- CensoredExchangeableCounts.bl
- MarginalizedGammaCensoredCounts.bl
- IntMixture.bl

The test did not reject the null hypothesis that the forward and posterior simulation code agree at the 5% level. Testing the implementation of the parametric families described in Section 1.5.2 is already covered by the test suite included in the Blang SDK. All tests were automatically performed for all commits in the repositories, the test results for all code commits are available at https://travis-ci.org/github/UBC-Stat-ML/nowellpack/branches and https://travis-ci.org/github/UBC-Stat-ML/blangSDK/branches.

Sampling was performed using Blang's default configurations unless noted otherwise. Briefly, sampling is based on adaptive non-reversible parallel tempering [13] with an initialization obtained from an adaptive annealed sequential Monte Carlo algorithm [14]. Sampling of the individual variables was performed using slice sampling via the doubling procedure from [15]. In all experiments, the first 50% of the samples were discarded as burn-in.

We constructed point estimates and credible intervals for $B_{1,0}|Y$ from the post burn-in samples $\theta^{(n/2)}, \theta^{(n/2+1)}, \ldots$

as follows. Let us focus on a single pair of targeting sgRNA t and reference sgRNA t'. First, we compute

$$B^{(i)} = B^{(i)}_{1,0} = \frac{\mathbb{E}[f(D_1)|\theta^{(i)}_1]}{\mathbb{E}[f(D_0)|\theta^{(i)}_0]}. \tag{11}$$

If $f(d) = d$, then this can be computed analytically from the known mean of the parametric families in Section 1.5.2. For example, since the mean of a Negative Binomial is given by its first parameter, $\theta^{(i)}_t = (\mu^{(i)}_t, \tau^{(i)}_t)$, then Equation (11) reduces to

$$B^{(i)}_{1,0} = \frac{\mu^{(i)}_1}{\mu^{(i)}_0}.$$

If f is the conditional Winsorized mean introduced in Section 1.3.1, then each conditional expectation can be computed numerically:

$$\mathbb{E}[f(D_t)|\theta^{(i)}_t] = \sum_{d=0}^{d^{*(i)}_t} d \times p(d|\theta^{(i)}_t) + d^{*(i)}_t \left(1 - \sum_{d=0}^{d^{*(i)}} p(d|\theta^{(i)}_t)\right) \tag{12}$$

$$d^{*(i)}_t = \inf\left\{d' : 0.95 \le \sum_{d=1}^{d'} p(d|\theta^{(i)}_t)\right\}.$$

Second, the set of samples $B^{(i)}_{1,0}$ is summarized using standard Bayesian methods. Specifically, we use a median point estimate and a $(1-\alpha)$-credible interval with end points at the quantiles $\alpha/2$ and $1-\alpha/2$.

1.5.6 Goodness of fit

We used a procedure based on posterior predictive checks [16] to assess the goodness of fit of the various models described in Section 1.5.2. Briefly, we selected a suite of type-specific observed summary statistics and computed posterior predictive distributions for these statistics. We then constructed $\gamma \times 100\%$ credible intervals for each posterior predictive distribution and measured if this interval contains the observed value. We then computed the frequency across the different types at which the observed value falls within the credible interval. Frequency can be thought of as an approximation of a frequentist coverage probability, which can be compared to the nominal probability γ. A large divergence between the nominal and actual coverage probability is interpreted as a gross model mis-specification.

We considered the following observed statistics:

Visible number of clones: the observed random variable $S_{t,e}$ as introduced in Section 1.1. To build a predictive distribution for this random variable, we proceed as follows. For each MCMC sample for $\theta^{(i)}_{t,e}, \lambda^{(i)}_e$, compute $p^{(i)}_0 = p(0|\theta^{(i)}_t)$, and $\tilde{S}^{(i)}_{t,e} \sim \text{Poi}((1-p^{(i)}_0)J_{t,e}\lambda^{(i)}_e)$. We then create for each t and e a $\gamma\%$ credible intervals based on symmetric two-tailed quantiles of $\tilde{S}^{(1)}_{t,e}, \tilde{S}^{(2)}_{t,e}, \dots$. The analogue for the λ-local model variant uses $\lambda^{(i)}_e|\alpha^{(i)}, \beta^{(i)} \sim \text{Gamma}(\alpha^{(i)}, \beta^{(i)})$.

Truncated mean clone size: based on a truncation interval T_i which should exclude zero in order to be

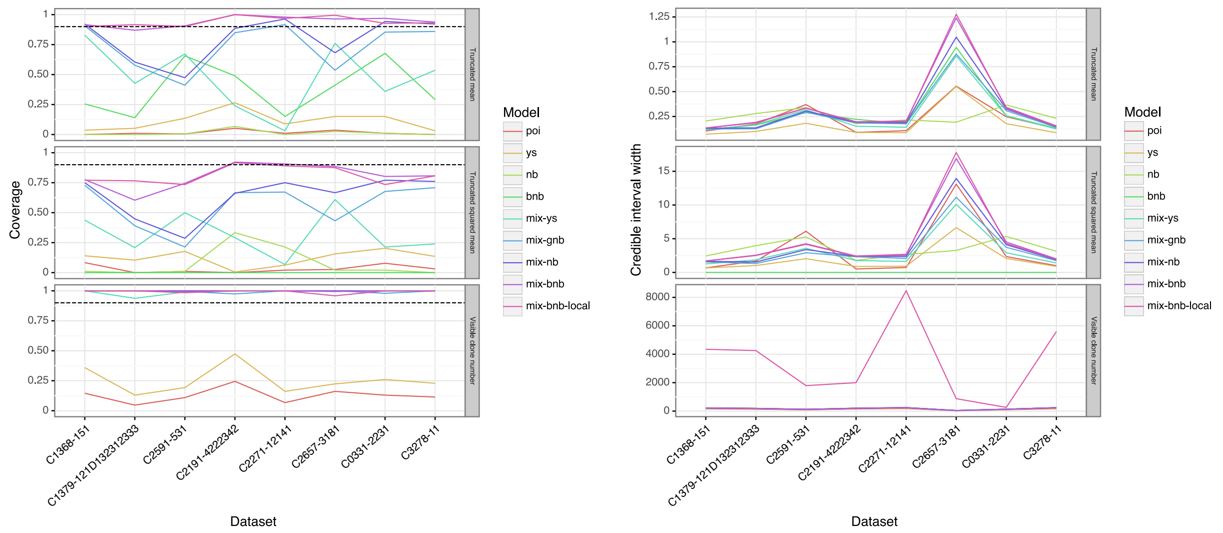

Figure S 15 Bayesian goodness of fit results. The three facet rows denote the three observed statistics. Model abbreviations: Beta Negative Binomial (bnb), λ-local Mixture of Beta Negative Binomial (Section 1.5.3, mix-bnb-local), Mixture of Beta Negative Binomial (mix-bnb), Mixture of Negative Binomial (mix-nb), Mixture of Yule Simon (mix-ys), Negative Binomial (nb), Poisson (poi), Yule Simon (ys). The ordinate axis shows a representative subset of datasets, including biological replicates of final conditions (C2191-4222341–4) and different initial conditions (denoted by the suffix initial1). Left: actual coverage of nominal 90% credible intervals (optimal value shown as an horizontal black dashed line). Right: average width of the intervals.

computable from the observations. We used $T_{\mathrm{i}} = [1, 20]$. This truncated mean can be computed from observed data as

$$\frac{\sum_{j=1}^{I_t} \mathbf{1}[D_{t,e}^j \in T_{\mathrm{i}}] f(D_{t,e}^j)}{\sum_{j=1}^{I_t} \mathbf{1}[D_{t,e}^j \in T_{\mathrm{i}}]},$$

where $f(d) = d$. The random variable can also be imputed from an MCMC sample $\theta^{(i)}$ via

$$\frac{\sum_{d \in T_{\mathrm{i}}} d \times p(d|\theta_t^{(i)})}{\sum_{d \in T_{\mathrm{i}}} p(d|\theta_t^{(i)})}.$$

Truncated squared mean clone size: constructed in the same way as the truncated mean clone size but using $f(d) = d^2$ instead of $f(d) = d$.

The results are summarized in **Figure S15**. Many of the models based on mixtures achieve a coverage close to nominal, particularly the models based on mixtures of Beta Negative Binomial (mix-bnb). Comparing the two flavours of mix-bnb (mix-bnb and mix-bnb-local, Section 1.5.3), the λ-local version appears to yield unnecessarily large visible clone number intervals for a similar coverage. All the models that are not based on mixture models are severely misfit.

Model	poi	ys	nb	bnb	mix-ys	mix-gnb	mix-nb	mix-bnb	mix-bnb-loc
Dataset									
C1368-151	−1578.57	−514.45	−594.57	−464.29	**−404.68**	−417.66	−413.47	−409.65	−408.98
C1379-121D132312333	−13388.70	−1728.43	−2227.46	−1621.14	**−1580.93**	−1597.81	−1593.52	−1591.94	−1591.13
C2591-531	−1104.73	−271.90	−302.89	−259.44	**−241.50**	−251.71	−247.22	−247.13	−242.74
C2191-4222342	−862.89	−516.25	−542.08	−505.21	**−486.11**	−502.74	−499.60	−493.41	−491.75
C2271-12141	−11283.87	−3926.99	−4248.83	−3631.81	−3425.44	−3415.55	−3412.29	**−3407.66**	−3407.90
C2657-3181	−67.69	−48.56	−42.91	−46.88	−43.88	−44.91	−42.41	−46.55	**−41.43**
C0331-2231	−2246.12	−547.89	−588.49	−514.42	**−470.41**	−481.09	−476.60	−473.34	−472.32
C3278-11	−12623.79	−2679.76	−3172.02	−2372.67	**−2130.75**	−2144.23	−2139.56	−2133.22	−2135.91

Table 5: Evidence estimates for different clone size distributions. Values indicated in bold text are the best for a dataset.

1.5.7 Bayesian model selection

A complementary method to perform model selection within the Bayesian framework is to compute the probability assigned to the observed data, $\mathbb{P}_m(Y = y)$ for each model at hand, $m \in$ {bnb, mix-bnb-local, mix-bnb, ..., ys} [17]. The quantity $\mathbb{P}_m(Y = y)$ is known as the marginal likelihood or evidence.

The results in this section use the stepping stone estimator [18] used by Blang to automatically estimate $\mathbb{P}_m(Y = y)$ from the output of non-reversible parallel tempering. For the first set of experiments, we use a subset with $|\mathscr{T}| = 1$ to speed-up computation. We show the progress of the stepping stone estimator as a function of the parallel tempering adaptation rounds in **Figure S16**, showing that these estimates converge quickly in terms of the number of Markov chain Monte Carlo iterations.

The evidence scores for the different models are shown in Table 5. The results confirm that the largest improvement is achieved by the introduction of mixture models. Note that Bayesian model selection automatically takes into account the complexity of the model (number of parameters) as well as potential non-identifiability.

Next, we compared the model with multiple infection modelling (Section 1.5.4) with two representative models that do not take multiple infection modelling into account (MixNB and NB). Since the multiple infection model requires sharing statistical strength across sgRNAs, we remove the restriction $|\mathscr{T}| = 1$ and increase the computational budget from 20 heated chains in **Figure S16**, to 500 heated chains **Figure S17**. We found that the multiple infection model does increase the marginal likelihood, however the increase is negligible compared to the increase in marginal likelihood obtained from going from NB to MixNB.

1.5.8 Prior sensitivity

The main tuning parameters in prior distributions described in Section 1.5.2 are the hyper-parameters η. controlling the dispersion of the top level priors. By default, these hyper-parameters are all set to $\eta = 1/100$. To assess the sensitivity of this tuning parameter, we performed a set of experiments where we set η to a value one order of magnitude lower ($1/1000$) and higher ($1/10$). The results are shown in **Figure S18**. The vast majority of the intervals and point estimates are highly similar across the three orders of magnitudes for

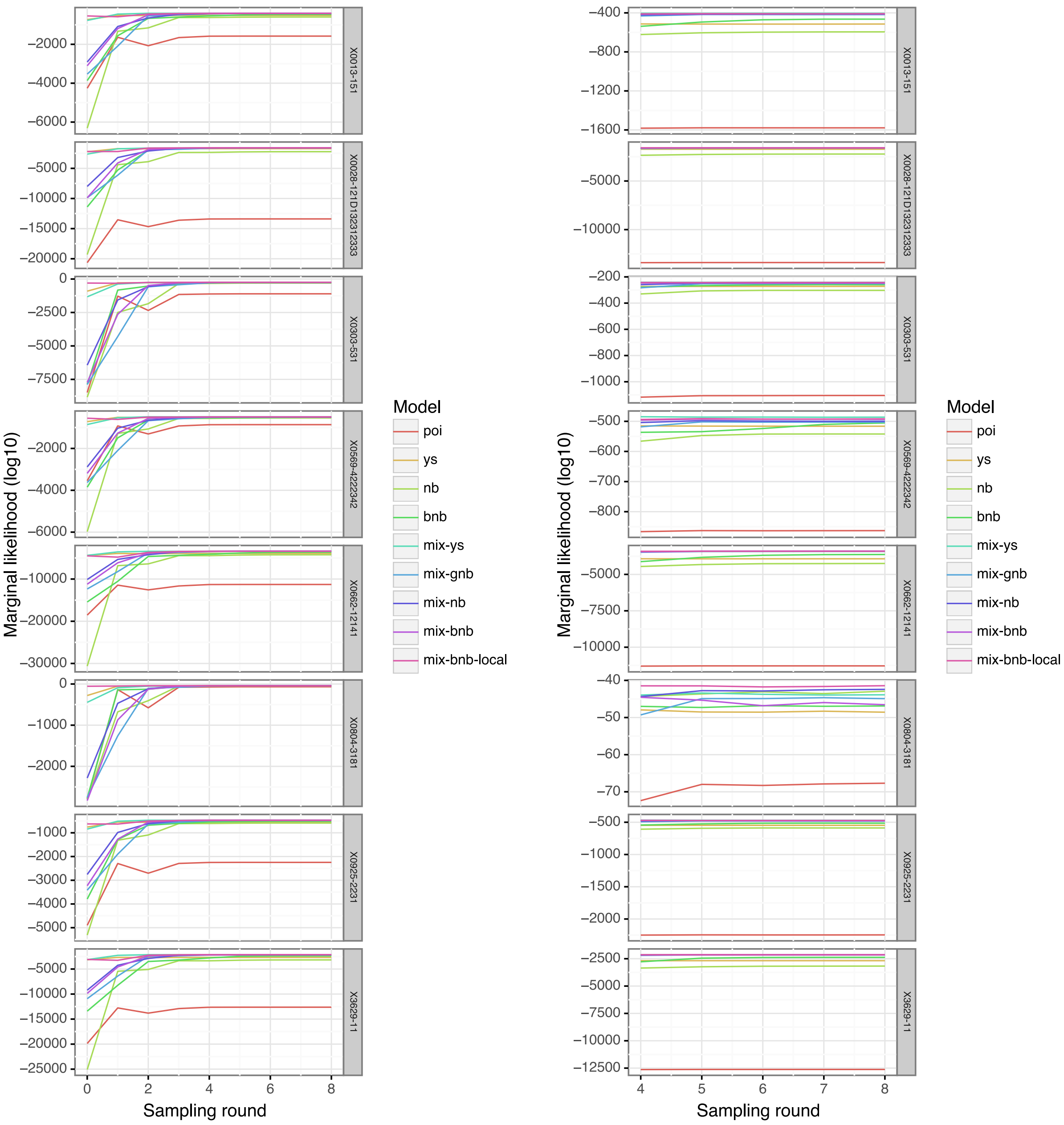

Figure S 16 Convergence of the stepping stone evidence estimate with $|\mathscr{T}| = 1$ to speed-up computation.. Left: across all adaptation rounds. Right: for the last five adaptation rounds.

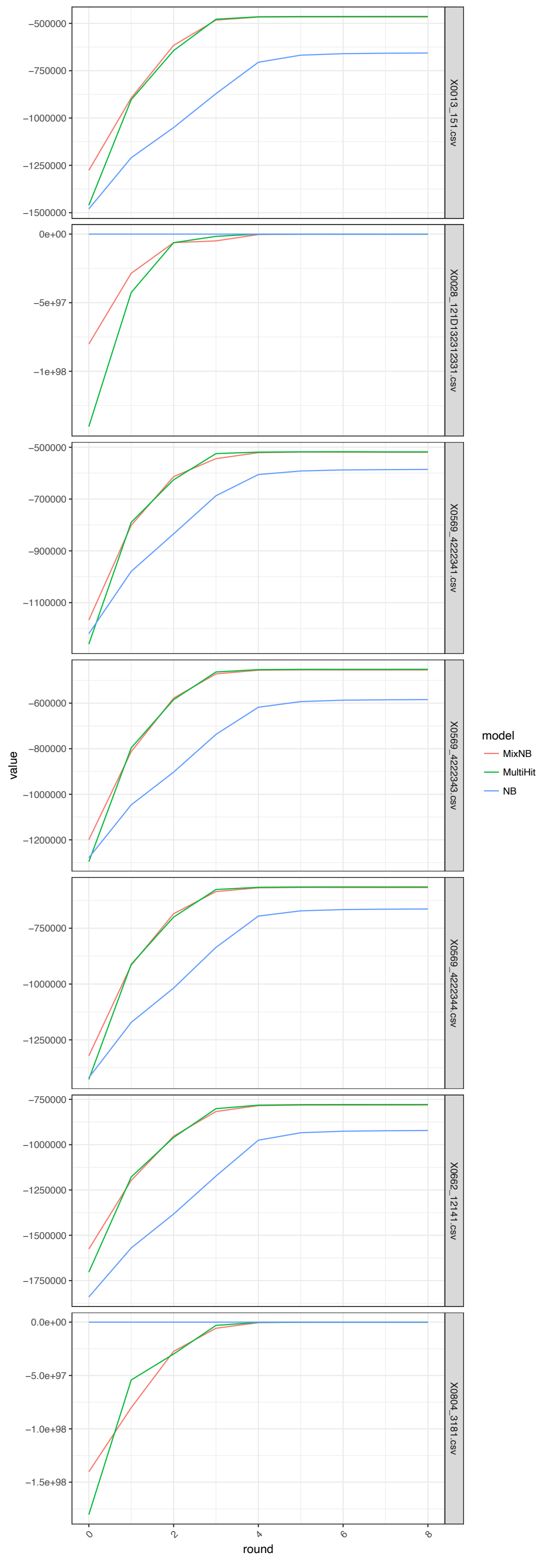

Figure S 17 Convergence of the evidence estimates for the multiple infection model, as well as two models that do not take multiple infection into account, namely MixNB and NB. In these comparisons, we remove the restriction $|\mathscr{T}| = 1$.

the prior tuning parameter. Most exceptions occur in the dataset C2657-3181 (right-most column), which has lower sgRNA-UMI diversity than the other datasets and shows less good fit with the mix-nb model (**Figure 1**a, **Figure 2**c,f). In the other datasets, the main difference is that the intervals tend to be slightly larger in the regime $\eta = 0.001$, but with still a large overlap with the other prior regimes.

1.6 Choice of reference Both frequentist and Bayesian estimators, R_{t,t_0}, B_{t,t_0} rely on a fixed reference type t_0 based on which the fitness of various other targeting sgRNA $t : t \neq t_0$ is estimated. When non-targeting guides are available, a simple option for t_0 is to relabel all the non-targeting guides as being of a common type t_0.

We also considered an alternative which does not require non-targeting guides. This alternative is based on the observation that when we ranked the guides in our panel by their fitnesses, we observed a central "plateau" sharing the non-targeting guides neutrality. In the Bayesian setup this can be used to estimate a reference as follows. Define the random variables $E_t = \mathbb{E}[f(D_t)|\theta_t]$ and the quantile function $q(x_1, x_2, \dots)$. Then we introduce a random reference $T = q(E_1, E_2, \dots, E_{|\mathscr{T}|})$ and define fitness with respect to it: $\tilde{B} = B_{t,T}$. The distribution of $\tilde{B}|Y$ is summarized as follows. First, for each MCMC iteration i and type t, compute $E_t^{(i)}$ using the same method as described in Section 1.5.5. Second, compute $T^{(i)} = q(E_1^{(i)}, E_2^{(i)}, \dots, E_{|\mathscr{T}|}^{(i)})$. Third, compute $\tilde{B}_t^{(i)} = \frac{E_t^{(i)}}{E_{T^{(i)}}^{(i)}}$. Then from $\tilde{B}_t^{(i)}$ compute credible intervals as in Section 1.5.5.

1.7 Poset framework The methodology described in Section 1.5.5 allows us to compute point estimates for fitness and credible intervals which reflect our certainty in those estimates. We use the credible intervals to assess whether there is a significant difference if fitness between a pair of guides from a single condition. Let t_1 and t_2 be a pair of guides and $I_i = [l_i, r_i]$ denote the α level credible interval of guide i. Then we say that guide 1 is significantly fitter than guide 2, denoted $t_1 \succeq t_2$, if $I_1 \cap I_2 = \emptyset$ and $l_1 > r_2$. Stated plainly we say there is a significant difference if the credible intervals do not overlap, and the guide with the interval to the right (higher) is fitter.

By definition if $t_1 \succeq t_2$ and $t_2 \succeq t_3$ then $t_1 \succeq t_3$. If the intervals of t_1 and t_2 overlap then neither $t_1 \succeq t_2$ nor $t_2 \succeq t_1$ is true and we say that the guides are not comparable. Thus the relationship $\succeq$ induced by comparing fitness of guides is a partial ordering (poset) on $\mathscr{T}$ [19]. The complete set of relationships can be represented by a graph with $|\mathscr{T}|$ nodes representing guides, and directed edges between t_i and t_j whenever $t_i \succeq t_j$. Visualizing this graph is difficult as the number of nodes is $\mathcal{O}(|\mathscr{T}|)$ and the number of edges is $\mathcal{O}(|\mathscr{T}|^2)$. Instead we use a Hasse diagram [19] which is a compact representation of the poset graph from which all edges in the original poset can be inferred. Consider again the case where we have $t_1 \succeq t_2$ and $t_2 \succeq t_3$ which implies $t_1 \succeq t_3$. Then the full poset graph would have three edges, (t_1, t_2), (t_1, t_3) and (t_2, t_3). However, the Hasse diagram would only have two edges, (t_1, t_2) and (t_2, t_3). The existence of (t_1, t_3) can be inferred because there is a path in the Hasse diagram from t_1 to t_3.

1.8 Difference criteria in comparisons When carrying out pairwise comparisons between experiments (in which each experiment can be a single datasets or a set of replicate datasets), we identified a subset of outlying targeting guides that satisfied all four of the following criteria based on the fitness and rank differences.

1. **Fitness difference criterion**

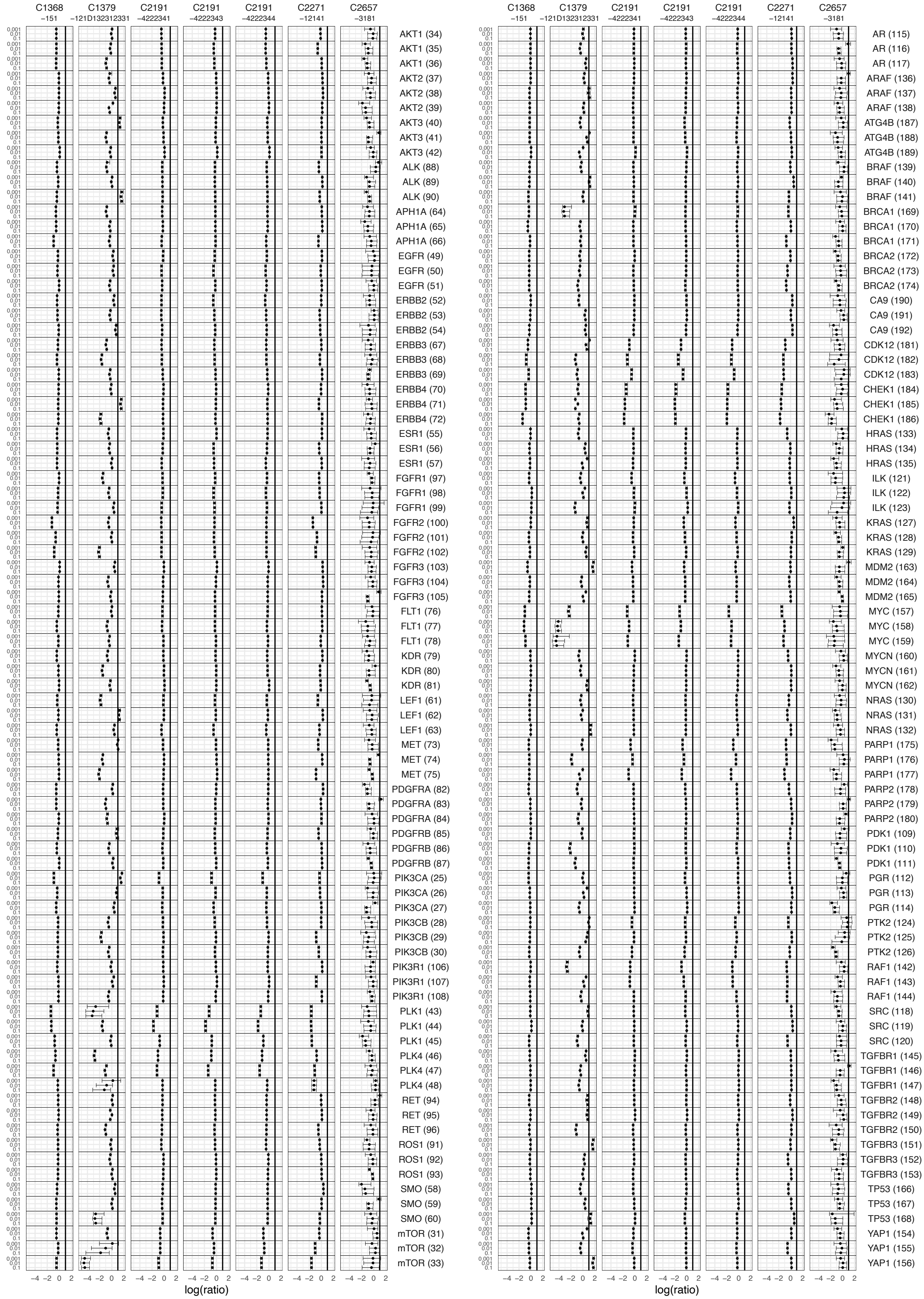

Figure S 18 Prior sensitivity analysis: comparison of the credible intervals obtained when varying the prior distributions. Row triples indicate three different order of magnitude for the prior hyper-parameters η's in Section 1.5.2.

For two experimental conditions a and b, let $\theta_{t,a}$ and $\theta_{t,b}$ denote the respective fitness parameters for each sgRNA t. We estimate the difference in Winsorized mean fitness $\Delta_t = \mathbb{E}[f(D_t)|\theta_{t,a}] - \mathbb{E}[f(D_t)|\theta_{t,b}]$ using Equation (12). For each guide t, we calculate the 2.5% and 97.5% quantile of the posterior distribution of Δ_t (approximated from the MCMC samples). The difference criterion is satisfied for targeting guides t for which the interval between these quantiles excludes zero.

2. **Biological magnitude criterion**
   For each control guide c, calculate the semi-interquartile range of the posterior distribution of Δ_c (i.e., half of the difference between the 25% and 75% quantile values). We use the mean semi-interquartile range of all control guides as a threshold below which a fitness difference between targeting guides is considered not to be of biological significance. The difference criterion is satisfied for targeting guides t for which the absolute value of the median of the posterior distribution of Δ_t exceeds this biological significance threshold.

3. **Rank difference criterion**
   Let ρ_t denote a random variable equal to the rank of guide t when the guides are sorted by their Winsorized mean fitness. When two experimental conditions are considered, denote the corresponding ranks by $\rho_{t,a}$ and $\rho_{t,b}$. Let $\delta_t = \rho_{t,a} - \rho_{t,b}$. For each guide t, we calculate the 2.5% and 97.5% quantile of the posterior distribution of δ_t (approximated from the MCMC samples). The difference criterion is satisfied for targeting guides t for which the interval between these quantiles excludes zero.

4. **Multiple guides** The difference criterion is satisfied for guides targeting a gene for which at least 2 guides satisfy all of criteria 1, 2, and 3.

1.9 Comparison with cell line essentiality dataset We downloaded the Sanger Institute cell line CRISPR screen dataset reported in [20]. We extracted the proportion of breast cancer cell lines (n=25) with fitness dependency on each gene (ADaM adaptive daisy model). For each of the 56 genes in our signaling library, we calculated the median reported depletion of CRISPRcleanR-corrected sgRNAs-level values (BAGEL model) across the 25 breast cell lines. We compared the rank ordering of genes based on this in vitro median fitness metric with the rank ordering of genes based on median in vivo fitness in our PDX datasets (15 PDX lines used).
