# Supplementary material for: Accurate determination of CRISPR-mediated gene fitness in transplantable tumours
Source: Nat Commun. 2022 Aug 4;13:4534. doi: 10.1038/s41467-022-31830-2 (PMC9352714; doi:10.1038/s41467-022-31830-2)
Supplement: Supplementary file 2 — Description to Additional Supplementary Information [file 41467_2022_31830_MOESM2_ESM.pdf]

Supplementary Data 1

Bayesian mix-NB model results for *in vivo* pooled CRISPR screens in 181 PDX tumours, reported as median fitness and 95% credible intervals for each guide.
