# Supplementary material for: Accurate determination of CRISPR-mediated gene fitness in transplantable tumours
Source: Nat Commun. 2022 Aug 4;13:4534. doi: 10.1038/s41467-022-31830-2 (PMC9352714; doi:10.1038/s41467-022-31830-2)
Supplement: Supplementary file 4 — Reporting Summary [file 41467_2022_31830_MOESM4_ESM.pdf]

nature research

Corresponding author(s): Dr Samuel Aparicio

Last updated by author(s): June 21 2022

Reporting Summary

Nature Research wishes to improve the reproducibility of the work that we publish. This form provides structure for consistency and transparency in reporting. For further information on Nature Research policies, see our Editorial Policies and the Editorial Policy Checklist.

Statistics

For all statistical analyses, confirm that the following items are present in the figure legend, table legend, main text, or Methods section.

n/a	Confirmed	
☐	☒	The exact sample size (n) for each experimental group/condition, given as a discrete number and unit of measurement
☐	☒	A statement on whether measurements were taken from distinct samples or whether the same sample was measured repeatedly
☐	☒	The statistical test(s) used AND whether they are one- or two-sided<br>*Only common tests should be described solely by name; describe more complex techniques in the Methods section.*
☐	☒	A description of all covariates tested
☐	☒	A description of any assumptions or corrections, such as tests of normality and adjustment for multiple comparisons
☐	☒	A full description of the statistical parameters including central tendency (e.g. means) or other basic estimates (e.g. regression coefficient) AND variation (e.g. standard deviation) or associated estimates of uncertainty (e.g. confidence intervals)
☐	☒	For null hypothesis testing, the test statistic (e.g. F, t, r) with confidence intervals, effect sizes, degrees of freedom and P value noted<br>*Give P values as exact values whenever suitable.*
☐	☒	For Bayesian analysis, information on the choice of priors and Markov chain Monte Carlo settings
☐	☒	For hierarchical and complex designs, identification of the appropriate level for tests and full reporting of outcomes
☐	☒	Estimates of effect sizes (e.g. Cohen's d, Pearson's r), indicating how they were calculated

Our web collection on statistics for biologists contains articles on many of the points above.

Software and code

Policy information about availability of computer code

Data collection	FASTQ files were generated by Illumina control software on Illumina MiSeq and NextSeq benchtop sequencing machines. MiSeq currently running Illumina Control Software version 4.0.0.1769.
Data analysis	Fitness modeling used the Blang probabilistic programming language. The model code and sample datasets are available on Github (https://github.com/aroth85/humi_pipeline). Blang code is available at \url{https://github.com/UBC-Stat-ML/nowellpack. Pre- and post-processing of results used custom scripts in Python and R version 3.6.3. Mutation-calling algorithms mutationSeq, TITAN, deStruct and signature inference MMCTM are published, and cited in the text. ClinVar (https://www.ncbi.nlm.nih.gov/clinvar/) and COSMIC (https://cancer.sanger.ac.uk/cosmic) datasets were used for mutation annotation. The CHOPCHOP webtool (version 3, https://chopchop.cbu.uib.no) was used for CRISPR guide design. Flow cytometry data was analyzed using FloJo 10.7.1.

For manuscripts utilizing custom algorithms or software that are central to the research but not yet described in published literature, software must be made available to editors and reviewers. We strongly encourage code deposition in a community repository (e.g. GitHub). See the Nature Research guidelines for submitting code & software for further information.

Data

Policy information about availability of data

All manuscripts must include a data availability statement. This statement should provide the following information, where applicable:

- Accession codes, unique identifiers, or web links for publicly available datasets
- A list of figures that have associated raw data
- A description of any restrictions on data availability

Targeted sequence, whole genome sequence and RNAseq data have been deposited in the NCBI Sequence Read Archive (SRA) under BioProject ID PRJNA842677.

Derived UMI-sgRNA count files have been deposited at Zenodo DOI: 10.5281/zenodo.6584802. Mutation annotation was carried out using ClinVar (https://www.ncbi.nlm.nih.gov/clinvar/) and COSMIC (https://cancer.sanger.ac.uk/cosmic). Source data are provided for all figures as Source Data files.

Field-specific reporting

Please select the one below that is the best fit for your research. If you are not sure, read the appropriate sections before making your selection.

☒ Life sciences ☐ Behavioural & social sciences ☐ Ecological, evolutionary & environmental sciences

For a reference copy of the document with all sections, see nature.com/documents/nr-reporting-summary-flat.pdf

Life sciences study design

All studies must disclose on these points even when the disclosure is negative.

Sample size	The number of patient-derived xenograft lines used in the study (n=21) was chosen to ensure coverage of initiating patient tumours with a variety of histological subtypes (HR+, HR+Her2+, Her2+, TNBC), site (breast or metastatic) and including treatment-naive and previously therapy-treated at the time of biopsy. Only tumours that successfully engrafted in mice to were used, which represents some bias in favour of more aggressive tumours within each histological subtype.
Data exclusions	No data was excluded. In analyses that used subsets of the data, the selection criteria are detailed in the main text or supplementary methods sections.
Replication	Transplantation experiments were routinely carried out as biological replicated into 3-4 recipient mice. Replication was successful in a large majority of cases, with occasional instances in which a replicate transplant did not generate a palpable tumour. This is a normal occurrence when xenografting human tissue.
Randomization	Mice were randomized for drug treatment experiments and for experiments testing different transplant sites.
Blinding	Blinding was not relevant to the study, as the measured endpoint (sequence data from individual tumours) was considered free from subjective bias.

Reporting for specific materials, systems and methods

We require information from authors about some types of materials, experimental systems and methods used in many studies. Here, indicate whether each material, system or method listed is relevant to your study. If you are not sure if a list item applies to your research, read the appropriate section before selecting a response.

Materials & experimental systems

n/a	Involved in the study	
☒	☐	Antibodies
☐	☒	Eukaryotic cell lines
☒	☐	Palaeontology and archaeology
☐	☒	Animals and other organisms
☐	☒	Human research participants
☒	☐	Clinical data
☒	☐	Dual use research of concern

Methods

n/a	Involved in the study	
☒	☐	ChIP-seq
☐	☒	Flow cytometry
☒	☐	MRI-based neuroimaging

Eukaryotic cell lines

Policy information about cell lines

Cell line source(s)	MCF-7 from ATCC. HEK293T from ATCC). 184hTERT-L9 and 184hTERT p53-/- BRCA2-/- derived from parental 184hTERT, a gift from Martha Stampfer.
Authentication	MCF-7, HEK293T cells were not authenticated. 184hTERT lines were authenticated by sequencing.
Mycoplasma contamination	Cell lines tested negative for mycoplasma contamination.
Commonly misidentified lines (See ICLAC register)	None

Animals and other organisms

Policy information about studies involving animals; ARRIVE guidelines recommended for reporting animal research

Laboratory animals	Mice, NOD/SCID/IL2rgamma-/- (NSG) and NOD/Rag1-/-/Il2rgamma-/- (NRG), female, transplanted at 5-12 weeks of age
Wild animals	Study did not involve wild animals.
Field-collected samples	Study did not involve samples collected from the field.
Ethics oversight	All animal experimental procedures were approved by the University of British Columbia Animal Care Committee.

Note that full information on the approval of the study protocol must also be provided in the manuscript.

Human research participants

Policy information about studies involving human research participants

Population characteristics	Breast cancer patients, female, age 35-86, undergoing surgery or diagnostic core biopsy.
Recruitment	Patients were recruited with informed consent by breast oncologists working on this study. Due to the research focus on triple negative breast cancer, patients with this subtype of tumour were preferentially recruited.
Ethics oversight	Ethics oversight was by the University of British Columbia Research Ethics Board.

Note that full information on the approval of the study protocol must also be provided in the manuscript.

Flow Cytometry

Plots

Confirm that:

☒ The axis labels state the marker and fluorochrome used (e.g. CD4-FITC).

☒ The axis scales are clearly visible. Include numbers along axes only for bottom left plot of group (a 'group' is an analysis of identical markers).

☒ All plots are contour plots with outliers or pseudocolor plots.

☒ A numerical value for number of cells or percentage (with statistics) is provided.

Methodology

Sample preparation	For titration of viral concentration, 184htert-L9 cells transduced with sgRNA-UMI vector were harvested from culture using trypsin/EDTA, and incubated with DAPI to distinguish viable from non-viable cells. For testing the effect of UMI addition to lentiCRISPRv2 vector sequence, eGFP-expressing HEK293T cells transfected with vectors were harvested from culture using trypsin/EDTA, and incubated with propidium iodide (PI) to distinguish viable from non-viable cells.
Instrument	LSRFortessa, BD Biosciences
Software	FlowJo, BD
Cell population abundance	No cell sorting was carried out.
Gating strategy	Cell population were successively gated to exclude non-viable cells (forward scatter low, DAPI- or PI-), then to exclude side scatter high cells. A final gate (forward scatter vs dsRed, or forward scatter vs eGFP was determined with reference to a non-vector-transduced control cell population.

☒ Tick this box to confirm that a figure exemplifying the gating strategy is provided in the Supplementary Information.
