# Supplementary figures and images for: Accurate determination of CRISPR-mediated gene fitness in transplantable tumours
Source: Nat Commun. 2022 Aug 4;13:4534. doi: 10.1038/s41467-022-31830-2 (PMC9352714; doi:10.1038/s41467-022-31830-2)

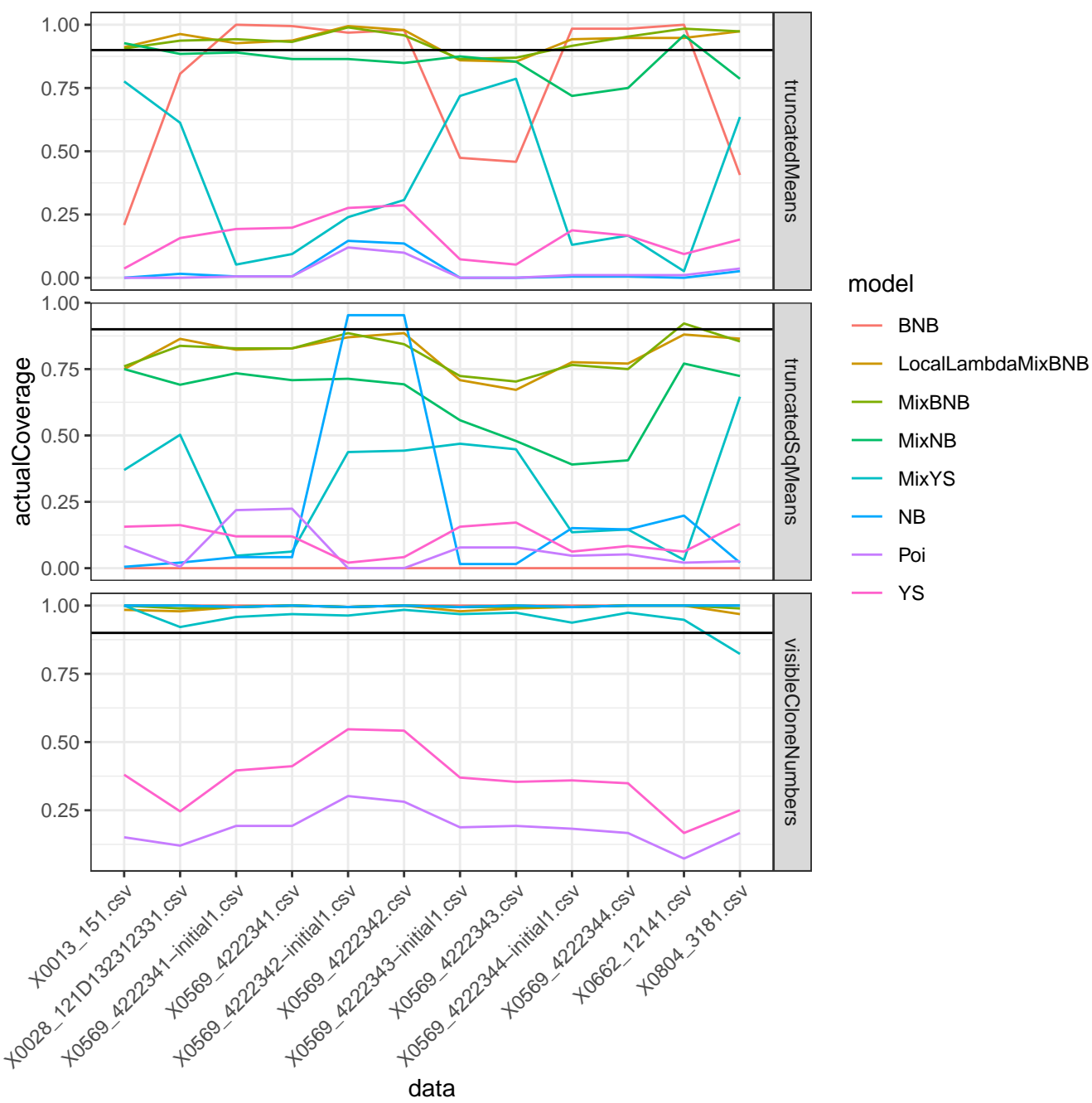

Supplement: Supplementary file 5 — Source data [file 41467_2022_31830_MOESM5_ESM.zip › source_data/FigS15/gof.pdf]

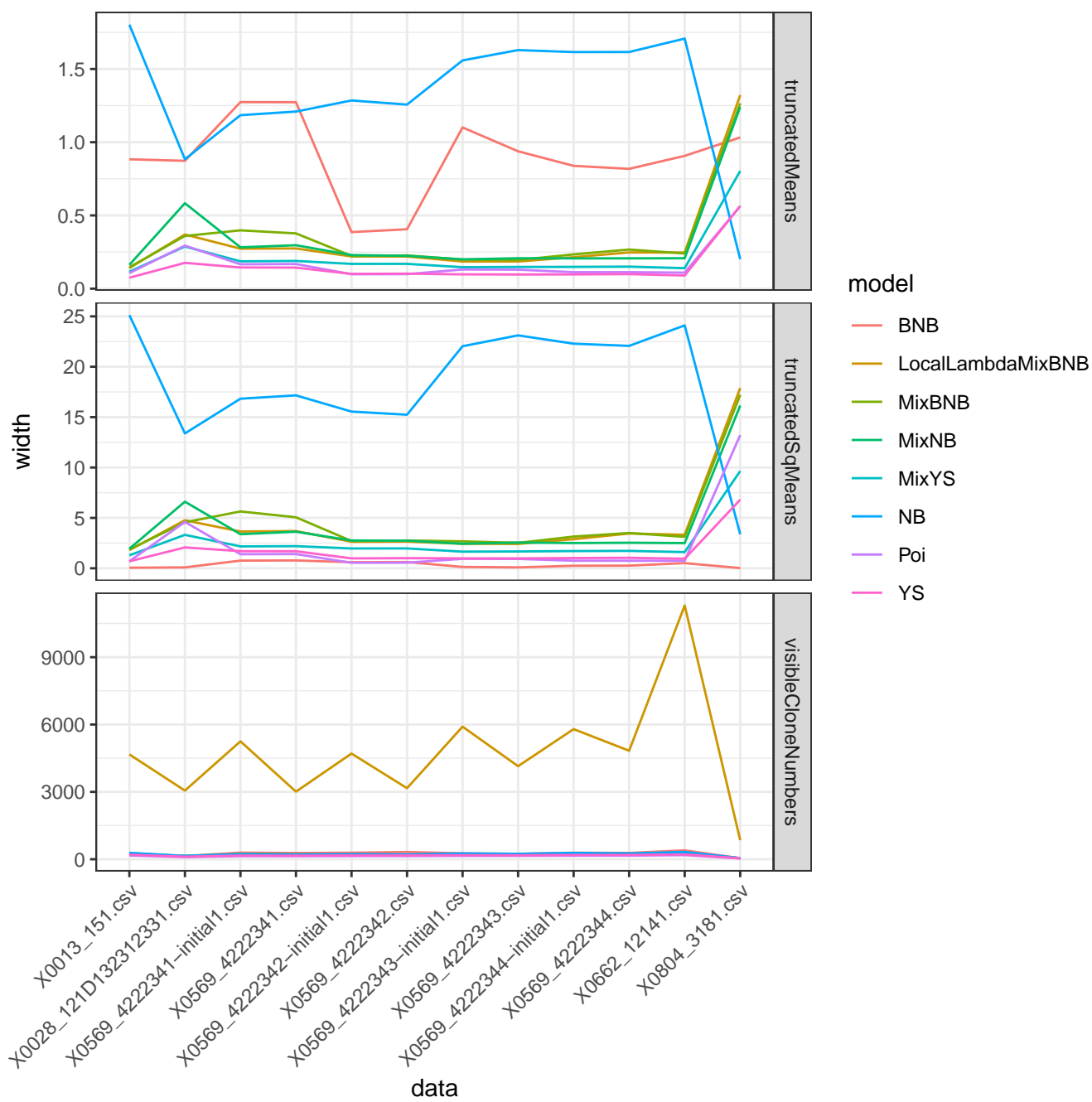

Supplement: Supplementary file 5 — Source data [file 41467_2022_31830_MOESM5_ESM.zip › source_data/FigS15/width.pdf]

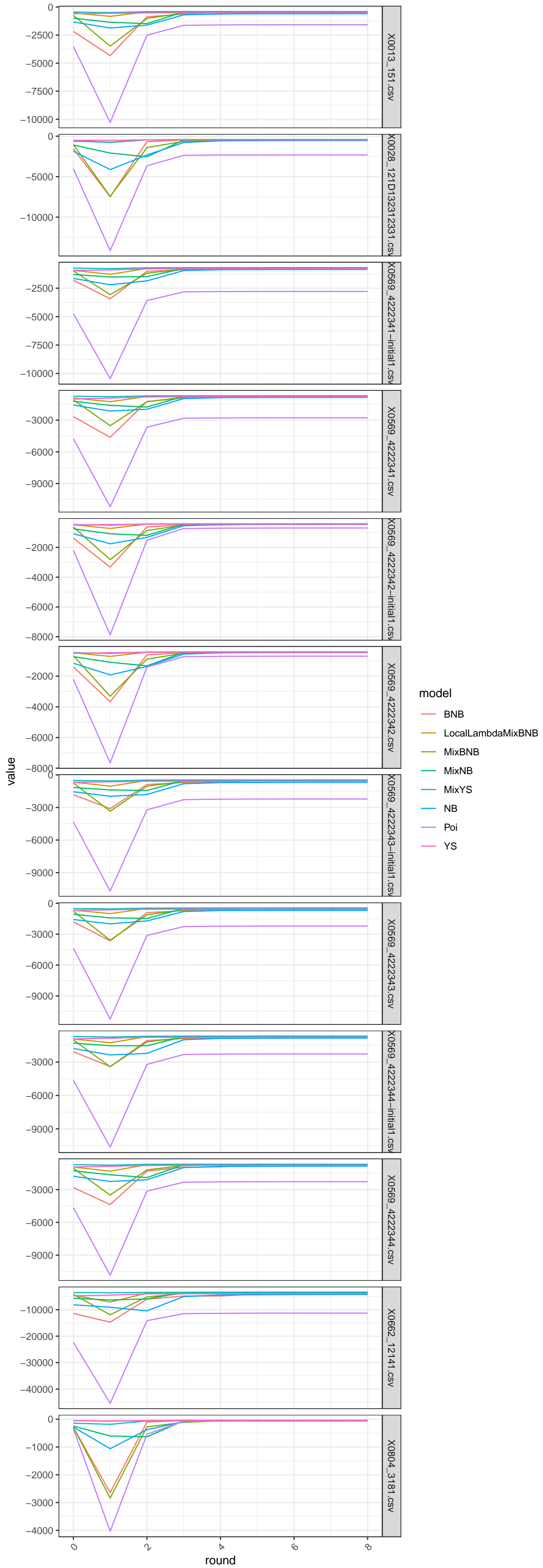

Supplement: Supplementary file 5 — Source data [file 41467_2022_31830_MOESM5_ESM.zip › source_data/FigS16/evidence.pdf]

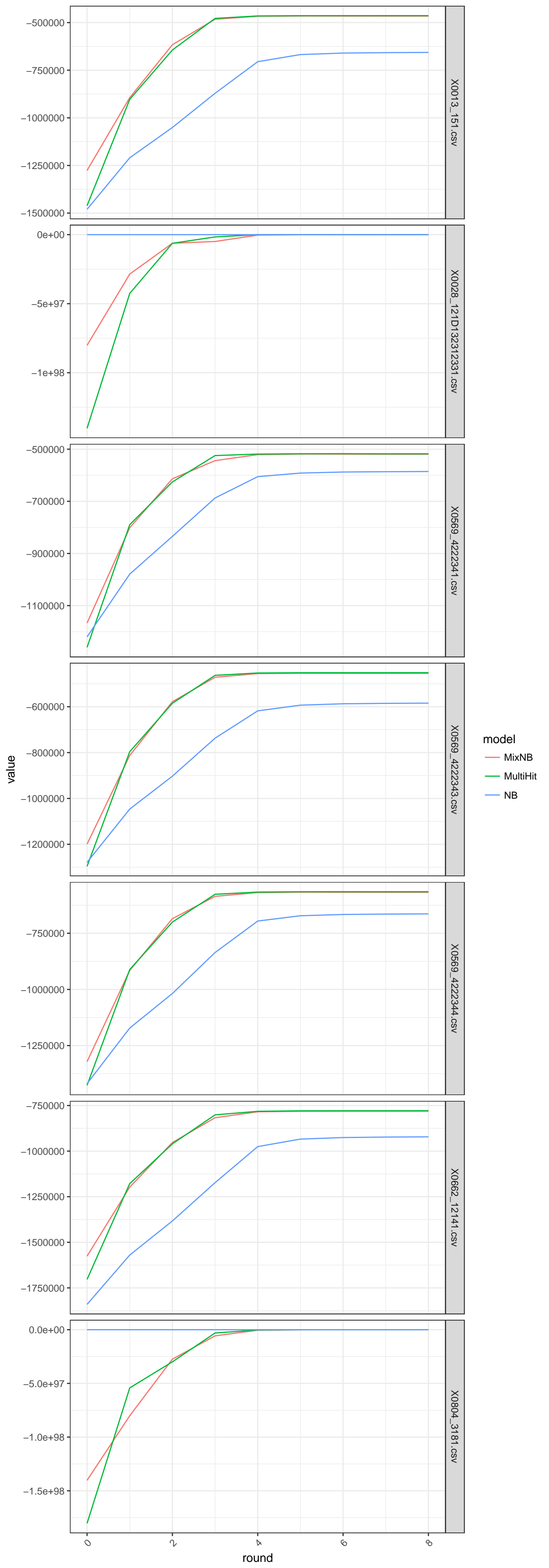

Supplement: Supplementary file 5 — Source data [file 41467_2022_31830_MOESM5_ESM.zip › source_data/FigS17/evidence2.pdf]

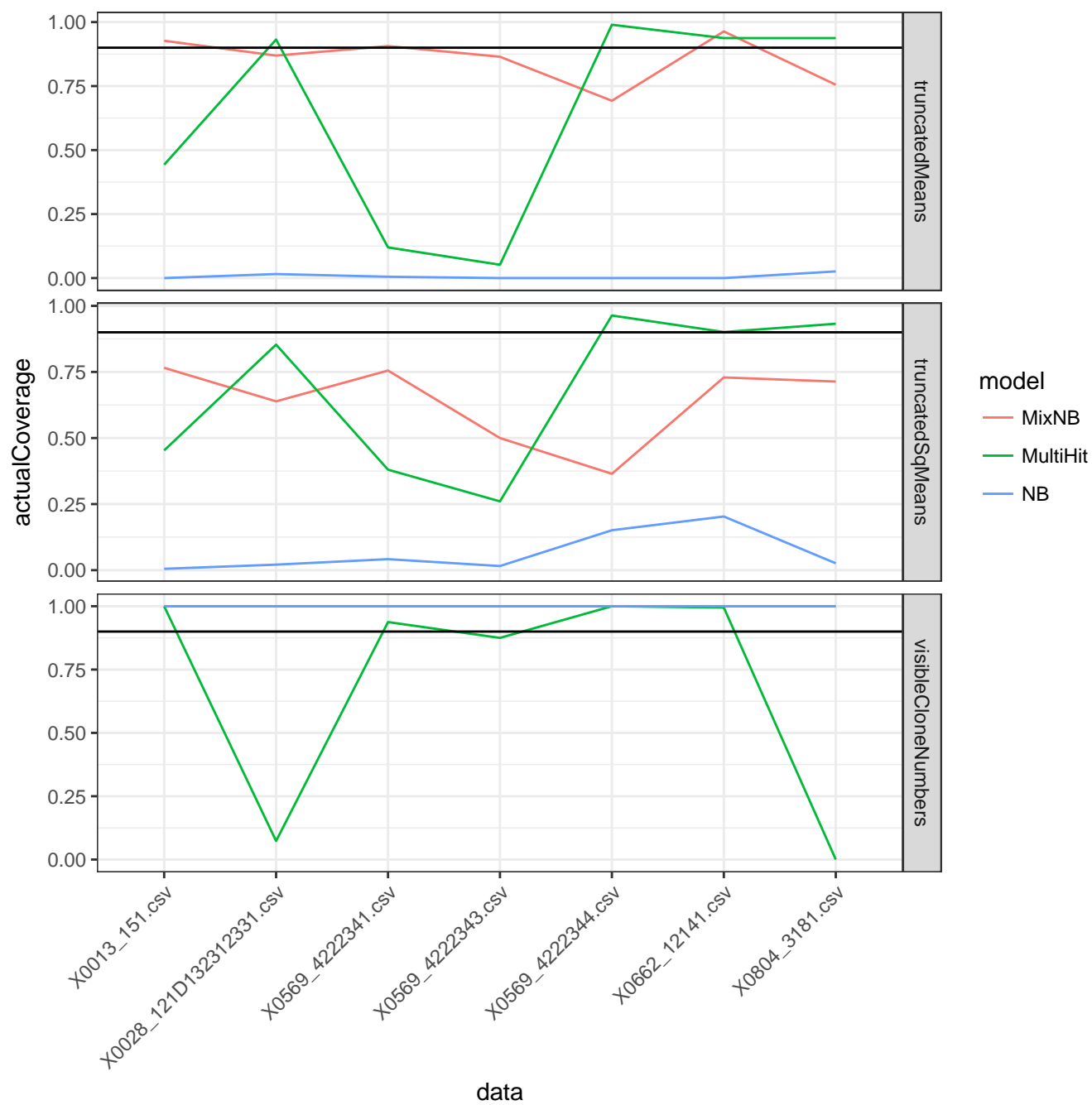

Supplement: Supplementary file 5 — Source data [file 41467_2022_31830_MOESM5_ESM.zip › source_data/FigS17/gof.pdf]

Ratio of clone sizes relative to controls  
Bayesian hierarchical model credible intervals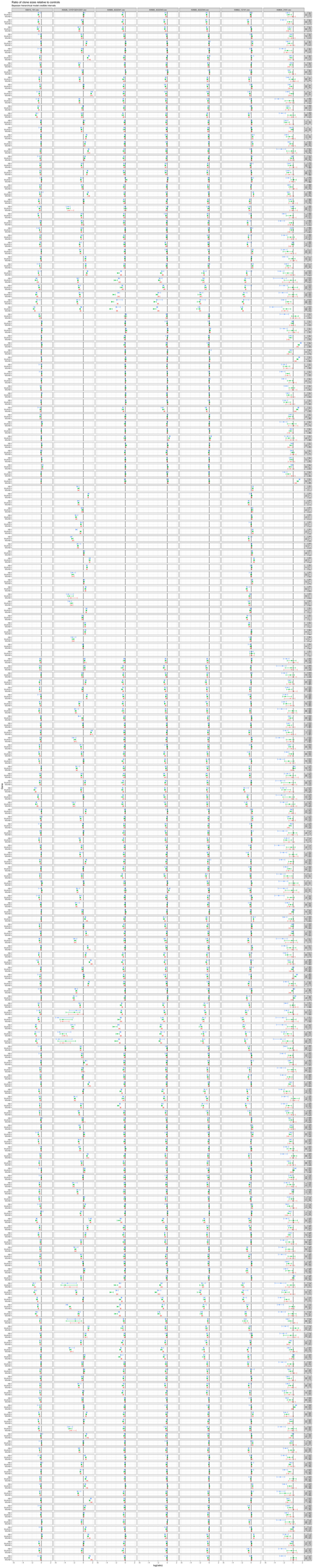

Supplement: Supplementary file 5 — Source data [file 41467_2022_31830_MOESM5_ESM.zip › source_data/FigS17/intervals-multi.pdf]

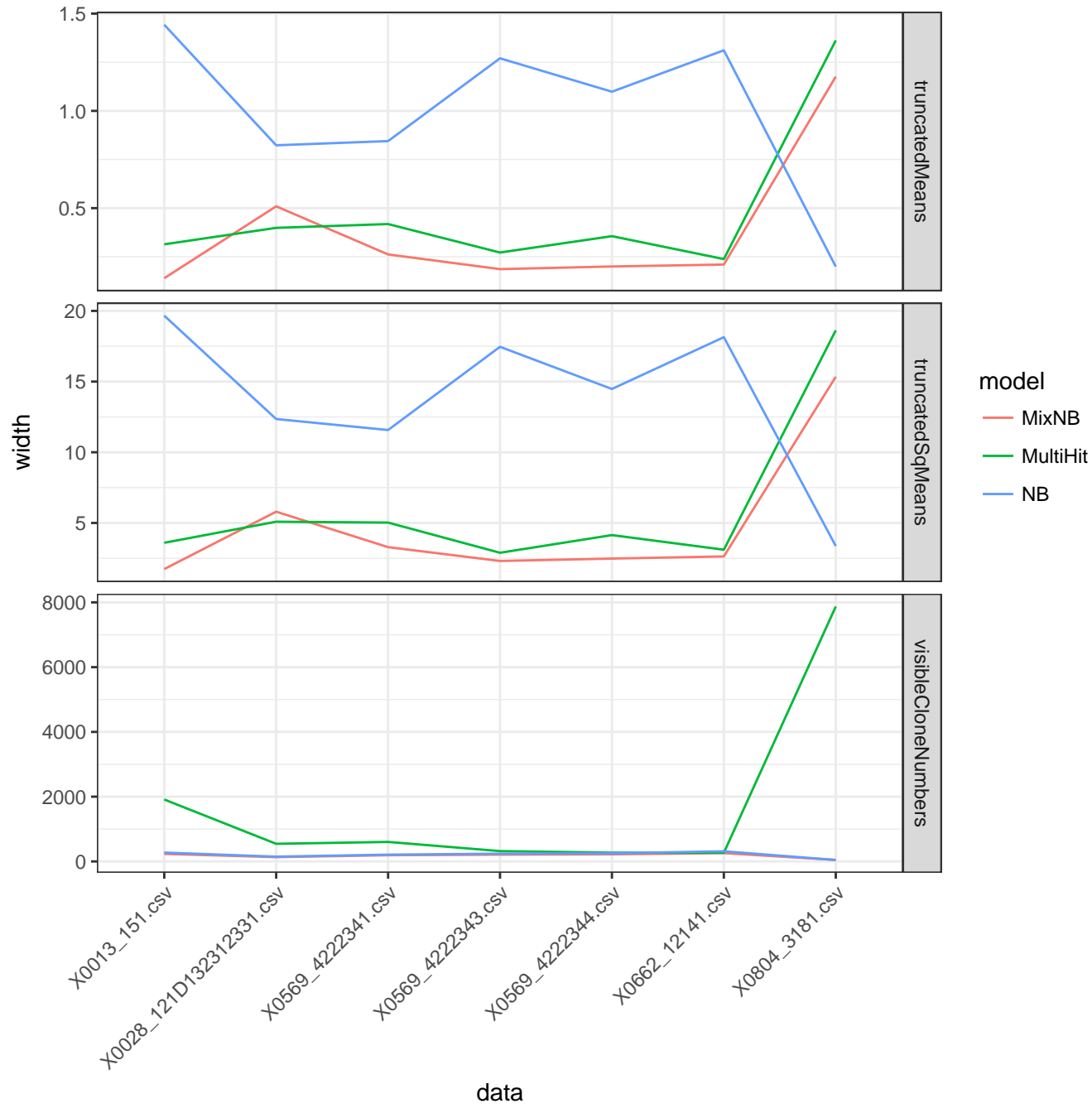

Supplement: Supplementary file 5 — Source data [file 41467_2022_31830_MOESM5_ESM.zip › source_data/FigS17/width.pdf]
