# Supplementary material for: Accurate determination of CRISPR-mediated gene fitness in transplantable tumours
Source: Nat Commun. 2022 Aug 4;13:4534. doi: 10.1038/s41467-022-31830-2 (PMC9352714; doi:10.1038/s41467-022-31830-2)
Supplement: Supplementary file 5 — Source data [file intervals-sensitivity.pdf › source_data/FigS18/intervals-sensitivity.pdf]

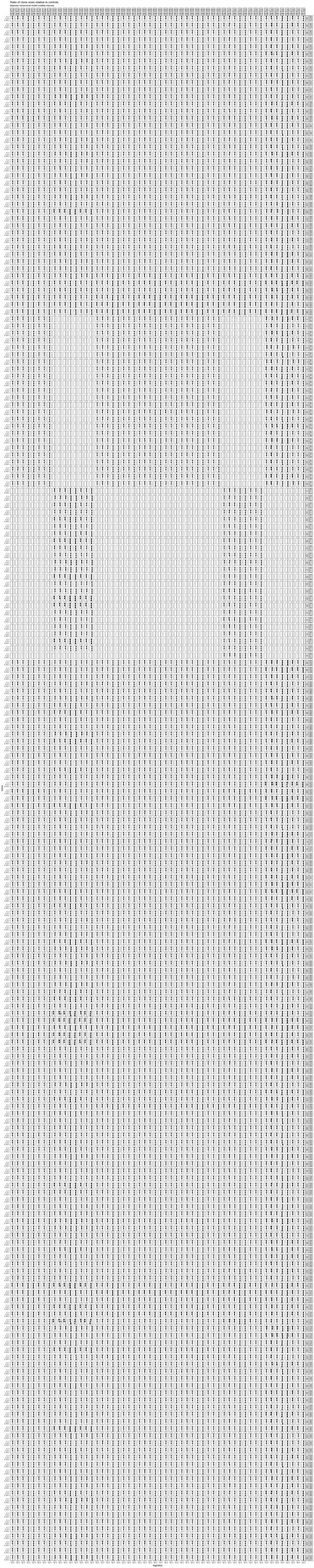
Ratio of clone sizes relative to controls
Bayesian hierarchical model credible intervals
Gene
log(ratio)
